# Supplementary material for: S-acylation of p62 promotes p62 droplet recruitment into autophagosomes in mammalian autophagy
Source: Mol Cell. 2023 Oct 5;83(19):3485–3501.e11. doi: 10.1016/j.molcel.2023.09.004 (PMC10552648; doi:10.1016/j.molcel.2023.09.004)
Supplement: Document S1. Figures S1–S7 [file mmc1.pdf]

Molecular Cell, Volume *83*

Supplemental information

S-acylation of p62 promotes p62 droplet recruitment into autophagosomes in mammalian autophagy

Xue Huang, Jia Yao, Lu Liu, Jing Chen, Ligang Mei, Jingjing Huangfu, Dong Luo, Xinyi Wang, Changhai Lin, Xiaorong Chen, Yi Yang, Sheng Ouyang, Fujing Wei, Zhuolin Wang, Shaolin Zhang, Tingxiu Xiang, Dante Neculai, Qiming Sun, Eryan Kong, Edward W. Tate, and Aimin Yang

Supplemental Information

S-acylation of p62 promotes p62 droplet recruitment into autophagosomes in mammalian autophagy

Xue Huang, Jia Yao, Lu Liu, Jing Chen, Ligang Mei, Jingjing Huangfu, Dong Luo, Xinyi Wang, Changhai Lin, Xiaorong Chen, Yi Yang, Sheng Ouyang, Fujing Wei, Zhuolin Wang, Shaolin Zhang, Tingxiu Xiang, Dante Neculai, Qiming Sun, Eryan Kong, Edward W. Tate, and Aimin Yang

This file includes:

- Supplementary Figures and Legends
  - Figure S1 to S7

Supplemental Figures

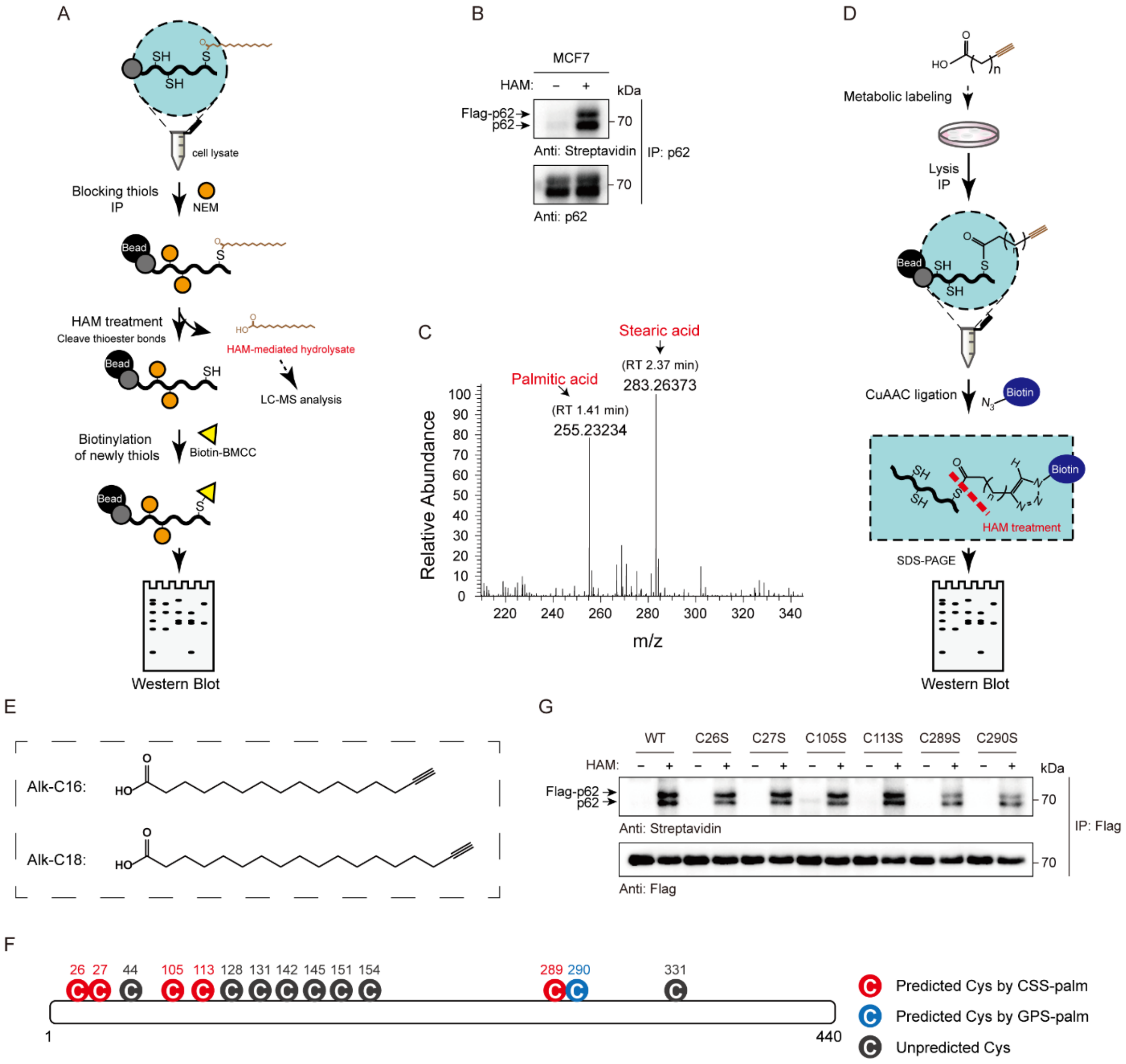

Figure S1. p62 undergoes *S*-acylation at Cys289 and Cys290, related to Figure 1

(**A**) Scheme of the acyl-biotin exchange (ABE) assay. Cell lysates are treated with *N*-ethylmaleimide (NEM) to irreversibly block free thiol (–SH) groups, and a target protein is then purified using immunoprecipitation. The purified target protein is subjected to treatment with Hydroxylamine (HAM) to expose the thiol groups of acylated cysteines and then reacts with a thiol-reactive biotin molecule, biotin-BMCC, resulting in specific biotinylation of the acylated cysteines.

(**B**) *S*-acylation of Flag-tagged p62 and endogenous p62 detected by ABE assay in MCF7 cells. Cells were transfected with the plasmid encoding Flag-tagged p62. Flag-tagged p62 and endogenous p62 were immunoprecipitated with anti-p62 antibody, and *S*-acylated p62 was further detected by the ABE assay.

(**C**) Mass spectrometry analysis of HAM-mediated hydrolysates. The HAM-mediated hydrolysates from MCF7 cells as in (**B**) were analyzed by mass spectrometry. Stearic acid and palmitic acid were detected in the HAM-mediated hydrolysates in negative ion mode. HRMS: stearic acid, *m/z*: calcd for $C_{18}H_{35}O_2$ 283.26371 $[M\text{-}H]^-$, found 283.26373; palmitic acid, *m/z*: calcd for $C_{16}H_{31}O_2$ 255.23241 $[M\text{-}H]^-$, found 255.23234.

(**D**) Scheme of chemical reporters for protein acylation and the click reaction. Cells are metabolically labeled with alkyne-functionalized lipid analogs. The on-beads copper (I)-catalyzed azide alkyne cycloaddition (CuAAC) click reaction is conducted with azide-tagged biotin (Azide-PEG3-Biotin Conjugate). HAM treatment hydrolyzes the thioester bonds, resulting in loss of biotinylation.

(**E**) Structure of Alk-C16 and Alk-C18. Alk-C16, palmitic acid alkyne. Alk-C18, stearic acid alkyne.

(**F**) Prediction of p62 acylation sites using prediction algorithms.

(**G**) *S*-acylation of Flag-p62 mutants in MCF7 cells detected by the ABE assay. Because of p62 oligomerization, anti-Flag antibody precipitated exogenous Flag-p62 (the upper band) as well as p62 (the lower band).

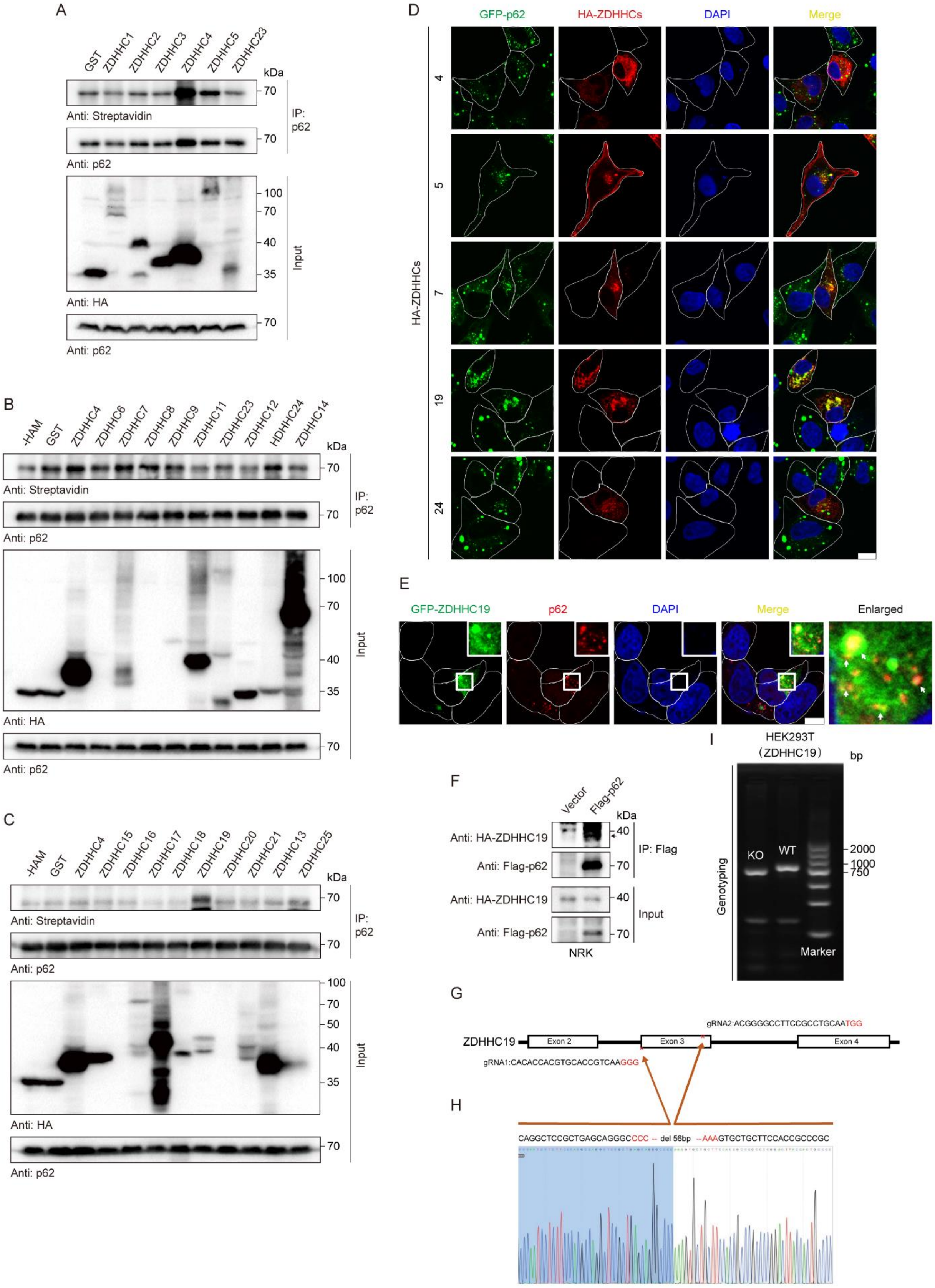
A
GST ZDHHC1 ZDHHC2 ZDHHC3 ZDHHC4 ZDHHC5 ZDHHC23
kDa
Anti: Streptavidin
Anti: p62
IP: p62
Anti: HA
Input
Anti: p62
B
-HAM GST ZDHHC4 ZDHHC6 ZDHHC7 ZDHHC8 ZDHHC9 ZDHHC11 ZDHHC23 ZDHHC12 HDHHC24 ZDHHC14
C
-HAM GST ZDHHC4 ZDHHC15 ZDHHC16 ZDHHC17 ZDHHC18 ZDHHC19 ZDHHC20 ZDHHC21 ZDHHC13 ZDHHC25
D
GFP-p62 HA-ZDHHCs DAPI Merge
HA-ZDHHCs
4 5 7 19 24
E
GFP-ZDHHC19 p62 DAPI Merge Enlarged
F
Vector Flag-p62
Anti: HA-ZDHHC19
Anti: Flag-p62
IP: Flag
Input
NRK
I
HEK293T (ZDHHC19)
bp
KO WT
Marker
Genotyping
2000 1000 750
G
gRNA2:ACGGGGCCTTCCGCCTGCAATGG
ZDHHC19
Exon 2 Exon 3 Exon 4
gRNA1:CACACCACGTGCACCGTCAAGGG
H
CAGGCTCCGCTGAGCAGGGCCCC -- del 56bp -- AAAGTGCTGCTTCCACCGCCCGC

Figure S2. ZDHHC19 mediates p62 *S*-acylation, related to Figure 2

(**A**), (**B**) and (**C**) p62 *S*-acylation in cells expressing ZDHHCs. MCF7 cells were transfected with HA-ZDHHC1-25 plasmids to screen for acyltransferases that could increase p62 acylation. Endogenous p62 was immunoprecipitated with anti-p62 antibody, and *S*-acylated p62 was further analyzed by ABE assay. Key ZDHHC candidates were replicated to examine the acylation level.

(**D**) Representative images of the colocalization of p62 with ZDHHCs. *p62*-KO NRK cells stably expressing GFP-p62 (green) were transfected with HA-ZDHHC4, 5, 7, 19 or 24 and immunostained with anti-HA antibody (red). Scale bar, 10 μm.

(**E**) Representative images of the colocalization of stable ZDHHC19 and endogenous p62. HEK293T cells stably expressing GFP-ZDHHC19 (green) were immunostained with anti-p62 (red) antibody. Scale bar, 10 μm.

(**F)** Immunoblot analysis of Flag-p62-immunoprecipitated (IP) proteins in NRK cells co-expressing Flag-p62 with HA-ZDHHC19.

(**G**) Deleting *ZDHHC19* in HEK293T cells. Targeting scheme of the truncation of exon 3 in human *ZDHHC19*. Two sgRNAs were designed to target the enzyme activity center (ACGGGGCCTTCCGCCTGCAA, CACACCACGTGCACCGTCAA) of human *ZDHHC19*.

(**H**) Sequencing verification of the *ZDHHC19*-KO cell line. The knockout allele has a deletion of 56 bp on exon 3, confirmed by sequencing, and the ORF finder predicts that the knockout allele results in a premature termination of the protein.

(**I**) Electrophoresis verification of the *ZDHHC19*-KO cell line. Genotyping was carried out by PCR amplification with corresponding primers and the amplicons of WT and knockout alleles were 420 bp and 364 bp, respectively.

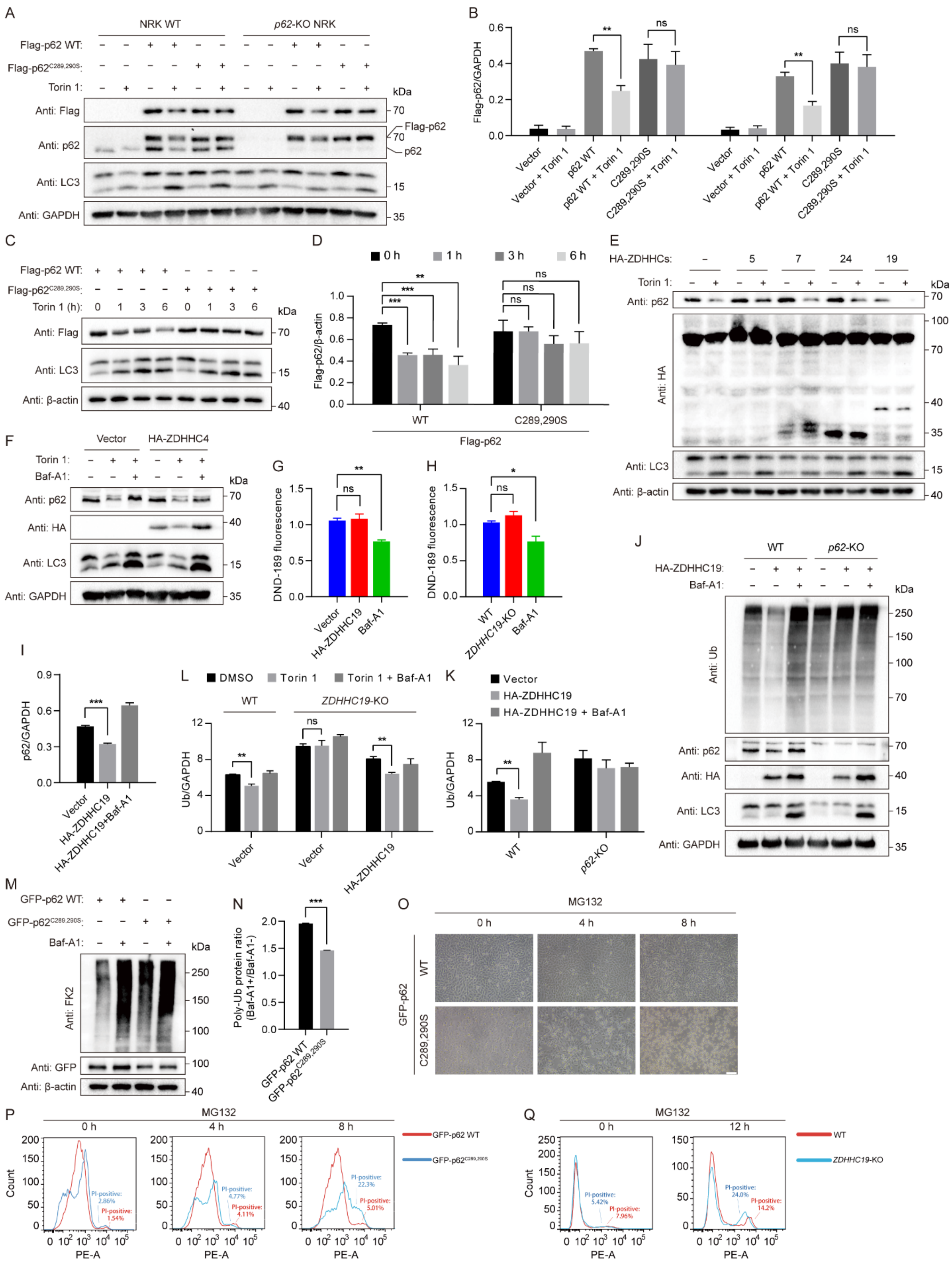

A
NRK WT
p62-KO NRK
Flag-p62 WT:
Flag-p62C289,290S:
Torin 1:
kDa
Anti: Flag
Anti: p62
Flag-p62
p62
Anti: LC3
Anti: GAPDH
B
Flag-p62/GAPDH
Vector
Vector + Torin 1
p62 WT
p62 WT + Torin 1
C289,290S
C289,290S + Torin 1
C
Torin 1 (h):
Anti: β-actin
D
Flag-p62/β-actin
WT
C289,290S
Flag-p62
E
HA-ZDHHCs:
Anti: HA
F
Vector
HA-ZDHHC4
Baf-A1:
G
DND-189 fluorescence
HA-ZDHHC19
H
ZDHHC19-KO
I
p62/GAPDH
HA-ZDHHC19+Baf-A1
J
Anti: Ub
L
DMSO
Torin 1 + Baf-A1
Ub/GAPDH
K
p62-KO
M
GFP-p62 WT:
GFP-p62C289,290S:
Anti: FK2
Anti: GFP
N
Poly-Ub protein ratio (Baf-A1+/Baf-A1-)
O
MG132
GFP-p62
P
Count
PE-A
PI-positive
Q

Figure S3. *S*-acylation facilitates the degradation of p62 and ubiquitinated proteins, related to Figure 3

(**A**) Immunoblot analysis of Flag-p62 WT and Flag-p62^{C289,290S} proteins in NRK WT or *p62*-KO cells. Cells were transiently transfected with Flag-p62 WT or Flag-p62^{C289,290S} under basal condition or upon induction of autophagy with the mTOR inhibitor Torin 1 (1 μM) for 1 h. Total proteins were extracted and detected by Western blotting.

(**B**) Quantification of Flag-p62 WT and Flag-p62^{C289,290S} protein levels as in (**A**). Data represent the mean ± SEM of three independent experiments. "ns", no significant difference, $^{**}P < 0.01$, Student's *t* test.

(**C**) Immunoblot analysis of Flag-p62 WT and Flag-p62^{C289,290S} upon autophagy induction. *p62*-KO NRK cells were transfected with Flag-p62 WT or Flag-p62^{C289,290S}, followed by Torin 1 (1 μM) treatment for the indicated time points.

(**D**) Quantification of Flag-p62 WT and Flag-p62^{C289,290S} protein levels as in (**C**). Data represent the mean ± SEM of three independent experiments. "ns", no significant difference, $^{**}P < 0.01$, $^{***}P < 0.001$, Student's *t* test.

(**E**) Immunoblot analysis of p62 protein level upon the expression of each ZDHHC. NRK cells were transfected with HA-ZDHHC5, 7, 24 or 19 and treated with DMSO or Torin 1 (1 μM) for 1 h.

(**F**) Immunoblot analysis of p62 protein level upon ZDHHC4 expression. NRK cells were transfected with empty vector or HA-ZDHHC4, and treated with Torin 1 (1 μM) for 1 h with or without Baf-A1 (1 μM). Baf-A1, Bafilomycin A1.

(**G**) Analysis of lysosomal pH in HEK293T cells expressing HA-ZDHHC19. The HEK293T cells transiently expressing empty vector or HA-ZDHHC19 were incubated with DND-189 (1 μM) for 1 h. Baf-A1 was used as positive control. Mean intensity of fluorescence was detected by flow cytometry and assessed by FlowJo software. Data represent the mean ± SEM of three independent experiments. "ns", no significant difference, $^{**}P < 0.01$, Student's *t* test.

(**H**) Analysis of lysosomal pH in WT or *ZDHHC19*-KO HEK293T cells. The WT or *ZDHHC19*-KO HEK293T cells were incubated with DND-189 (1 μM) for 1 h. Data represent the mean ± SEM of three independent experiments. "ns", no significant difference, $^{*}P < 0.05$, Student's *t* test.

(**I**) Quantification of p62 protein levels as in **Figure 3K**. Data represent the mean ± SEM of three independent experiments. ***P < 0.001, Student's *t* test.

(**J**) Immunoblot analysis of total ubiquitinated protein and p62 protein level upon ZDHHC19 expression. WT or *p62*-KO NRK cells were transiently transfected with HA-ZDHHC19 and treated with DMSO or Baf-A1 (1 μM) for 6 h.

(**K**) Quantification of total ubiquitinated proteins as in (**J**). Data represent the mean ± SEM of three independent experiments. **P < 0.01, Student's *t* test.

(**L**) Quantification of total ubiquitinated protein levels as in **Figure 3E**. Data represent the mean ± SEM of three independent experiments. "ns", no significant difference, **P < 0.01, Student's *t* test.

(**M**) Immunoblot analysis of total poly-ubiquitinated proteins in GFP-p62 WT and GFP-p62^{C289,290S} cells. *p62*-KO NRK cells stably expressing GFP-p62 WT or GFP-p62^{C289,290S} were treated with DMSO or Baf-A1 (1 μM) for 18 h. The levels of polyubiquitinated proteins were detected with anti-FK2 antibody.

(**N**) Quantification of total ubiquitinated proteins in Baf-A1-treated cells to that in Baf-A1-untreated cells as in (**M**). Data represent the mean ± SEM of three independent experiments. ***P < 0.001, Student's *t* test.

(**O**) Representative bright-field images of *p62*-KO NRK cells stably expressing GFP-p62 WT or GFP-p62^{C289,290S}. Cells were treated with MG132 (5 μM) for the indicated time points and observed by microscopy. Scale bar, 100 μm.

(**P**) Cell death assay of GFP-p62 WT or GFP-p62^{C289,290S} cells. *p62*-KO NRK cells stably expressing GFP-p62 WT or GFP-p62^{C289,290S} were treated with MG132 (5 μM) for the indicated time points and stained with propidium iodide (PI). The fluorescence of PI-stained cells was detected by flow cytometry and assessed by FlowJo software.

(**Q**) Cell death assay of WT or *ZDHHC19*-KO HEK293T cells. The WT or *ZDHHC19*-KO HEK293T cells were treated with MG132 (10 μM) for 8 h and stained with propidium iodide (PI).

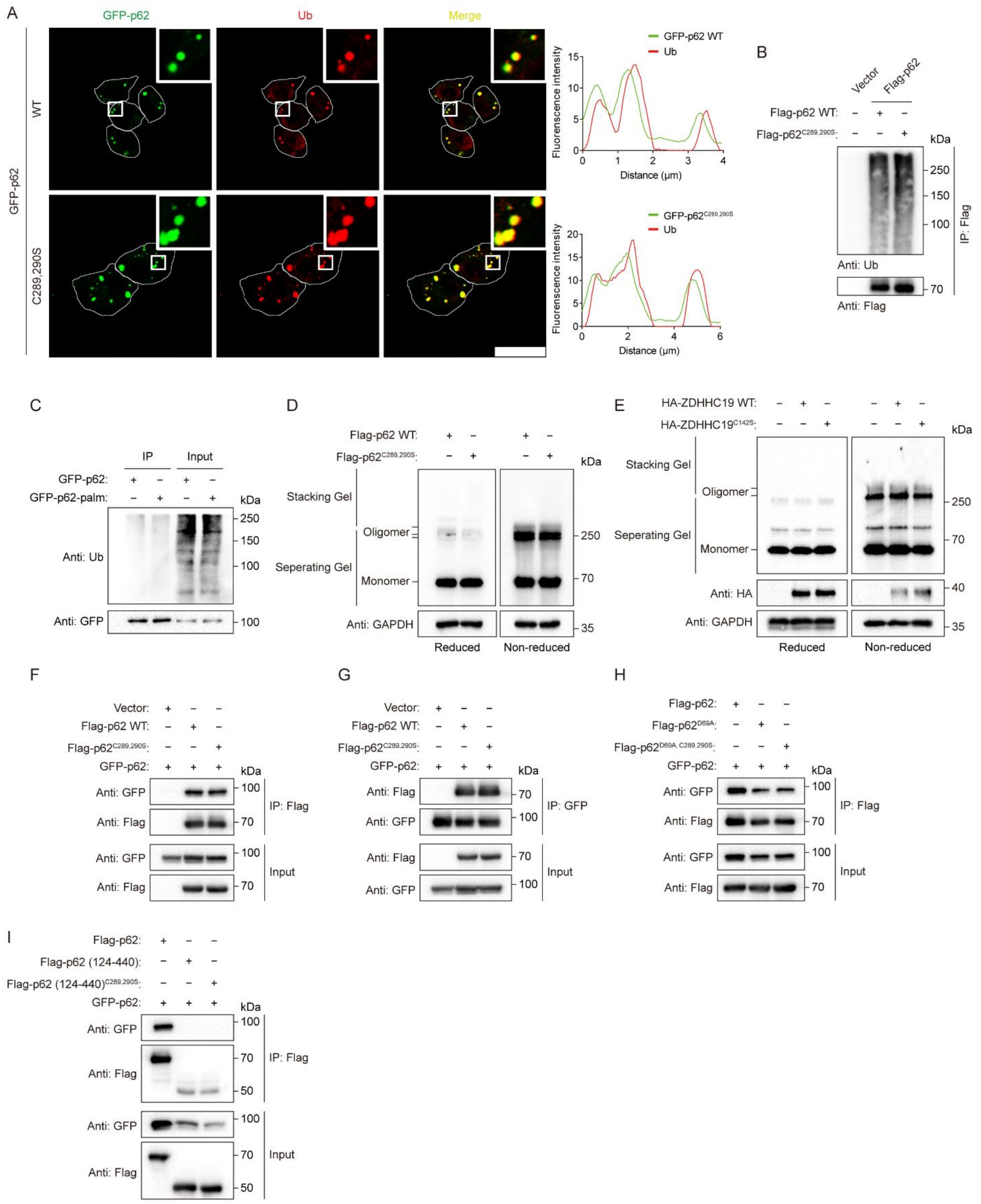

Figure S4. Effect of *S*-acylation on p62-ubiquitin binding and its oligomerization, related to Figure 4

(**A**) Representative images of colocalization of GFP-p62 and poly-ubiquitinated proteins. *p62*-KO NRK cells stably expressing GFP-p62 WT and GFP-p62^{C289,290S} (green) were immunostained with anti-FK2 antibody (red), and observed under confocal microscopy. The colocalization of GFP-p62 and poly-Ub was evaluated by ImageJ software. Scale bar, 10 μm.

(**B**) Immunoblot analysis of Flag-p62-immunoprecipitated (IP) ubiquitinated proteins in NRK cells. *p62*-KO NRK cells were transfected with empty vector (control), Flag-p62 WT or Flag-p62^{C289,290S}. Immunoprecipitation was performed with anti-Flag antibody and further analyzed by Western blotting.

(**C**) The pull-down assay of purified GFP-p62 and total ubiquitin proteins. The GFP-p62 or GFP-p62-palm was incubated with *p62*-KO NRK cell lysates for overnight. The ubiquitin was co-immunoprecipitated with anti-GFP antibody.

(**D**) Immunoblot analysis of Flag-p62 oligomerization. *p62*-KO NRK cells were transfected with Flag-p62 WT or Flag-p62^{C289,290S}. The oligomer and monomer were separated by non-reduced SDS-PAGE.

(**E**) Immunoblot analysis of p62 oligomerization upon ZDHHC19 expression. HEK293T cells were transfected with empty vector, HA-ZDHHC19 WT or HA-ZDHHC19^{C142S}. The oligomer and monomer were separated by non-reduced SDS-PAGE.

(**F**) Immunoblot analysis of Flag-p62-immunoprecipitated (IP) proteins in HEK293T cells co-expressing Flag-p62 with GFP-p62. The HEK293T cells were co-transfected with GFP-p62 and Flag-p62 (WT or the mutant p62^{C289,290S}). The empty vector was transfected as control.

(**G**) Immunoblot analysis of GFP-p62-immunoprecipitated (IP) proteins in HEK293T cells co-expressing GFP-p62 with Flag-p62. The HEK293T cells were co-transfected with GFP-p62 and Flag-p62 (WT or the mutant p62^{C289,290S}). The empty vector was transfected as control.

(**H**) Immunoblot analysis of Flag-p62 and its mutants-immunoprecipitated (IP) proteins in HEK293T cells co-expressing GFP-p62 with Flag-p62^{D69A} or Flag-p62$^{D69A, C289,290S}$. The Flag-p62 was transfected as control.

(**I**) Immunoblot analysis of Flag-p62 and its mutants-immunoprecipitated (IP) proteins in HEK293T cells co-expressing GFP-p62 with Flag-p62 (124–440) or Flag-p62 (124–440)^{C289,290S}. The Flag-p62 was transfected as control.

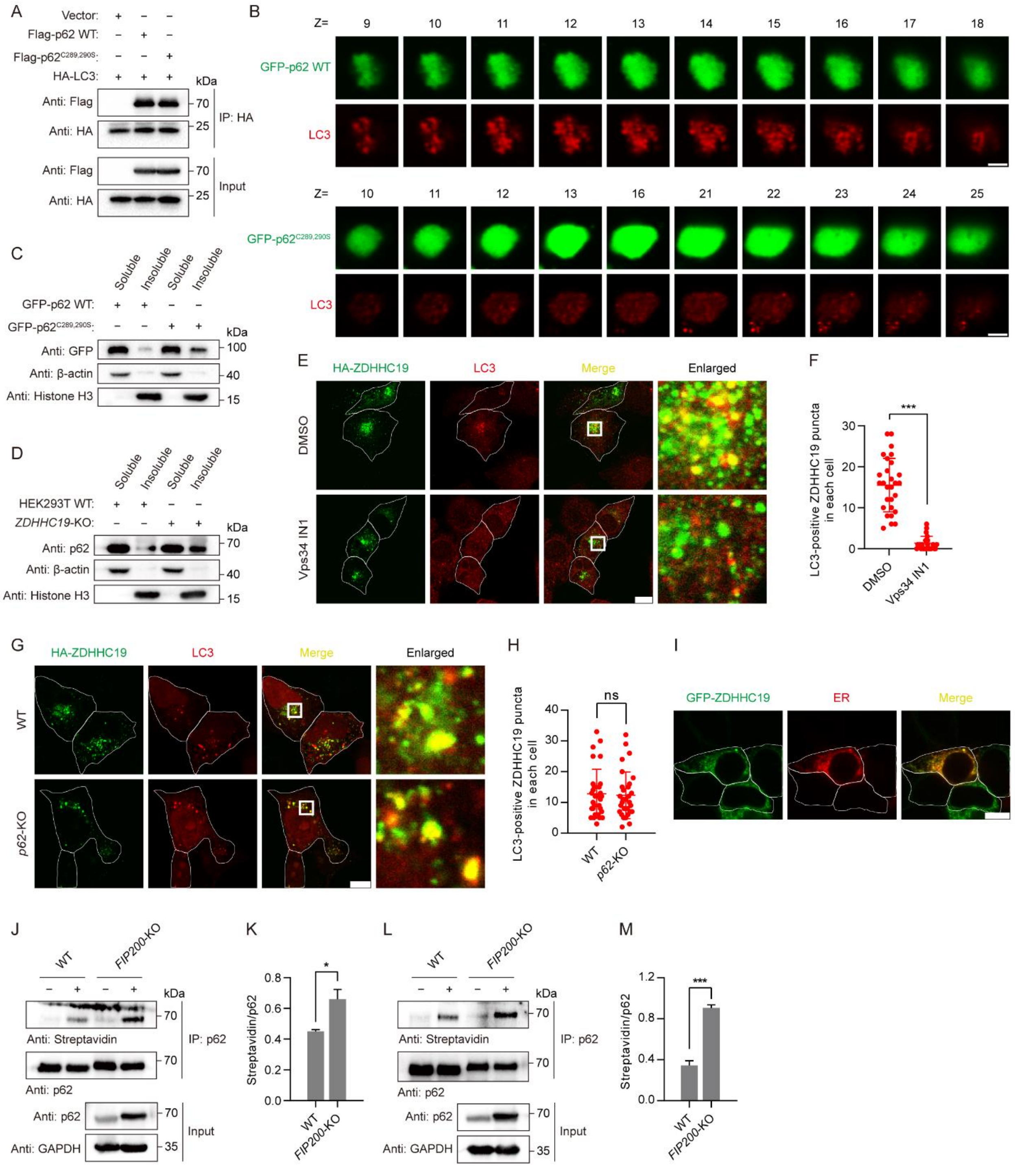

Figure S5. *S*-acylation regulates autophagic membrane localization of p62 droplets, related to Figure 5

(**A**) Immunoblot analysis of HA-LC3-immunoprecipitated (IP) proteins in HEK293T cells co-expressing HA-LC3 with Flag-p62 (WT or the mutant p62^{C289,290S}). The empty vector was transfected as control. The HA-LC3 was purified with anti-HA antibody.

(**B**) Representative images of LC3-positive p62 puncta in GFP-p62 WT or GFP-p62^{C289,290S} cells upon HA-ZDHHC19 expression. *p62*-KO NRK cells stably expressing GFP-p62 WT or GFP-p62^{C289,290S} were transiently transfected with HA-ZDHHC19. The p62- and LC3-positive puncta were analyzed by serial layer scanning. For GFP-p62 WT puncta, N=25 layers. For GFP-p62^{C289,290S} puncta, N=30 layers. Interval= 0.2 μm. Scale bar, 0.5 μm.

(**C**) Analysis of Triton X-100 soluble and insoluble fractions of GFP-p62. *p62*-KO NRK cells stably expressing GFP-p62 WT or GFP-p62^{C289,290S} were separated into Triton X-100 soluble and insoluble fractions.

(**D**) Analysis of Triton X-100 soluble and insoluble fractions of p62 in *ZDHHC19*-KO HEK293T cells.

(**E**) Representative images of the colocalization of ZDHHC19 and LC3 upon Vps34 IN1 treatment. HEK293T cells were transfected with HA-ZDHHC19, and treated with Vps34 IN1 (5 μM) for 6 h. ZDHHC19 and LC3 were immunostained with anti-HA antibody (green) and anti-LC3A/B antibody (red), respectively. Cells were visualized under confocal microscopy. Scale bar, 10 μm.

(**F**) Quantification of LC3-positive ZDHHC19 puncta in HEK293T cells as in (**E**). ***P < 0.001, Student's *t* test.

(**G**) Representative images of the colocalization of ZDHHC19 and LC3 in WT or *p62*-KO NRK cells. WT or *p62*-KO NRK cells were transfected with HA-ZDHHC19. ZDHHC19 and LC3 were immunostained with anti-HA antibody (green) and anti-LC3A/B antibody (red), respectively. Scale bar, 10 μm.

(**H**) Quantification of LC3-positive ZDHHC19 puncta in WT or *p62*-KO NRK cells as in (**G**). "ns", no significant difference, Student's *t* test.

(**I**) Representative images of the colocalization of stable ZDHHC19 and ER. The GFP-ZDHHC19 (green) stably expressing HEK293T cells were transiently transfected with DsRed2-ER (red). Scale bar, 10 μm.

(**J**) *S*-acylation of p62 in WT or *FIP200*-KO MEF cells. Endogenous p62 were immunoprecipitated with anti-p62 antibody, and *S*-acylated p62 was further detected by ABE assay.

(**K**) Quantification of p62 *S*-acylation in WT or *FIP200*-KO MEF cells as in (**J**). Data represent the mean ± SEM of three independent experiments. *$P < 0.05$, Student's *t* test.

(**L**) *S*-acylation of p62 in WT or *FIP200*-KO HeLa cells. The acylated p62 was detected by ABE assay.

(**M**) Quantification of p62 *S*-acylation in WT or *FIP200*-KO HeLa cells as in (**L**). Data represent the mean ± SEM of three independent experiments. ***$P < 0.001$, Student's *t* test.

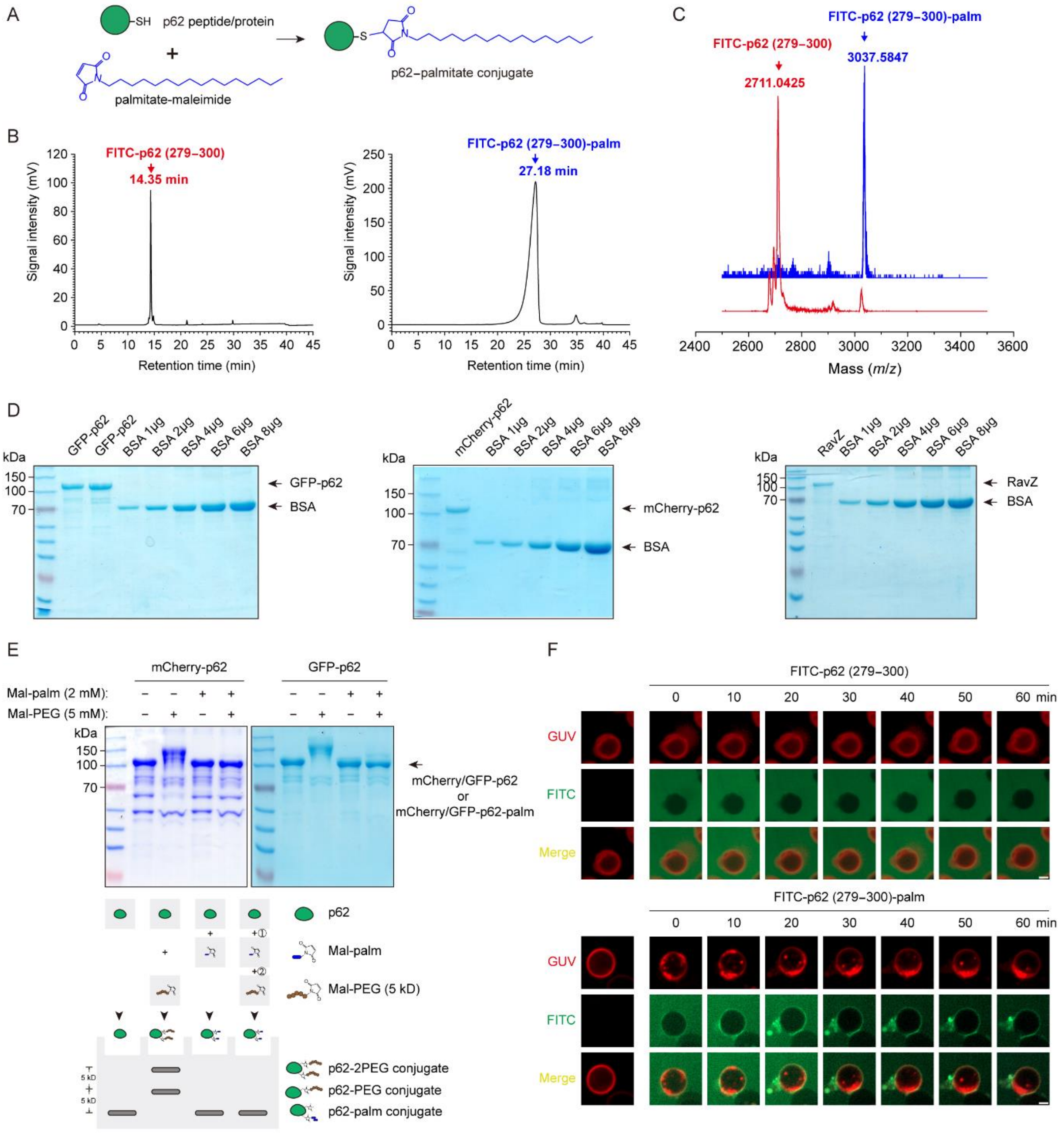

Figure S6. *S*-acylation enhances the affinity of p62 for LC3-positive membranes *in vitro*, related to Figure 6

(**A**) Schematic diagram of peptide/protein-palmitate conjugate. The palmitate-maleimide was conjugated to purified peptide or protein through the free thiol groups (–SH) of peptide or protein.

(**B**) HPLC analysis of FITC-p62 (279–300) peptide (left panel) and palmitate-maleimide conjugated FITC-p62 (279–300) peptide (right panel). The retention time of FITC-p62 (279-300) peptide is 14.35 min, and that of FITC-p62 (279–300)-palm peptide is 27.18 min. Due to its high hydrophobicity, the peptide modified with two palmitates was not purified. We used the peptide modified with one palmitate for GUV experiment. FITC-p62 (279–300)-palm, palmitate-maleimide conjugated FITC-p62 (279–300).

(**C**) Mass spectrometry analysis of FITC-p62 (279–300) and FITC-p62 (279–300)-palm. MS (MALDI–TOF): FITC-p62 (279–300), calcd. for $C_{113}H_{164}N_{29}O_{43}S_3$ $[M+H]^+$ 2711.0622, found 2711.0425; FITC-p62 (279–300)-palm, calcd. for $C_{133}H_{199}N_{30}O_{45}S_3$ $[M+H]^+$ 3037.5847, found 3037.3289.

(**D**) SDS-PAGE analysis of purified MBP-mCherry-p62 (155–440)^{C331S}, MBP-GFP^{C48S}-p62 (155–440)^{C331S} and MBP-RavZ. The arrow indicates the target proteins.

(**E**) (Upper panel) SDS-PAGE analysis of p62 and palmitate-maleimide conjugation reaction. MBP-mCherry/GFP–p62 proteins were treated with 2 μM palmitate-maleimide (Mal-palm), the resulting product was further treated with 5 μM maleimide-poly (ethylene glycol) (5 kD) (Mal-PEG). (Lower panel) The Mal-PEG conjugated to p62 caused an up-shift of p62 protein (Lane 2), while palmitate-maleimide was incapable to change the p62 migration (Lane 3). Once palmitate-maleimide blocked the free –SH group, Mal-PEG didn't provide the up-shift of p62 any more (Lane 4). GFP-p62, MBP-GFP^{C48S}-p62 (155–440)^{C331S}; GFP-p62-palm, palmitate-maleimide conjugated MBP-GFP^{C48S}-p62 (155–440)^{C331S}.

(**F**) Representative images of FITC-p62 (279–300)-positive GUVs. The FITC-p62 (279–300) or FITC-p62 (279–300)-palm (green) was incubated with rhodamine labelled GUVs (red) for 0–60 min. The fluorescence was monitored by laser scanning confocal microscope and analyzed by OLYMPUS OlyVIA software. Scale bar, 4 μm.

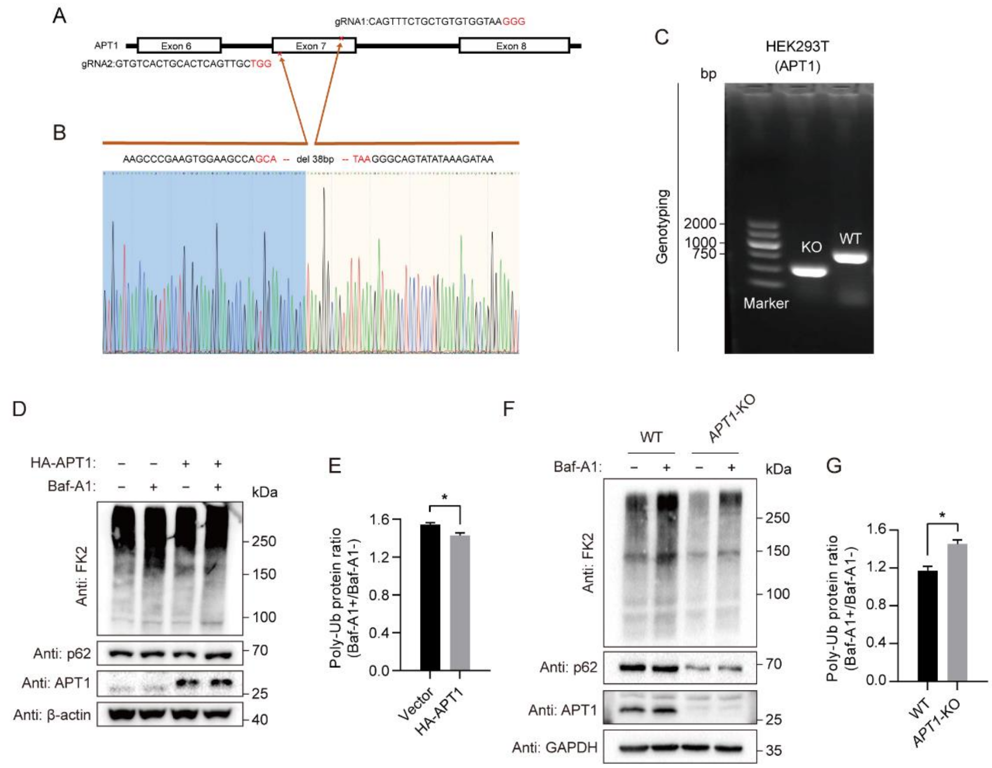

Figure S7. APT1 inhibits selective autophagic flux, related to Figure 7

(**A**) Deleting *APT1* in HEK293T cells. Targeting scheme of the truncation of exon 7 in human *APT1*. Two sgRNAs were designed to target the enzyme activity center (GTGTCACTGCACTCAGTTGC, CAGTTTCTGCTGTGTGGTAA) of human *APT1*.

(**B**) Sequencing verification of the *APT1*-KO HEK293T cell line. The knockout allele has a deletion of 38 bp on exon 7, confirmed by sequencing, and the ORF finder predicts that the knockout allele results in a premature termination of the protein.

(**C**) Electrophoresis verification of the *APT1*-KO HEK293T cell line. Genotyping was carried out by PCR amplification with corresponding primers, and the amplicons of WT and knockout alleles were 297 bp and 259 bp, respectively.

(**D**) Immunoblot analysis of total poly-ubiquitinated proteins in HEK293T cells transiently expressing HA-APT1 with or without Baf-A1 treatment for 18 h. Total proteins were extracted and detected by Western blotting.

(**E**) The ratio of the intensity of ubiquitinated proteins in Baf-A1-treated cells to that in Baf-A1-untreated cells as in (**D**). Data represent the mean ± SEM of three independent experiments. *$P < 0.05$, Student's t test.

(**F**) Immunoblot analysis of total poly-ubiquitinated proteins in WT or *APT1*-KO HEK293T cells with or without Baf-A1 treatment for 18 h.

(**G**) The ratio of the intensity of ubiquitinated proteins in Baf-A1-treated cells to that in Baf-A1-untreated cells as in (**F**). Data represent the mean ± SEM of three independent experiments. *P < 0.05, Student's t test.
