# Supplementary material for: S-acylation of p62 promotes p62 droplet recruitment into autophagosomes in mammalian autophagy
Source: Mol Cell. 2023 Oct 5;83(19):3485–3501.e11. doi: 10.1016/j.molcel.2023.09.004 (PMC10552648; doi:10.1016/j.molcel.2023.09.004)
Supplement: Document S2. Article plus supplemental information [file mmc6.pdf]

Molecular Cell

Article

S-acylation of p62 promotes p62 droplet recruitment into autophagosomes in mammalian autophagy

Graphical abstract

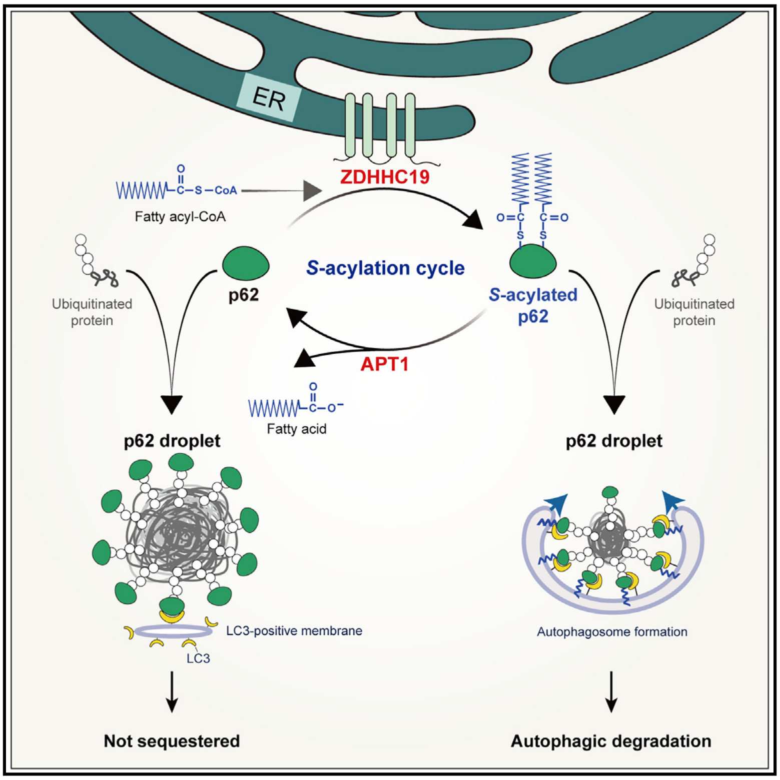

Highlights

- *S*-acyltransferase ZDHHC19 catalyzes p62 *S*-acylation at Cys289 and Cys290
- *S*-acylation facilitates autophagic degradation of p62 and ubiquitinated proteins
- *S*-acylation promotes autophagic membrane localization of p62 droplets
- APT1 deacylates p62 and suppresses p62-mediated selective autophagy

Authors

Xue Huang, Jia Yao, Lu Liu, ..., Eryan Kong, Edward W. Tate, Aimin Yang

Correspondence

aimin.yang@cqu.edu.cn

In brief

How autophagosomes efficiently sequester phase-separated p62 droplets remains unclear. Huang et al. found a posttranslational modification of p62 with long-chain fatty acids. p62 *S*-acylation regulates p62 droplet recruitment to the autophagic membrane and selective autophagic flux, thereby contributing to the control of selective autophagic clearance of ubiquitinated proteins.

Huang et al., 2023, Molecular Cell *83*, 3485–3501
October 5, 2023 © 2023 The Authors. Published by Elsevier Inc.
https://doi.org/10.1016/j.molcel.2023.09.004

CellPress

Molecular Cell

CellPress
OPEN ACCESS

Article

S-acylation of p62 promotes p62 droplet recruitment into autophagosomes in mammalian autophagy

Xue Huang,[1] Jia Yao,[1] Lu Liu,[1] Jing Chen,[1] Ligang Mei,[1] Jingjing Huangfu,[2] Dong Luo,[3] Xinyi Wang,[4,5] Changhai Lin,[1,6] Xiaorong Chen,[1] Yi Yang,[1] Sheng Ouyang,[1] Fujing Wei,[1] Zhuolin Wang,[1] Shaolin Zhang,[3] Tingxiu Xiang,[6] Dante Neculai,[4] Qiming Sun,[4,5] Eryan Kong,[2] Edward W. Tate,[7] and Aimin Yang[1,8,*]

[1]School of Life Sciences, Chongqing University, Chongqing 401331, China
[2]Institute of Psychiatry and Neuroscience, Xinxiang Key Laboratory of Protein Palmitoylation and Major Human Diseases, Xinxiang Medical University, Xinxiang, China
[3]School of Pharmacy, Chongqing University, Chongqing 401331, China
[4]International Institutes of Medicine, The Fourth Affiliated Hospital of Zhejiang University School of Medicine, Yiwu, Zhejiang, China
[5]Department of Biochemistry and Department of Cardiology of Second Affiliated Hospital, Zhejiang University School of Medicine, Hangzhou 310058, China
[6]Chongqing Key Laboratory of Translational Research for Cancer Metastasis and Individualized Treatment, Chongqing University Cancer Hospital, Chongqing 400030, China
[7]Department of Chemistry, Imperial College London, 82 Wood Lane, London W12 0BZ, UK
[8]Lead contact
*Correspondence: aimin.yang@cqu.edu.cn

https://doi.org/10.1016/j.molcel.2023.09.004

SUMMARY

p62 is a well-characterized autophagy receptor that recognizes and sequesters specific cargoes into autophagosomes for degradation. p62 promotes the assembly and removal of ubiquitinated proteins by forming p62-liquid droplets. However, it remains unclear how autophagosomes efficiently sequester p62 droplets. Herein, we report that p62 undergoes reversible *S*-acylation in multiple human-, rat-, and mouse-derived cell lines, catalyzed by zinc-finger Asp-His-His-Cys *S*-acyltransferase 19 (ZDHHC19) and deacylated by acyl protein thioesterase 1 (APT1). *S*-acylation of p62 enhances the affinity of p62 for microtubule-associated protein 1 light chain 3 (LC3)-positive membranes and promotes autophagic membrane localization of p62 droplets, thereby leading to the production of small LC3-positive p62 droplets and efficient autophagic degradation of p62-cargo complexes. Specifically, increasing p62 acylation by upregulating ZDHHC19 or by genetic knockout of APT1 accelerates p62 degradation and p62-mediated autophagic clearance of ubiquitinated proteins. Thus, the protein *S*-acylation-deacylation cycle regulates p62 droplet recruitment to the autophagic membrane and selective autophagic flux, thereby contributing to the control of selective autophagic clearance of ubiquitinated proteins.

INTRODUCTION

The efficient clearance of damaged proteins and protein aggregates is mediated by two major quality control pathways, the ubiquitin-proteasome system (UPS) and macroautophagy (hereafter referred to as autophagy), which are crucial for maintenance of cellular homeostasis.[1–3] The UPS mostly degrades ubiquitinated misfolded proteins through proteasomes, whereas autophagy mediates the clearance of cellular ubiquitinated aggregates.[4,5] In particular, when proteasome activity declines during aging or under environmental stresses, selective autophagic clearance of ubiquitinated proteins is a compensatory mechanism to the UPS.[6–8] Dysregulation of selective autophagic clearance of specific ubiquitinated proteins is associated with aging-related diseases, including cancer and neurodegenerative disorders.[9–11]

Selective autophagy is mediated via autophagy receptors that recognize and sequester specific cargo into the autophagosome, which subsequently fuses with a lysosome, resulting in the degradation of ubiquitinated proteins by lysosomal hydrolases.[12–14] Sequestosome 1 (SQSTM1)/p62 (hereafter referred to as p62) is an autophagy receptor that participates in autophagic clearance of ubiquitinated proteins, whereupon it is itself degraded along with its cargoes.[15–17] p62 possesses multiple domains, including a Phox1 and Bem1p (PB1) domain, a microtubule-associated protein 1 light chain 3 (LC3)-interacting region (LIR) and a ubiquitin-associated (UBA) domain.[17,18] In selective autophagy, p62 binds to ubiquitinated proteins via its UBA domain and interacts with

This is an open access article under the CC BY license (http://creativecommons.org/licenses/by/4.0/).

LC3 on autophagosomes via its LIR domain, ultimately leading to ubiquitinated protein degradation through fusion with lysosomes.[19–23] Therefore, p62 is a vital player in the selective autophagic clearance of ubiquitinated proteins.

In cells, p62 promotes the assembly and removal of ubiquitinated proteins by forming insoluble cytoplasmic inclusions known as p62 bodies.[21] p62 bodies have liquid-like properties formed by polyubiquitin chain-triggered liquid-liquid phase separation,[24,25] which is required for p62 body formation and p62-mediated selective autophagy.[26,27] Several p62-binding partners and posttranslational modifications of p62 have been shown to alter the liquid-liquid phase separation behavior of p62 droplets, thereby regulating p62-mediated selective autophagy.[6,28–33] Although the interaction of membrane-associated LC3 with the LIR of p62 is required for p62-mediated selective clearance of ubiquitinated proteins,[22] it remains unclear how autophagosomes efficiently sequester phase-separated p62 droplets. As p62 condensates are unique and important structures in regulating selective autophagy, it is of critical importance to understand molecular mechanisms underlying p62 condensate recruitment to autophagic membranes.

Protein *S*-acylation is the covalent attachment of a long-chain fatty acid to specific cysteine residues within a protein; often, this fatty acid is palmitate or stearate.[34,35] *S*-acylation significantly increases the hydrophobicity of proteins and contributes to their membrane association, thereby playing critical roles in protein function and cell signaling.[36–38] *S*-acylation is readily reversible due to the lability of the thioester bond between the fatty acyl group and cysteine residue, therefore leading to spatial-temporal control of protein function and subcellular localization.[39–41] In the present study, we report that p62 undergoes reversible *S*-acylation, which is mediated by the zinc-finger Asp-His-His-Cys *S*-acyltransferase 19 (ZDHHC19) and the acyl protein thioesterase 1 (APT1). Mechanistically, *S*-acylation enhances the affinity of p62 for LC3-positive membranes and promotes autophagic membrane localization of p62 droplets, leading to the production of small LC3-positive p62 droplets, and the subsequent degradation of p62 and ubiquitinated substrates through autophagy. Our results reveal an important molecular mechanism underlying p62 droplet recruitment to the autophagic membrane.

RESULTS

p62 is *S*-acylated in mammalian cells

To measure whether p62 is an acylated protein, we performed an acyl-biotin exchange (ABE) assay, which selectively replaces thioester-linked acyl groups with biotin that can be detected by western blotting probed with streptavidin-horseradish peroxidase (HRP) (Figure S1A).[42] We observed the apparent acylation of endogenous p62 in human cervical cancer HeLa cells and normal rat kidney (NRK) cells (Figure 1A). In addition, we also found that exogenously expressed p62 was acylated in breast cancer MCF7 cells (Figures S1B and S1C), consistent with other proteomic studies.[43,44] We performed further studies using chemical reporters of protein *S*-acylation (Figures S1D and S1E),[45] and we confirmed that p62 is *S*-acylated through thioester bonds that are cleavable by treatment with hydroxylamine (HAM [NH_2OH]) (Figure 1B). Specifically, p62 was efficiently labeled by an alkynyl stearic acid (Alk-C18) probe but not significantly labeled by a probe with a chain length of C16 (Alk-C16) (Figure 1B). We then examined p62 *S*-acylation by ABE assay in cells under autophagy induction (starvation or treatment with the mammalian target of rapamycin [mTOR] inhibitor Torin 1), proteasomal inhibition (treatment with the proteasome inhibitor MG132), or translation inhibition (treatment with the translation inhibitor puromycin). We found that proteasomal inhibition increased the *S*-acylation of p62 (Figure 1C). Thus, p62 undergoes *S*-acylation in mammalian cells.

Cys289 and Cys290 of p62 are *S*-acylated

We next determined the acylation sites of p62. The human p62 protein contains 14 cysteine residues of which six cysteine residues were predicted to be acylated by GPS- and CSS-palm software (Figure S1F).[46,47] To verify modification sites experimentally, we individually mutated each predicted cysteine to serine and examined the acylation levels of the mutants by ABE assay. Compared with p62 wild type (WT), the mutants $p62^{C289S}$ and $p62^{C290S}$ showed markedly reduced acylation levels, and the double-Cys mutant $p62^{C289,290S}$ showed almost undetectable acylation (Figures 1D and S1G), suggesting that Cys^{289} and Cys^{290} are *S*-acylated.

To further define the region of p62 required for acylation, we constructed fusion motifs consisting of FLAG-GFP and peptides derived from p62, including p62 (285–294), p62 (283–296), and p62 (279–300), and we then examined the acylation levels of these fusions by ABE assay (Figure 1E). We observed that p62 (279–300), but not the double-Cys mutant, was acylated (Figure 1F), suggesting that the p62 (279–300) region is required for p62 *S*-acylation. Moreover, the acylation sites, Cys^{289} and Cys^{290}, are conserved among species and are located in an intrinsically disordered region of p62 ranging from 264 to 390 that has been shown to be critical for p62-mediated selective autophagic clearance of ubiquitinated proteins (Figure 1G).[21,48]

Therefore, these results consistently suggest that Cys^{289} and Cys^{290} are the acylation sites of p62.

S-acyltransferase ZDHHC19 catalyzes p62 *S*-acylation

Protein *S*-acylation is catalyzed by a family of ZDHHC domain-containing *S*-acyltransferases.[49,50] These enzymes are ubiquitous in eukaryotes, with 23 distinct ZDHHC-encoding genes in the human genome.[51–53] To identify which *S*-acyltransferases were responsible for p62 *S*-acylation, each of the 23 hemagglutinin (HA)-tagged ZDHHC enzymes was expressed in cells, and the acylation level of endogenous p62 was subsequently analyzed by ABE assay. We observed an increase in endogenous p62 acylation levels following the expression of ZDHHC4, 5, 7, 19, or 24, with ZDHHC19 being the most efficient (Figures S2A–S2C). ZDHHC19 also showed the clearest co-localization with p62 (Figures 2A, S2D, and S2E). To test the interaction between ZDHHC19 and p62, we performed a co-immunoprecipitation (coIP) assay of HA-ZDHHC19 and FLAG-p62 in HEK293T and NRK cells. We observed a physical interaction between ZDHHC19 and p62 (Figures 2B, 2C, and S2F). Furthermore, we performed TurboID-mediated proximity labeling of ZDHHC19 (Figure 2D)[54] and identified endogenous p62

CellPress
OPEN ACCESS

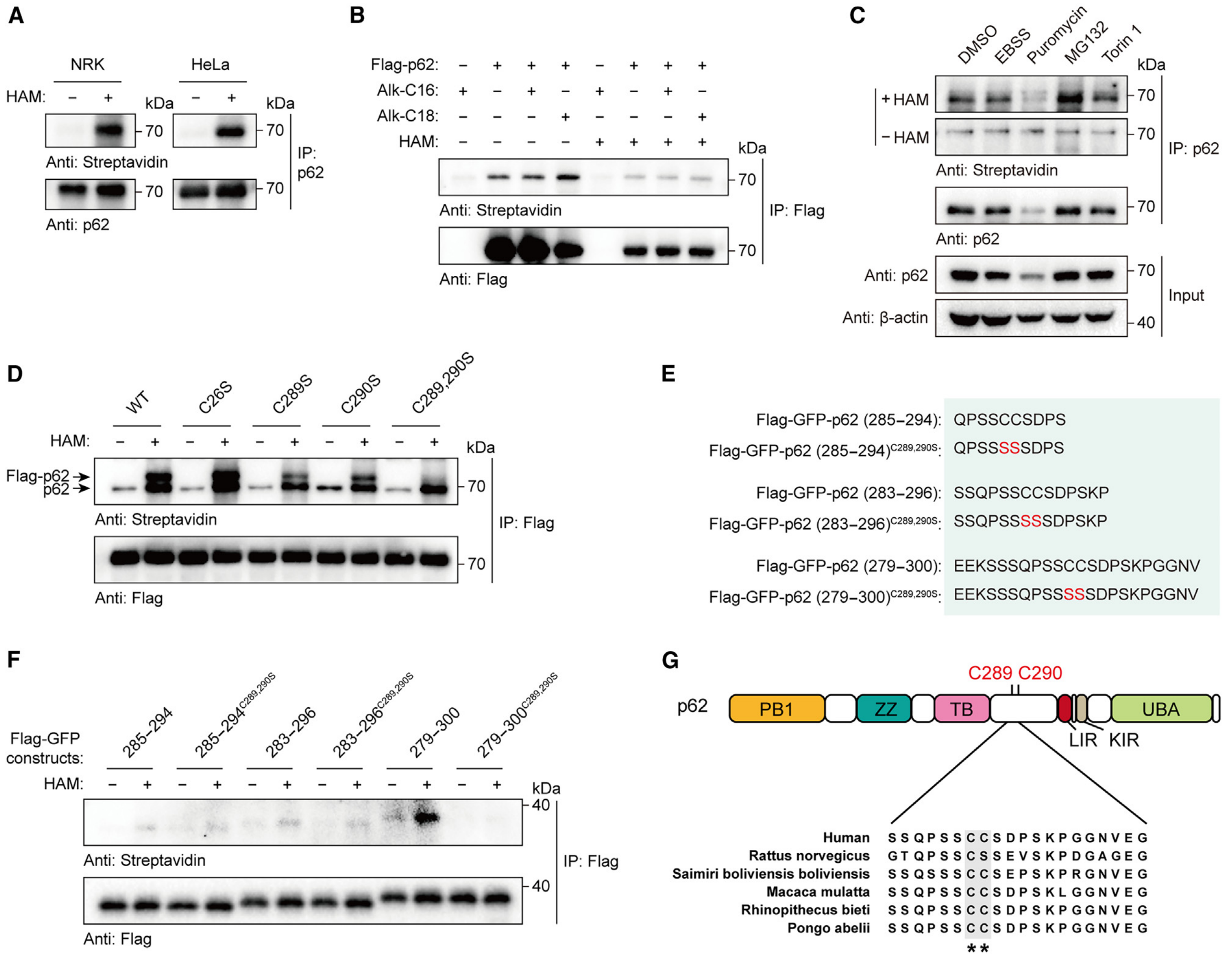

Figure 1. p62 undergoes *S*-acylation at Cys289 and Cys290

(A) *S*-acylation of endogenous p62 detected by ABE assay in NRK and HeLa cells. Endogenous p62 was immunoprecipitated with anti-p62 antibody. HAM, hydroxylamine.

(B) *S*-acylation of FLAG-p62 detected by chemical reporters in HEK293T cells. The cells transiently expressing FLAG-p62 were metabolically labeled with Alk-C16 or Alk-C18 (100 μM) for 12 h. FLAG-p62 was immunoprecipitated with anti-FLAG antibody. Alk-C16, palmitic acid alkyne; Alk-C18, stearic acid alkyne.

(C) *S*-acylation of p62 detected by ABE assay in HEK293T cells incubated in starvation medium (EBSS) and treated with puromycin, MG132, or Torin 1.

(D) *S*-acylation of FLAG-p62 mutants detected by ABE assay. Because of p62 oligomerization, anti-FLAG antibody precipitated exogenous FLAG-p62 (the upper band) as well as p62 (the lower band).

(E) Scheme of the fusion motifs consisting of FLAG-GFP and peptides derived from the p62 sequence. Three peptides, including p62 (285–294), p62 (283–296), and p62 (279–300), were selected to define the region of p62 required for *S*-acylation. The peptides containing the mutation C289,290S were designed as controls.

(F) *S*-acylation of FLAG-GFP fusions detected by ABE assay in (E).

(G) Conservation analysis of p62 *S*-acylation sites Cys289 and Cys290 among various species. PB1, Phox1, and Bem1p domain; ZZ, ZZ-type zinc-finger domain; TB, TRAF6-binding domain; LIR, LC3-interacting region; KIR, Keap1-interacting region; UBA, ubiquitin-associated domain.

See also Figure S1.

as a ZDHHC19 interactor in living cells (Figure 2E). To confirm that ZDHHC19 is the endogenous *S*-acyltransferase mediating p62 modification, we generated *ZDHHC19*-knockout (KO) HEK293T cells using the CRISPR-Cas9 system (Figures S2G–S2I). We observed that *S*-acylation of p62 in *ZDHHC19*-KO cells was substantially reduced compared with that in control cells (Figures 2F and 2G). Taken together, these results suggest that ZDHHC19 catalyzes p62 *S*-acylation.

S-acylation facilitates the degradation of p62

As an autophagic receptor, p62 is itself degraded along with its cargoes by autophagy. We therefore investigated whether p62 *S*-acylation is involved in p62 turnover. To achieve this purpose, we expressed plasmids encoding p62 WT or the acylation-deficient mutant p62^{C289,290S} in WT or *p62*-KO NRK cells. We found that in both cell lines, a substantial amount of the autophagy marker phosphatidylethanolamine (PE)-modified LC3 (LC3-II)[55]

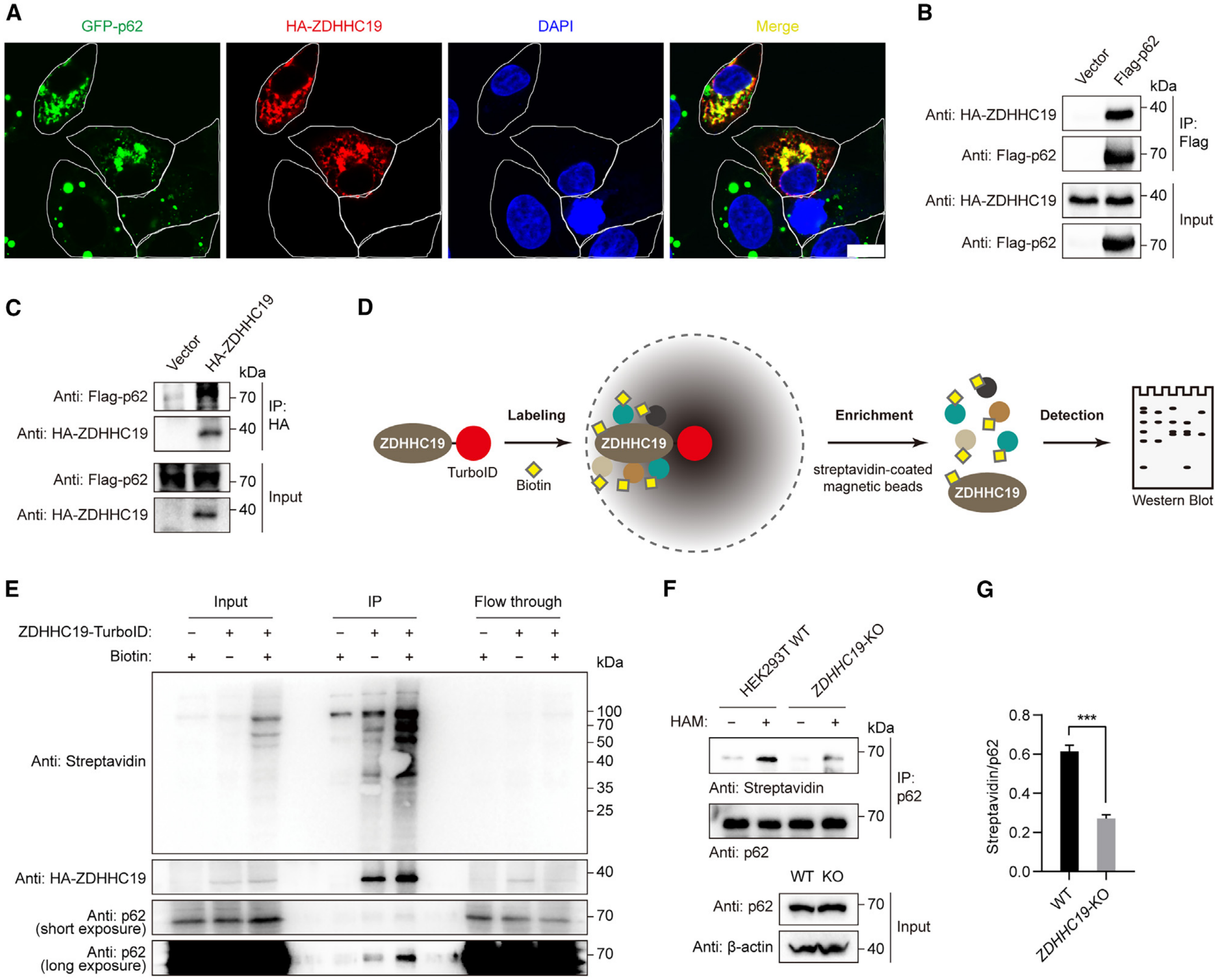

Figure 2. ZDHHC19 mediates p62 *S*-acylation

(A) Representative images of HA-ZDHHC19 localization in *p62*-KO NRK cells stably expressing GFP-p62 (green). HA-ZDHHC19 was transfected and immunostained with anti-HA antibody (red). The nucleus was visualized by DAPI (blue). Scale bars, 10 μm.

(B) Immunoblot analysis of FLAG-p62-immunoprecipitated (IP) proteins in HEK293T cells co-expressing FLAG-p62 with HA-ZDHHC19.

(C) Immunoblot analysis of HA-ZDHHC19-IP proteins in NRK cells co-expressing FLAG-p62 with HA-ZDHHC19.

(D) Scheme of TurboID-mediated proximity labeling of ZDHHC19. Cells were transfected with the plasmid encoding ZDHHC19-TurboID and labeled with D-biotin.

(E) Immunoblot analysis of ZDHHC19-TurboID-labeled proteins. Total proteins were precipitated from cell lysates and enriched with streptavidin-coated magnetic beads. Biotinylated proteins were eluted and further analyzed by western blotting with HRP-conjugated streptavidin. Biotinylated p62 was analyzed by western blotting with anti-p62 antibody.

(F) *S*-acylation level of p62 detected by ABE assay in *ZDHHC19*-KO HEK293T cells.

(G) Quantification of p62 *S*-acylation levels as in (F). Data represent the mean ± SEM of three independent experiments. ***$p < 0.001$, Student's t test.

See also Figure S2.

was accumulated upon induction of autophagy with the mTOR inhibitor Torin 1 while degradation of p62 WT was faster than that of p62^{C289,290S}, which was reversed by the lysosome V-ATPase inhibitor bafilomycin A1 (Baf-A1) (Figures 3A, 3B, and S3A–S3D).

We next examined the effect of increasing p62 acylation on p62 degradation by upregulating ZDHHC19 expression. We observed that the expression of ZDHHC19, but not other ZDHHC enzymes, facilitated p62 degradation under basal conditions or upon autophagy induction (Figures S3E and S3F). In contrast, expression of ZDHHC19^{C142S}, a catalytically inactive ZDHHC19 mutant, did not affect p62 degradation (Figures 3C and 3D). We further observed a delay in p62 degradation in *ZDHHC19*-KO cells compared with control cells, which was rescued by re-expressing ZDHHC19 (Figures 3E and 3F). To exclude the possibility that such changes of p62 level were due to lysosomal activity, we evaluated lysosome function upon ZDHHC19 deficiency or overexpression. We found no

CellPress
OPEN ACCESS

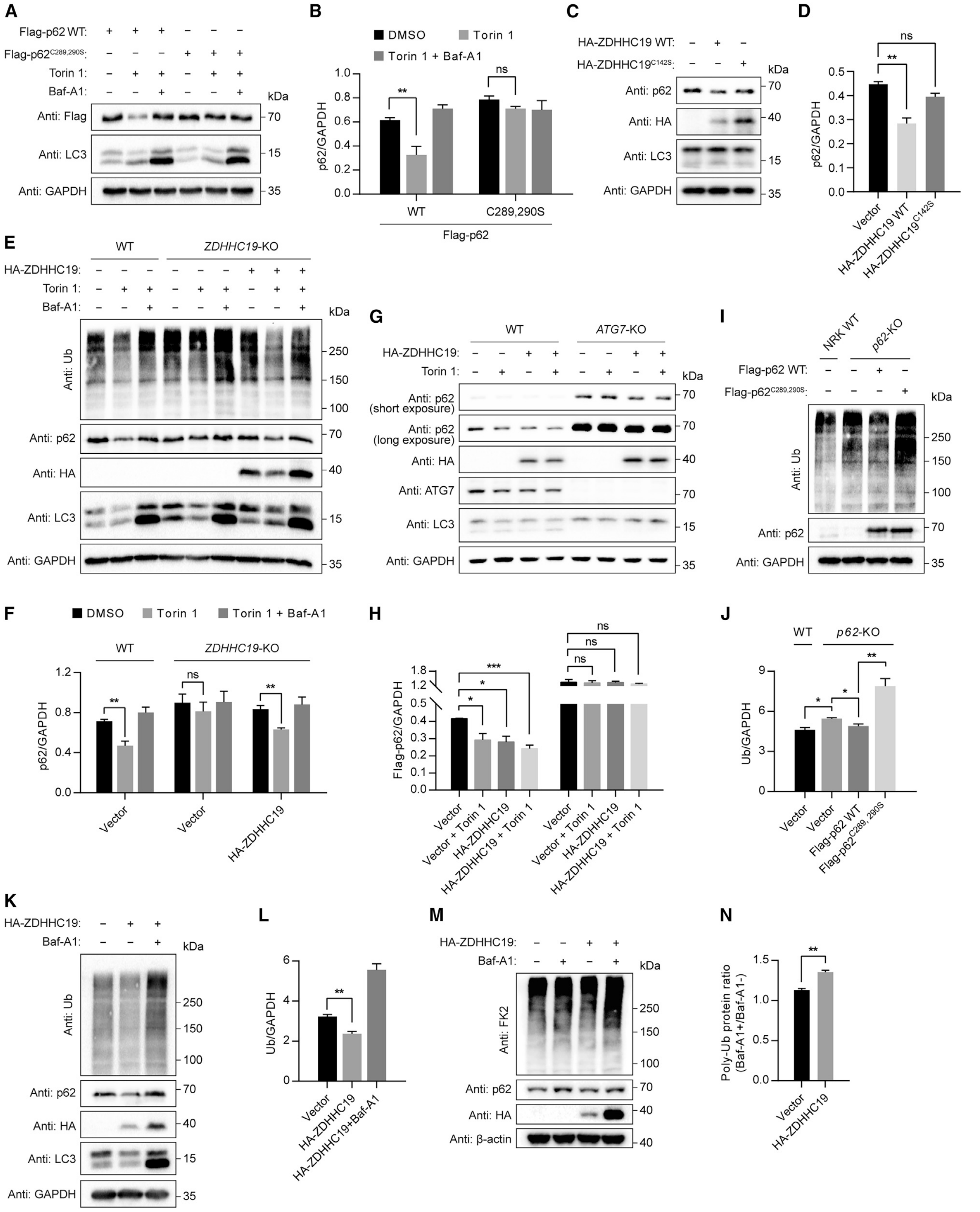

(legend on next page)

change in lysosomal pH in *ZDHHC19*-KO cells and cells transiently expressing ZDHHC19, compared with control cells (Figures S3G and S3H). In addition, ZDHHC19 expression did not change p62 protein levels in autophagy-deficient *ATG7*-KO HEK293T cells, suggesting that ZDHHC19-mediated p62 degradation is dependent on the autophagy pathway (Figures 3G and 3H).

Thus, ZDHHC19-mediated p62 *S*-acylation facilitates autophagic degradation of p62.

S-acylation promotes p62-mediated selective autophagic flux

p62 mediates the delivery of ubiquitinated substrates into autophagosomes, and the latter fuse directly with lysosomes for degradation.[56] We observed that *p62*-KO led to accumulation of ubiquitinated proteins, which was fully rescued by re-expressing p62 (Figures 3I and 3J), consistent with previous studies.[57,58] However, cells re-expressing the *S*-acylation-deficient mutant p62^{C289,290S} retained the clearance defect for ubiquitinated proteins, suggesting that p62 *S*-acylation is required for p62-mediated selective autophagic clearance of ubiquitinated proteins. In addition, expression of ZDHHC19 facilitated degradation of ubiquitinated proteins, which was blocked by Baf-A1 (Figures 3K, 3L, and S3I), while *p62* KO inhibited ZDHHC19-induced degradation of ubiquitinated proteins (Figures S3J and S3K), suggesting that ZDHHC19-mediated degradation of ubiquitinated proteins is dependent on p62. We also observed a delay in the clearance of ubiquitinated proteins in *ZDHHC19*-KO cells compared with control cells, which was rescued by re-expressing ZDHHC19 (Figures 3E and S3L).

To further investigate the effect of p62 *S*-acylation on selective autophagic flux, we treated *p62*-KO NRK cells stably expressing p62 WT or p62^{C289,290S} with Baf-A1 and then assessed the accumulation of ubiquitinated proteins. We observed that lysosomal inhibition caused greater accumulation of ubiquitinated proteins in p62 WT cells than in p62^{C289,290S} cells (Figures S3M and S3N). Furthermore, lysosomal inhibition led to the accumulation of poly-ubiquitinated proteins upon ZDHHC19 expression (Figures 3M and 3N), suggesting that ZDHHC19-mediated p62 *S*-acylation promotes p62-mediated selective autophagic flux, thereby facilitating the entry of ubiquitinated substrates into autophagosomes.

To determine the physiological function of p62 *S*-acylation, we compared the viability of these cell lines. We observed that p62^{C289,290S} NRK cells and *ZDHHC19*-KO HEK293T cells showed reduced viability compared with p62 WT NRK cells and HEK293T cells, respectively, upon proteasomal inhibition (Figures S3O–S3Q), suggesting an important physiological role for p62 *S*-acylation upon induction of proteotoxic stress.

Taken together, these results suggest that ZDHHC19-catalyzed p62 *S*-acylation facilitates p62-mediated selective autophagy, which indicates an important role for p62 *S*-acylation in cell survival under stress.

S-acylation promotes the formation of small p62 droplets

Next, we investigated why p62 *S*-acylation facilitates degradation of p62 and ubiquitinated proteins through autophagy. p62 tends to form cytoplasmic aggregates with ubiquitinated proteins and is often observed in discrete punctate structures known as cellular p62 bodies in cells.[24,25] We therefore tested the effect of *S*-acylation on the formation of p62 puncta. Surprisingly, we observed, under similar levels of GFP-p62 WT and GFP-p62^{C289,290S}, a significant accumulation of p62 puncta with a diameter greater than 1 μm (large p62 puncta)[59] in GFP-p62^{C289,290S}-expressing cells compared with GFP-p62 WT-expressing cells (Figures 4A–4C), suggesting that deficiency in p62 *S*-acylation results in the formation of large p62 puncta. This finding was confirmed in *ZDHHC19*-KO cells, where we also observed large p62 puncta (Figures 4D and 4E). Also, the expression of ZDHHC19, but not the catalytically inactive mutant ZDHHC19^{C142S}, enabled the formation of smaller p62 puncta compared with that of the control (Figures 4F and 4G). Furthermore, transient expression of ZDHHC19 did not distribute p62 bodies into small puncta in GFP-p62^{C289,290S}-expressing NRK cells, suggesting that the formation of small p62 droplets

Figure 3. *S*-acylation facilitates the degradation of p62 and ubiquitinated proteins

(A) Immunoblot analysis of FLAG-p62 WT and FLAG-p62^{C289,290S} proteins in *p62*-KO NRK cells. Cells were transiently transfected with FLAG-p62 WT or FLAG-p62^{C289,290S} and treated with Torin 1 (1 μM) for 1 h, with or without Baf-A1 (1 μM).

(B) Quantification of FLAG-p62 WT and FLAG-p62^{C289,290S} protein levels as in (A).

(C) Immunoblot analysis of p62 protein upon transiently expressing ZDHHC19 WT or ZDHHC19^{C142S} in NRK cells.

(D) Quantification of p62 protein levels as in (C).

(E) Immunoblot analysis of p62 protein and ubiquitinated proteins in WT or *ZDHHC19*-KO HEK293T cells. Cells were transfected with empty vector or HA-ZDHHC19 and then incubated with Torin 1 (1 μM) for 4 h, with or without Baf-A1 (1 μM).

(F) Quantification of p62 protein levels as in (E).

(G) Immunoblot analysis of p62 protein in *ATG7*-KO cells upon HA-ZDHHC19 expression. HEK293T WT or *ATG7*-KO cells were transfected with empty vector or HA-ZDHHC19 and then treated with Torin 1 (1 μM) for 1 h.

(H) Quantification of p62 protein levels as in (G).

(I) Immunoblot analysis of total ubiquitinated proteins in WT or *p62*-KO NRK cells transiently expressing FLAG-p62 WT or FLAG-p62^{C289,290S}.

(J) Quantification of total ubiquitinated proteins as in (I).

(K) Immunoblot analysis of p62 and total ubiquitinated proteins in NRK cells transiently expressing ZDHHC19.

(L) Quantification of total ubiquitinated protein levels as in (K).

(M) Immunoblot analysis of p62 protein and total ubiquitinated proteins upon ZDHHC19 expression in NRK cells.

(N) Quantification of total ubiquitinated protein levels as in (M).

Data in (B), (D), (F), (H), (J), (L), and (N) represent the mean ± SEM of three independent experiments. ns, no significant difference; *$p < 0.05$; **$p < 0.01$; ***$p < 0.001$; Student's t test.

See also Figure S3.

CellPress
OPEN ACCESS

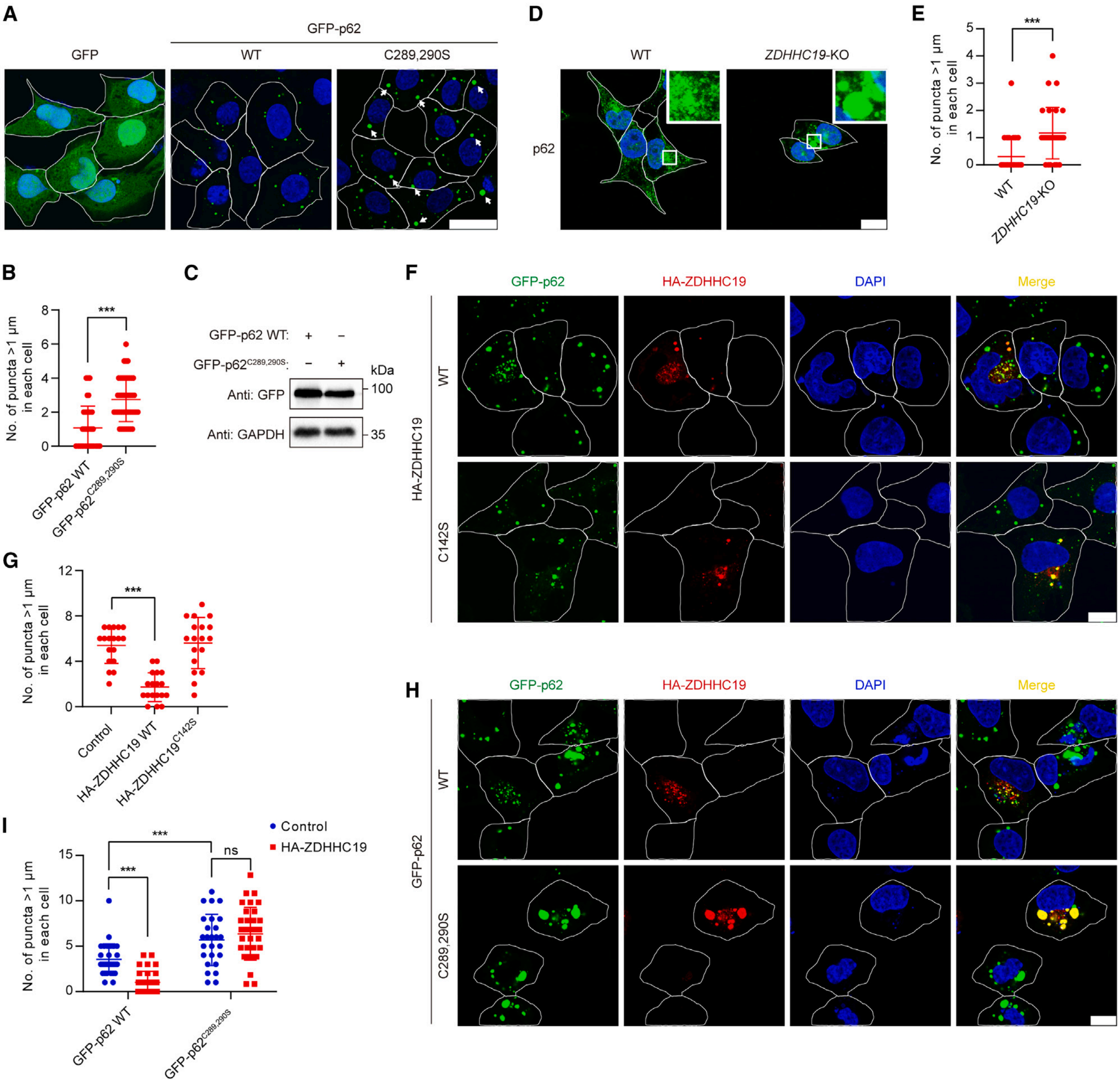

Figure 4. *S*-acylation promotes the formation of small LC3-positive p62 puncta

(A) Representative images of p62 puncta in GFP-p62 WT or GFP-p62^{C289,290S} cells. GFP-p62 WT or GFP-p62^{C289,290S} was stably expressed in *p62*-KO NRK cells. Cells were fixed with paraformaldehyde and observed under confocal microscopy. The arrows indicate p62 puncta with a diameter of more than 1 μm. Scale bars, 10 μm.

(B) Quantification of the number of p62 puncta with a diameter of more than 1 μm in each cell as in (A). ***p < 0.001, Student's t test.

(C) Immunoblot analysis of p62 proteins in GFP-p62 WT or GFP-p62^{C289,290S} cells.

(D) Representative images of p62 puncta in *ZDHHC19*-KO HEK293T cells. Scale bars, 10 μm.

(E) Quantification of the number of p62 puncta with a diameter of more than 1 μm in each cell as in (D). ***p < 0.001, Student's t test.

(F) Representative images of p62 puncta in GFP-p62 NRK cells transiently expressing HA-ZDHHC19 WT or HA-ZDHHC19^{C142S}. Scale bars, 10 μm.

(G) Quantification of the number of p62 puncta with a diameter of more than 1 μm in each cell as in (F). ***p < 0.001, Student's t test.

(H) Representative images of p62 puncta in GFP-p62 WT or GFP-p62^{C289,290S} NRK cells transiently expressing HA-ZDHHC19. Scale bars, 10 μm.

(I) Quantification of the number of p62 puncta with a diameter of more than 1 μm in each cell as in (H). ns, no significant difference. ***p < 0.001, Student's t test.

See also Figure S4.

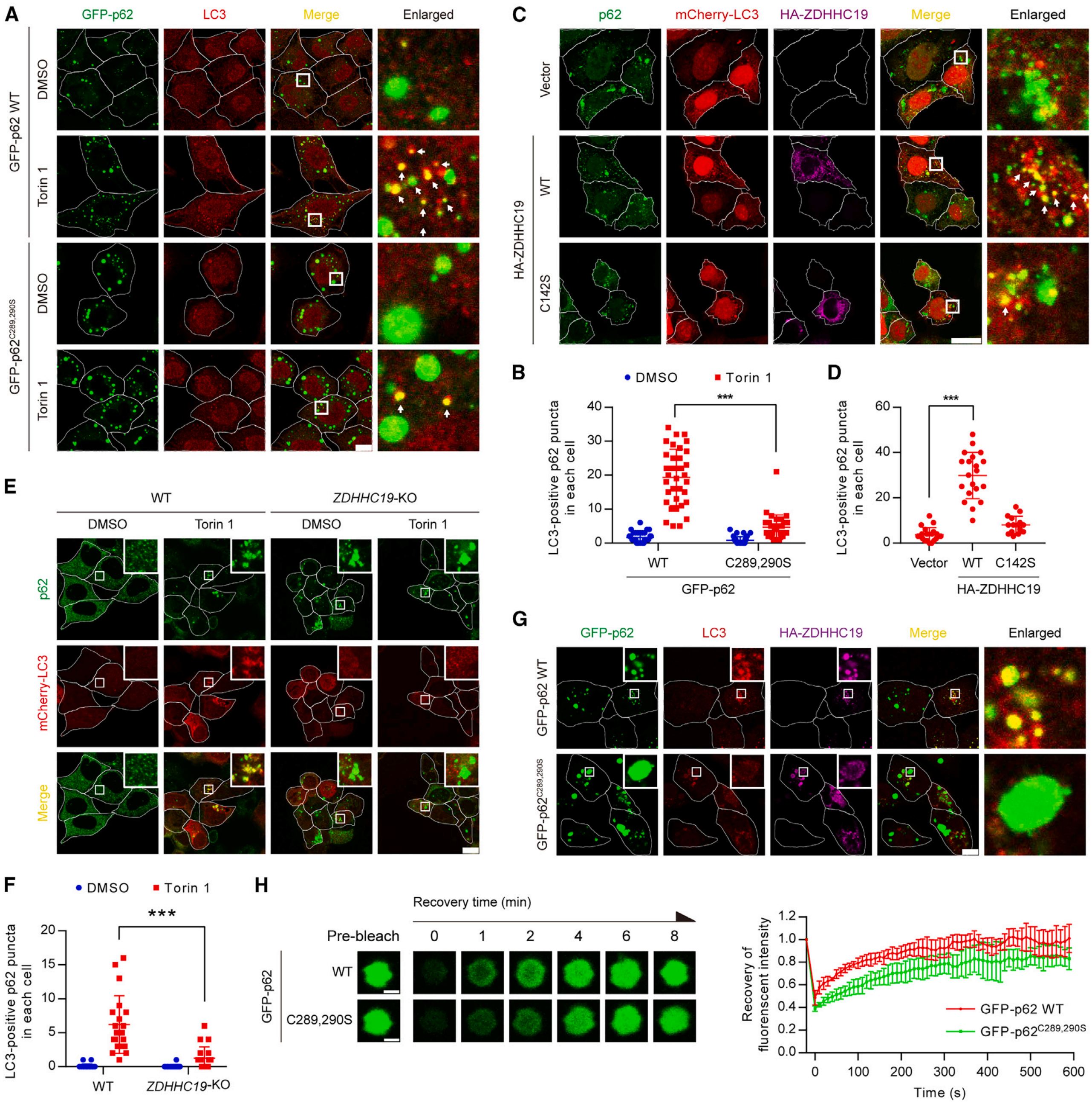

Figure 5. *S*-acylation regulates autophagic membrane localization of p62 droplets

(A) Representative images of autophagosome formation in GFP-p62 WT or GFP-p62^{C289,290S} cells upon autophagy induction. *p62*-KO NRK cells stably expressing GFP-p62 WT or GFP-p62^{C289,290S} (green) were incubated with Torin 1 (1 μM) for 3 h, followed by immunostaining with anti-LC3A/B antibody (red). Scale bars, 10 μm.

(B) Quantification of LC3-positive p62 puncta in GFP-p62 WT or GFP-p62^{C289,290S} cells upon autophagy induction as in (A). ***$p < 0.001$, Student's t test.

(C) Representative images of autophagosome formation in mCherry-LC3 HEK293 cells upon the expression of ZDHHC19 WT or ZDHHC19^{C142S}. HEK293 cells stably expressing mCherry-LC3 (red) were transiently expressed with HA-ZDHHC19 WT or HA-ZDHHC19^{C142S}, followed by immunostaining with anti-p62 antibody (green) and anti-HA antibody (purple). Scale bars, 10 μm.

(D) Quantification of LC3-positive p62 puncta in each cell as in (C). ***$p < 0.001$, Student's t test.

(E) Representative images of p62 puncta and autophagosome in WT or *ZDHHC19*-KO HEK293T cells. HEK293T cells transiently expressing mCherry-LC3 (red) were treated with or without Torin 1 (1 μM) for 3 h and immunostained with anti-p62 antibody (green). Scale bars, 10 μm.

(F) Quantification of LC3-positive p62 puncta in each cell as in (E). ***$p < 0.001$, Student's t test.

(legend continued on next page)

CellPress
OPEN ACCESS

induced by ZDHHC19 is dependent on *S*-acylation at Cys289 and Cys290 of p62 (Figures 4H and 4I).

Previous studies have suggested that binding affinity of p62 and ubiquitinated proteins, as well as self-oligomerization of p62, determines the size distribution of p62 droplets.[6,28,30,60] We observed that both GFP-p62 puncta and large GFP-p62^{C289,290S} puncta co-localized with ubiquitin (Figure S4A). In addition, p62 WT and p62^{C289,290S} co-precipitated equivalent amounts of ubiquitinated proteins (Figure S4B), consistent with *in vitro* pull-down data (Figure S4C). Using non-reduced SDS-PAGE, we observed the same amount of p62 monomer and oligomer in p62 WT- or p62^{C289,290S}-expressing cells (Figure S4D). We further found that the overexpression of ZDHHC19 did not affect the distribution of p62 monomer and oligomer (Figure S4E). Moreover, coIP analysis between p62 with different tags showed that *S*-acylation did not affect PB1 domain-dependent p62-p62 interaction (Figures S4F–S4I). These results suggest that p62 *S*-acylation has no effect on p62-ubiquitin binding and its oligomerization.

Therefore, p62 *S*-acylation mediated by ZDHHC19 promotes the formation of small p62 droplets, and deficiency in p62 *S*-acylation leads to the formation of large p62 droplets, which is not due to changes in p62-ubiquitin binding or oligomerization.

S-acylation regulates autophagic membrane localization of p62 droplets

We next investigated why p62 *S*-acylation promotes the formation of small p62 droplets. p62 bodies have viscous liquid-like properties.[24,61] p62 puncta are observed both as membrane-free protein aggregates or as being engulfed by autophagosomes or autolysosomes.[21] Studies have suggested that LC3-positive membranes associate with p62 droplets and deform p62 droplets through a wetting process, a phenomenon in which liquid-like properties of droplets allow for droplet deformation into spherical caps when a droplet contacts a surface.[60,62] *S*-acylation has previously been shown to regulate the membrane localization and trafficking of proteins.[36–38] We therefore investigated whether p62 *S*-acylation regulates autophagic membrane localization of p62 droplets. In GFP-p62 WT-expressing cells, p62 puncta showed co-localization with the autophagosome marker LC3 upon autophagy induction and became small p62 puncta engulfed by autophagosomes (Figures 5A and 5B), in agreement with previous studies.[21] In comparison, in GFP-p62^{C289,290S}-expressing cells, fewer p62 puncta were recruited to autophagosomes upon autophagy induction. To exclude the possibility that such changes of p62 co-localization with LC3 were due to impairment of p62-LC3 interaction, we performed a coIP analysis of p62 WT or p62^{C289,290S} and LC3. We observed that HA-tagged LC3 co-immunoprecipitated the same amount of FLAG-p62 WT and FLAG-p62^{C289,290S}, suggesting that *S*-acylation has no effect on p62-LC3 interaction (Figure S5A).

We next performed co-localization analysis of p62 puncta and LC3-positive membranes in the presence or absence of ZDHHC19. The expression of ZDHHC19, but not ZDHHC19^{C142S}, induced an increase in LC3-positive endogenous p62 puncta in HEK293 cells stably expressing mCherry-LC3 (Figures 5C and 5D). In *ZDHHC19*-KO cells, endogenous p62 formed abundant large puncta that were LC3-negative upon autophagy induction (Figures 5E and 5F). In addition, we observed small LC3-positive p62 puncta in GFP-p62 WT-expressing cells upon HA-ZDHHC19 expression, while it was difficult to establish LC3 localization owing to the large p62 puncta in GFP-p62^{C289,290S}-expressing cells (Figure 5G). The large p62 WT puncta adhered to LC3 dots, whereas it was difficult to observe apparent LC3 dots on large p62^{C289,290S} puncta (Figure S5B; Videos S1 and S2). Consistently, we found that, compared with GFP-p62 WT, more GFP-p62^{C289,290S} accumulated in the detergent-insoluble fraction (Figure S5C). Similarly, *ZDHHC19*-KO led to p62 accumulation in the detergent-insoluble fraction (Figure S5D). We then tested whether *S*-acylation regulates the liquid-like state of p62 by fluorescence recovery after photobleaching (FRAP), commonly used to measure condensate fluidity.[63] We observed that GFP-p62 had a faster recovery time than GFP-p62^{C289,290S} (Figure 5H), suggesting that *S*-acylation could regulate physicochemical behavior of p62-liquid droplets. Based on above results, we conclude that *S*-acylation mediates the association of p62 bodies with autophagic membranes, leading to formation of smaller LC3-positive p62 condensates.

To better understand the effect of *S*-acylation on autophagic membrane localization of p62 droplets, we determined where ZDHHC19-mediated p62 *S*-acylation occurs. ZDHHC19 itself was strongly co-localized with endoplasmic reticulum (ER) membranes as well as autophagic membranes (Figures S5E–S5I). Furthermore, we observed that autophagy inhibition by *FIP200* deletion caused an accumulation of *S*-acylated p62 in HeLa and mouse embryonic fibroblast (MEF) cells (Figures S5J–S5M), indicating that ER-localized ZDHHC19 mediates p62 *S*-acylation in the ER, and then follows p62 to autophagic membranes.

These results suggest that ZDHHC19-mediated *S*-acylation enhances autophagic membrane localization of p62 droplets, facilitating efficient degradation of their substrates.

S-acylation enhances the affinity of p62 for LC3-positive membranes *in vitro*

To directly test whether *S*-acylation promotes the interaction of p62 with LC3-positive membranes, we performed *in vitro* assays using acylated p62 protein and membrane mimics. Using a bioconjugation strategy, a palmitate with a maleimide moiety was reacted with purified recombinant protein to produce p62 protein-palmitate conjugates to mimic acylated proteins (Figure S6A), which is widely used to study the function of lipidated

(G) Representative images of p62 puncta and autophagosome in GFP-p62 WT or GFP-p62^{C289,290S} cells upon ZDHHC19 expression. *p62*-KO NRK cells stably expressing GFP-p62 WT or GFP-p62^{C289,290S} (green) were transfected with HA-ZDHHC19 and immunostained with anti-LC3A/B antibody (red) and anti-HA antibody (purple). Scale bars, 10 μm.

(H) Fluorescence recovery after photobleaching (FRAP) assays of GFP-p62 WT and GFP-p62^{C289,290S} puncta. Data from four independent experiments were analyzed. Scale bars, 1 μm.

See also Figure S5.

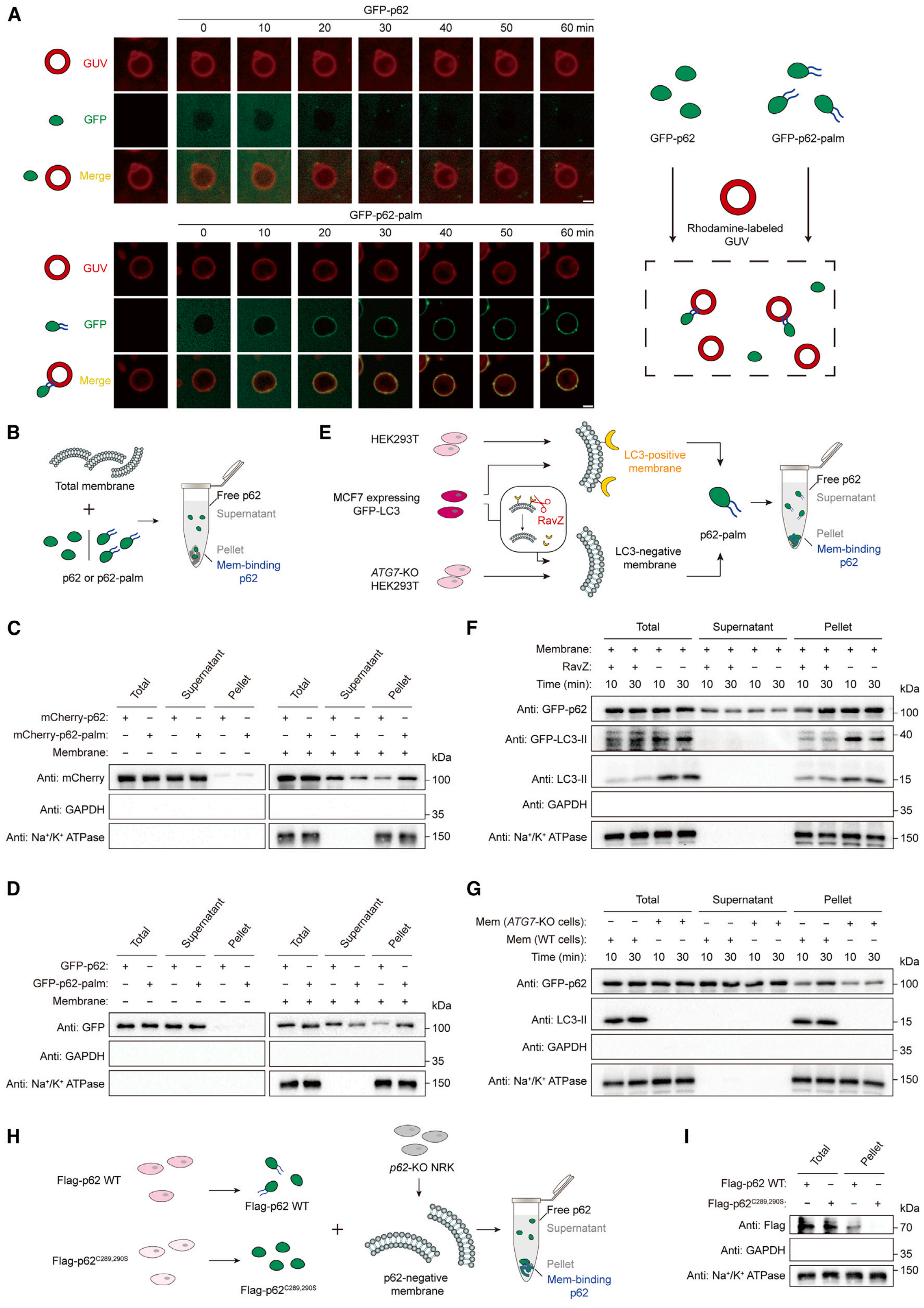

(legend on next page)

CellPress
OPEN ACCESS

proteins *in vitro*.[41,64,65] Site-specific modification of fluorescein isothiocyanate (FITC)-labeled p62 (279–300) peptide or GFP/mCherry-tagged p62 (155–440) protein was thereby achieved (Figures S6B–S6E).

We first investigated whether *S*-acylation promotes the interaction of p62 with membranes. Giant unilamellar vesicles (GUVs) are artificial membrane-mimetic systems used for the study of membrane-related phenomena. The system is characterized by its unilamellar lipid bilayer and micrometer-wide globular shape, allowing for easy observation of membrane interactions by microscopy. We observed that FITC-labeled p62 (279–300)-palmitate peptide was recruited to functionalized GUV membranes containing a red fluorescence rhodamine dye within 60 min, whereas the unmodified p62 peptide did not bind to GUVs (Figure S6F; Video S3). Similarly, acylated GFP-tagged p62 (155–440), but not the unmodified protein, was recruited to GUVs (Figure 6A; Video S4). In addition, we evaluated the affinity of acylated p62 protein to total cellular membranes *in vitro* (Figure 6B). We observed that while unmodified GFP/mCherry-p62 proteins targeted the membrane inefficiently, most acylated p62 proteins were recruited to cellular membranes, with the protein level in the supernatant decreasing accordingly (Figures 6C and 6D). These results suggest that acylation enhances the affinity of p62 protein to artificial membranes and total cellular membranes. In addition, we found that the association of p62 to isolated cellular membranes was faster than to artificial GUVs, indicating that other membrane-associated proteins or membrane composition has an impact on membrane association of p62.

We next evaluated the interaction between acylated p62 and LC3-positive or LC3-negative membranes. To this purpose, we treated total cellular membranes with the recombinant bacterial enzyme RavZ that can irreversibly delipidate membrane-associated LC3-II on autophagic membranes and release LC3 protein from membranes (Figures 6E and S6D).[66,67] We observed that more acylated p62 protein accumulated to total cellular membranes than that of RavZ-treated membranes (Figure 6F). Moreover, total cellular membranes were isolated from HEK293T WT cells or *ATG7*-KO HEK293T cells (Figure 6E). There was no LC3-II protein in the cell membranes from *ATG7*-KO HEK293T cells, because E1-like ATG7 deficiency prevents production of LC3-II in these cells. We observed that acylated p62 accumulated to the total cellular membranes from HEK293T WT cells but not to membranes from *ATG7*-KO cells (Figure 6G). These results suggest that acylated p62 has higher affinity for LC3-positive membranes than that for LC3-negative membranes.

To further confirm the binding of *S*-acylated p62 and membranes, we reconstituted *S*-acylated p62 in cellular membranes *in vitro* (Figure 6H). To this purpose, we purified *S*-acylated p62 and non-acylated p62 from HEK293T transiently expressing FLAG-p62 WT and FLAG-p62^{C289,290S}, respectively, and isolated total cellular membrane from *p62*-KO NRK cells. On incubation with total cellular membrane, more acylated p62 proteins were accumulated than non-acylated p62 (Figure 6I).

Therefore, these *in vitro* results are consistent with an increased affinity of acylated p62 to LC3-positive membranes.

APT1 deacylates p62

Deacylation, the reversal of *S*-acylation, is mediated by acyl protein thioesterases (APTs).[68] Previous studies have identified several mammalian deacylases, including APTs (APT1 and APT2), palmitoyl protein thioesterases 1 and 2 (PPT1 and PPT2), and the diverse α/β-hydrolase fold domain (ABHD) family of proteins.[69,70] To identify the enzyme that catalyzes the deacylation of p62, we expressed five known APTs in MCF7 cells and detected the *S*-acylation level of endogenous p62 by ABE assay. We found that expression of APT1, but not other APTs, significantly decreased endogenous p62 *S*-acylation (Figure 7A).

Given that p62 *S*-acylation facilitates p62 degradation, we then investigated the role of APT1-mediated p62 deacylation in the regulation of p62-mediated selective autophagy. We first checked p62 protein levels upon APT1 expression or in *APT1*-KO cells (Figures S7A–S7C). We observed that APT1 expression inhibited p62 turnover and degradation of ubiquitinated proteins (Figures 7B–7D). It is noted that Baf-A1 treatment increased levels of p62, but not ubiquitinated proteins, in cells expressing the APT1^{S119A} mutant, suggesting that APT1 could affect ubiquitinated protein degradation in a manner partially independent of p62-mediated autophagy. Compared with control WT cells, p62 protein level was decreased in *APT1*-KO cells, which was recovered by re-expressing APT1 but not catalytically inactive mutant APT1^{S119A} (Figures 7E and 7F). We then determined the effect of

Figure 6. *S*-acylation enhances the affinity of p62 for LC3-positive membranes *in vitro*

(A) Left, representative images of GFP-p62-positive GUVs. GFP-p62 (155–440) or GFP-p62 (155–440)-palm (green) was incubated with rhodamine labeled GUVs (red) for 0–60 min. The fluorescence was monitored by laser scanning confocal microscope. Right, schematic diagram of p62 protein recruitment to GUVs. GFP-p62 (155–440)-palm, palmitate-maleimide conjugated MBP-GFP-p62 (155–440)^{C331S}. Scale bars, 4 μm.

(B) Schematic diagram of p62 co-precipitated with total cellular membrane *in vitro*.

(C) Immunoblot analysis of mCherry-p62 and mCherry-p62-palm proteins co-precipitated with total cellular membrane *in vitro* as in (B). The levels of Na^+/K^+ ATPase and GADPH were detected as internal references for total cellular membrane and cytoplasm, respectively.

(D) Immunoblot analysis of GFP-p62 and GFP-p62-palm proteins co-precipitated with total cellular membrane *in vitro* as in (B).

(E) Schematic diagram of GFP-p62-palm co-precipitated with LC3-positive or LC3-negative membranes.

(F) Immunoblot analysis of GFP-p62-palm co-precipitated with LC3-rich and RavZ-treated membrane *in vitro* as in (E).

(G) Immunoblot analysis of GFP-p62-palm co-precipitated with LC3-positive and LC3-negative membrane from *in vitro ATG7*-KO HEK293T cells as in (E).

(H) Schematic diagram of reconstitution of *S*-acylated p62 in total cellular membrane *in vitro*. *S*-acylated p62 and non-acylated p62 proteins were purified from *p62*-KO NRK cells transiently expressing FLAG-p62 WT and FLAG-p62^{C289,290S}, respectively. The total cellular membranes were prepared from *p62*-KO NRK cells and further incubated with the purified p62 proteins.

(I) Immunoblot analysis of mammalian cell-expressed FLAG-p62 WT and FLAG-p62^{C289,290S} proteins co-precipitated with total cellular membrane *in vitro* as in (H).

See also Figure S6.

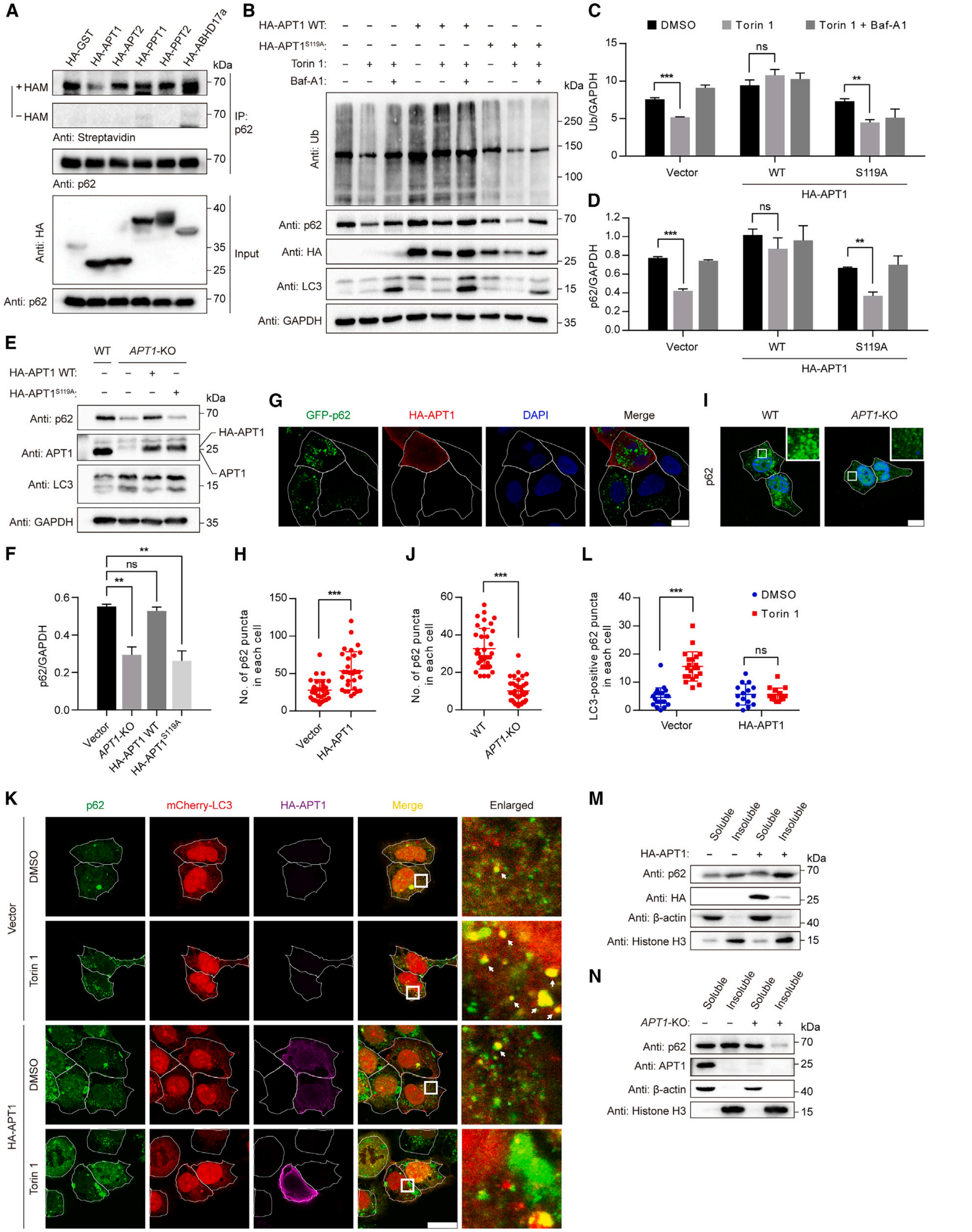

(legend on next page)

APT1 on the size distribution of p62 puncta. APT1 expression induced the accumulation of large p62 puncta (Figures 7G and 7H), whereas *APT1*-KO reduced the number of p62 puncta (Figures 7I and 7J). Additionally, APT1 expression suppressed autophagic membrane localization of p62 puncta (Figures 7K and 7L). We next analyzed the effect of APT1 on detergent-insoluble p62 levels. We found that APT1 expression induced the accumulation of detergent-insoluble p62 (Figure 7M), whereas *APT1*-KO decreased the amount of detergent-insoluble p62 (Figure 7N). Furthermore, lysosomal inhibition led to reduced accumulation of ubiquitinated proteins upon APT1 expression (Figures S7D and S7E), whereas *APT1*-KO increased the accumulation of ubiquitinated proteins (Figures S7F and S7G). These results suggest that APT1 suppresses p62-mediated selective autophagy.

Taken together, we conclude that APT1 deacylates p62 and regulates its function by suppressing p62-mediated selective autophagy.

DISCUSSION

Previous studies have shown that p62-mediated selective autophagy is regulated by several posttranslational modifications of p62, such as phosphorylation, acetylation, and ubiquitination.[6,28,30] In this study, we revealed a posttranslational modification of p62 with long-chain fatty acids, known as *S*-acylation. We have demonstrated that *S*-acylation of p62 enhances the affinity of p62 for LC3-positive membranes and promotes p62 droplet recruitment to autophagosomes. *S*-acylation of p62 is dynamically regulated by *S*-acyltransferase ZDHHC19 and APT1. These data highlight the importance of *S*-acylation in regulating p62-mediated selective autophagic clearance of ubiquitinated proteins in response to proteotoxic stress. Specifically, p62 *S*-acylation facilitates entry of p62 droplets into autophagosomes for degradation, hence enhancing p62-mediated selective autophagic flux. *S*-acylation promotes autophagic membrane localization of p62 droplets and the formation of small LC3-positive p62 droplets. Our study shows the functional significance of the *S*-acylation-deacylation cycle in regulating p62 droplet recruitment to the autophagic membrane, advancing our understanding of the molecular mechanism and regulatory network of p62-mediated selective autophagy.

Autophagy is a highly dynamic intracellular trafficking process and involves various membrane-related events that are tightly regulated by autophagy-related proteins.[71] Recently, proteomic studies of *S*-acylated proteins showed that many autophagy-related proteins are putatively acylated.[72] Our work will open up a field of investigation on the mechanism of autophagy-related protein interactions with autophagic membranes.

S-acylation regulates p62 droplet association with autophagic membrane

S-acylation has previously been shown to increase the hydrophobicity of proteins and to contribute to their membrane association. Indeed, our results suggest that *S*-acylation enhances the association of p62 to membrane mimics such as artificial membranes and isolated cellular membranes. We were also able to reconstitute *S*-acylated p62 in cellular membranes *in vitro*, enabling us to show that acylated p62 associated more with LC3-positive membranes than with LC3-negative membranes, which suggests that membrane-associated LC3 assists the interaction between *S*-acylated p62 and membranes *in vitro*.

Our results also show that deficiency or a decrease in p62 *S*-acylation results in the formation of large LC3-negative p62 puncta in cells, indicating that *S*-acylation plays an important role in regulating p62 droplet engulfment by the autophagic membrane. It is known that p62 binds directly to LC3 via its LIR motif to facilitate the degradation of ubiquitinated protein aggregates by autophagy.[22] It seems that in response to ubiquitin proteotoxic stress, p62 is *S*-acylated by the ER membrane-associated ZDHHC19; subsequently, p62 links to the autophagic membrane by its lipid and LIR motif. *S*-acylation-mediated association of p62 droplets with the autophagic membrane coupled

Figure 7. APT1 mediates deacylation of p62

(A) *S*-acylation of endogenous p62 detected by ABE assay in the presence of various thioesterases.

(B) Immunoblot analysis of p62 and total polyubiquitinated proteins in HEK293T cells transiently expressing HA-APT1 WT or HA-APT1^{S119A}. Cells were transfected with HA-APT1 WT or HA-APT1^{S119A} and then incubated with Torin 1 (1 μM) for 4 h, with or without Baf-A1 (1 μM).

(C) Quantification of total ubiquitinated proteins as in (B). Data represent the mean ± SEM of three independent experiments. ns, no significant difference; $^{**}p < 0.01$; $^{***}p < 0.001$; Student's t test.

(D) Quantification of p62 protein levels as in (B). Data represent the mean ± SEM of three independent experiments. ns, no significant difference; $^{**}p < 0.01$; $^{***}p < 0.001$; Student's t test.

(E) Immunoblot analysis of p62 protein in *APT1*-KO HEK293T cells expressing empty vector, HA-APT1 WT, or HA-APT1^{S119A}.

(F) Quantification of the p62 protein level as in (E). Data represent the mean ± SEM of three independent experiments. ns, no significant difference; $^{**}p < 0.01$; Student's t test.

(G) Representative images of p62 puncta in GFP-p62-expressing *p62*-KO NRK cells upon HA-APT1 expression. GFP-p62 NRK cells (green) were transiently transfected with HA-APT1 and immunostained with anti-HA antibody (red). Scale bars, 10 μm.

(H) Quantification of the number of p62 puncta in each cell as in (G). $^{***}p < 0.001$, Student's t test.

(I) Representative images of p62 puncta in *APT1*-KO HEK293T cells. Scale bars, 10 μm.

(J) Quantification of the number of p62 puncta in each cell as in (I). $^{***}p < 0.001$, Student's t test.

(K) Representative images of p62- and LC3-positive puncta in mCherry-LC3-expressing HEK293 cells transiently expressing HA-APT1. Cells were incubated with Torin 1 (1 μM) for 1 h, followed by immunostaining with anti-p62 antibody (green) and anti-HA antibody (purple). Scale bars, 10 μm.

(L) Quantification of the number of LC3-positive p62 puncta in each cell as in (K). ns, no significant difference; $^{***}p < 0.001$; Student's t test.

(M) Analysis of Triton X-100 soluble and insoluble fractions of p62 in HEK293T cells transiently expressing HA-APT1.

(N) Analysis of Triton X-100 soluble and insoluble fractions of p62 in *APT1*-KO cells.

See also Figure S7.

with the p62-LC3 interaction could yield enhanced affinity for p62 droplets and the autophagic membrane, thereby leading to the efficient recruitment of p62 droplets by the autophagic membrane. This is an example of *S*-acylation in regulating biomolecular condensate association with membrane.

S-acylation facilitates deformation of p62 droplets by autophagic membrane

Intracellular wetting mediates contacts between p62 droplets and autophagosomal membranes, leading to formation of multiple small LC3 puncta corresponding to autophagosomal membranes on the surface of these droplets.[60,62,73] Supporting this notion, we observed small LC3-positive puncta upon autophagy induction. Deficiency or decreasing p62 *S*-acylation results in the production of large p62 puncta, whereas increasing p62 *S*-acylation promotes the formation of small LC3-positive p62 droplets. These findings are consistent with a model whereby ZDHHC19-mediated p62 *S*-acylation promotes autophagic membrane localization of p62 droplets and enhances the interaction of p62 droplets with autophagic membranes. We propose that *S*-acylation enhances autophagic membrane association of p62 droplets and hence promotes wetting of p62 droplets with the autophagic membrane, thereby contributing to the production of small LC3-positive p62 droplets and efficient degradation of their substrates.

Increasing p62 acylation enhances p62-mediated selective autophagic flux

There is mounting evidence that dysregulation of selective autophagic clearance of protein aggregates is linked to a variety of pathological processes, including neurodegenerative diseases.[10,11,74,75] Therefore, inducing selective autophagy has potential therapeutic benefits for such diseases.[76–78] Most chemical inducers targeting common autophagy pathways activate selective autophagy; however, these chemical inducers also trigger basal autophagy activity, thereby leading to unexpected side effects.[79,80] Targeting specific components of selective autophagy machineries would be a promising alternative for the development of new autophagy inducers.[81] Our results indicate that increasing p62 acylation by upregulation of ZDHHC19 or genetic KO of APT1 accelerates selective autophagy by promoting the entry of ubiquitinated substrates into autophagy but has no effect on the level of LC3-II (Figures 7H and S7A), suggesting that increasing p62 acylation shows high selectivity for autophagic clearance of ubiquitinated proteins. Therefore, our study provides a strategy to specifically enhance selective autophagic clearance of protein aggregates. A recent report showed that APT1 inhibitor restores Huntington's disease (HD) by increasing brain palmitoylation,[82] which is line with our notion that APT1 inhibition could provide a potential strategy against neurodegenerative diseases.

Limitations of the study

Our study provides strong evidence that *S*-acylation enhances the association of p62 to LC3-positive membranes and further promotes p62 droplet recruitment into autophagosomes. p62 is *S*-acylated by the ER membrane-associated ZDHHC19, but exactly how *S*-acylated p62 droplets arrive at autophagosomes remains to be explored. In addition, in the absence of p62, ER membrane-associated ZDHHC19 was still strongly co-localized with autophagic membranes, and ZDHHC19 and the catalytically inactive mutant ZDHHC19^{C142S} were still degraded by autophagy. Although there was no apparent change in LC3-II level upon expression or deficiency of ZDHHC19, future research should explore other substrates and non-catalytic functions of ZDHHC19 in autophagy. Finally, while this research highlights the significance of p62 *S*-acylation in cell survival under stress, future studies should investigate the functional significance in animal models.

STAR★METHODS

Detailed methods are provided in the online version of this paper and include the following:

- KEY RESOURCES TABLE
- RESOURCE AVAILABILITY
  - Lead contact
  - Materials availability
  - Data and code availability
- EXPERIMENTAL MODEL AND STUDY PARTICIPANT DETAILS
  - E. coli strains and media
  - Cell lines
- METHOD DETAILS
  - Plasmid constructions and cloning
  - Western blotting
  - Acyl-biotin exchange (ABE) assay
  - LC–MS of HAM-mediated hydrolysate
  - Metabolic labeling and click chemistry
  - Proximity labeling
  - Immunofluorescence
  - Microscopy
  - Biochemical fractionation
  - Immunoprecipitation analysis
  - Protein expression and purification
  - Peptide synthesis and purification
  - Synthesis of palmitate-maleimide
  - Preparation of protein/peptide conjugates
  - GUV assay
  - Preparation of membrane fraction
  - Co-precipitation assay
  - FRAP analysis
  - Non-reduced SDS-PAGE
  - Cell death measurement
- QUANTIFICATION AND STATISTICAL ANALYSIS

SUPPLEMENTAL INFORMATION

Supplemental information can be found online at https://doi.org/10.1016/j.molcel.2023.09.004.

ACKNOWLEDGMENTS

We thank Janine Gray (Imperial College London), Quan Chen (Nankai University), and Feng Shao (National Institute of Biological Sciences, Beijing) for critical reading of the manuscript; Li Yu (Tsinghua University) for WT and *p62*-KO NRK cells; Kefeng Lu (Sichuan University) for *ATG7*-KO HEK293T cells; Min Li

CellPress
OPEN ACCESS

(Sun Yat-Sen University) for *FIP200*-KO HeLa cells; Prof. Yueguang Rong (Huazhong University of Science and Technology) for *FIP200*-KO MEF cells; Masaki Fukata (National Institute for Physiological Sciences) for the expression vectors of the ZDHHC proteins; and Jieqiong Tan (Central South University) for the plasmid encoding human p62. We are also thankful for the technical support by the Analytical and Testing Center of Chongqing University. This study was supported by the National Natural Science Foundation of China (NSFC) (91854101, 32170185, and 31801166), the Natural Science Foundation of Chongqing, China (CSTB2022NSCQ-MSX0463), and the Venture and Innovation Support Program for Chongqing Overseas Returnees grant cx2022066 to A.Y.; the Fundamental Research Funds for the Central Universities (2022CDJYGRH-002) to A.Y. and T. X.; NSFC and the Royal Society (RS) joint exchange program (22011530161 and IEC\\NSFC\191085) to A.Y. and E.W.T.; and Cancer Research UK (DRCNPG-Nov21\100001, with support from the Engineering & Physical Sciences Research Council) to E.W.T.

AUTHOR CONTRIBUTIONS

A.Y. conceived the study and supervised the project; A.Y., E.W.T., and X.H. wrote the manuscript; X.H., J.Y., L.L., and J.C. performed most of the experiments. E.K. and J.H. contributed to the construction of *ZDHHC19*-KO and *APT1*-KO cells. S.Z. and D.L. contributed to the synthesis of palmitate-maleimide. Q.S. and X.W. contributed to GUV experiments. L.M., C.L., X.C., Y.Y., S.O., F.W., and Z.W. contributed to parts of the experiments. E.W.T., E.K., Q.S., D.N., and T.X. provided insightful suggestions. All the authors discussed the results and commented on the manuscript.

DECLARATION OF INTERESTS

E.W.T. is a founder and shareholder in Myricx Pharma Ltd. and receives consultancy or research funding from Kura Oncology, Pfizer Ltd., Samsara Therapeutics, Myricx Pharma Ltd., MSD, Exscientia, and Daiichi Sankyo.

Received: November 22, 2022
Revised: June 22, 2023
Accepted: September 7, 2023
Published: October 5, 2023

REFERENCES

1. Pohl, C., and Dikic, I. (2019). Cellular quality control by the ubiquitin-proteasome system and autophagy. Science *366*, 818–822. https://doi.org/10.1126/science.aax3769.
2. Varshavsky, A. (2017). The ubiquitin system, autophagy, and regulated protein degradation. Annu. Rev. Biochem. *86*, 123–128. https://doi.org/10.1146/annurev-biochem-061516-044859.
3. Dikic, I. (2017). Proteasomal and autophagic degradation systems. Annu. Rev. Biochem. *86*, 193–224. https://doi.org/10.1146/annurev-biochem-061516-044908.
4. Ohsumi, Y. (2014). Historical landmarks of autophagy research. Cell Res. *24*, 9–23. https://doi.org/10.1038/cr.2013.169.
5. Mizushima, N. (2018). A brief history of autophagy from cell biology to physiology and disease. Nat. Cell Biol. *20*, 521–527. https://doi.org/10.1038/s41556-018-0092-5.
6. Matsumoto, G., Wada, K., Okuno, M., Kurosawa, M., and Nukina, N. (2011). Serine 403 phosphorylation of p62/SQSTM1 regulates selective autophagic clearance of ubiquitinated proteins. Mol. Cell *44*, 279–289. https://doi.org/10.1016/j.molcel.2011.07.039.
7. Korolchuk, V.I., Mansilla, A., Menzies, F.M., and Rubinsztein, D.C. (2009). Autophagy inhibition compromises degradation of ubiquitin-proteasome pathway substrates. Mol. Cell *33*, 517–527. https://doi.org/10.1016/j.molcel.2009.01.021.
8. Balch, W.E., Morimoto, R.I., Dillin, A., and Kelly, J.W. (2008). Adapting proteostasis for disease intervention. Science *319*, 916–919. https://doi.org/10.1126/science.1141448.
9. Levy, J.M.M., Towers, C.G., and Thorburn, A. (2017). Targeting autophagy in cancer. Nat. Rev. Cancer *17*, 528–542. https://doi.org/10.1038/nrc.2017.53.
10. Boland, B., Yu, W.H., Corti, O., Mollereau, B., Henriques, A., Bezard, E., Pastores, G.M., Rubinsztein, D.C., Nixon, R.A., Duchen, M.R., et al. (2018). Promoting the clearance of neurotoxic proteins in neurodegenerative disorders of ageing. Nat. Rev. Drug Discov. *17*, 660–688. https://doi.org/10.1038/nrd.2018.109.
11. Dikic, I., and Elazar, Z. (2018). Mechanism and medical implications of mammalian autophagy. Nat. Rev. Mol. Cell Biol. *19*, 349–364. https://doi.org/10.1038/s41580-018-0003-4.
12. Gatica, D., Lahiri, V., and Klionsky, D.J. (2018). Cargo recognition and degradation by selective autophagy. Nat. Cell Biol. *20*, 233–242. https://doi.org/10.1038/s41556-018-0037-z.
13. Levine, B., and Kroemer, G. (2019). Biological functions of autophagy genes: a disease perspective. Cell *176*, 11–42. https://doi.org/10.1016/j.cell.2018.09.048.
14. Lamark, T., and Johansen, T. (2021). Mechanisms of selective autophagy. Annu. Rev. Cell Dev. Biol. *37*, 143–169. https://doi.org/10.1146/annurev-cellbio-120219-035530.
15. Moscat, J., Karin, M., and Diaz-Meco, M.T. (2016). p62 in cancer: signaling adaptor beyond autophagy. Cell *167*, 606–609. https://doi.org/10.1016/j.cell.2016.09.030.
16. Katsuragi, Y., Ichimura, Y., and Komatsu, M. (2015). p62/SQSTM1 functions as a signaling hub and an autophagy adaptor. FEBS J. *282*, 4672–4678. https://doi.org/10.1111/febs.13540.
17. Kumar, A.V., Mills, J., and Lapierre, L.R. (2022). Selective autophagy receptor p62/SQSTM1, a pivotal player in stress and aging. Front. Cell Dev. Biol. *10*, 793328. https://doi.org/10.3389/fcell.2022.793328.
18. Berkamp, S., Mostafavi, S., and Sachse, C. (2021). Structure and function of p62/SQSTM1 in the emerging framework of phase separation. FEBS J. *288*, 6927–6941. https://doi.org/10.1111/febs.15672.
19. Clausen, T.H., Lamark, T., Isakson, P., Finley, K., Larsen, K.B., Brech, A., Øvervatn, A., Stenmark, H., Bjørkøy, G., Simonsen, A., and Johansen, T. (2010). p62/SQSTM1 and ALFY interact to facilitate the formation of p62 bodies/ALIS and their degradation by autophagy. Autophagy *6*, 330–344. https://doi.org/10.4161/auto.6.3.11226.
20. Isogai, S., Morimoto, D., Arita, K., Unzai, S., Tenno, T., Hasegawa, J., Sou, Y.S., Komatsu, M., Tanaka, K., Shirakawa, M., and Tochio, H. (2011). Crystal structure of the ubiquitin-associated (UBA) domain of p62 and its interaction with ubiquitin. J. Biol. Chem. *286*, 31864–31874. https://doi.org/10.1074/jbc.M111.259630.
21. Bjørkøy, G., Lamark, T., Brech, A., Outzen, H., Perander, M., Overvatn, A., Stenmark, H., and Johansen, T. (2005). p62/SQSTM1 forms protein aggregates degraded by autophagy and has a protective effect on huntingtin-induced cell death. J. Cell Biol. *171*, 603–614. https://doi.org/10.1083/jcb.200507002.
22. Pankiv, S., Clausen, T.H., Lamark, T., Brech, A., Bruun, J.A., Outzen, H., Øvervatn, A., Bjørkøy, G., and Johansen, T. (2007). p62/SQSTM1 binds directly to Atg8/LC3 to facilitate degradation of ubiquitinated protein aggregates by autophagy. J. Biol. Chem. *282*, 24131–24145. https://doi.org/10.1074/jbc.M702824200.
23. Ichimura, Y., Kumanomidou, T., Sou, Y.S., Mizushima, T., Ezaki, J., Ueno, T., Kominami, E., Yamane, T., Tanaka, K., and Komatsu, M. (2008). Structural basis for sorting mechanism of p62 in selective autophagy. J. Biol. Chem. *283*, 22847–22857. https://doi.org/10.1074/jbc.M802182200.
24. Sun, D., Wu, R., Zheng, J., Li, P., and Yu, L. (2018). Polyubiquitin chain-induced p62 phase separation drives autophagic cargo segregation. Cell Res. *28*, 405–415. https://doi.org/10.1038/s41422-018-0017-7.
25. Zaffagnini, G., Savova, A., Danieli, A., Romanov, J., Tremel, S., Ebner, M., Peterbauer, T., Sztacho, M., Trapannone, R., Tarafder, A.K., et al. (2018).

p62 filaments capture and present ubiquitinated cargos for autophagy. EMBO J. *37*, e98308. https://doi.org/10.15252/embj.201798308.

26. Kageyama, S., Gudmundsson, S.R., Sou, Y.S., Ichimura, Y., Tamura, N., Kazuno, S., Ueno, T., Miura, Y., Noshiro, D., Abe, M., et al. (2021). p62/SQSTM1-droplet serves as a platform for autophagosome formation and anti-oxidative stress response. Nat. Commun. *12*, 16. https://doi.org/10.1038/s41467-020-20185-1.
27. Danieli, A., and Martens, S. (2018). p62-mediated phase separation at the intersection of the ubiquitin-proteasome system and autophagy. J. Cell Sci. *131*, jcs214304. https://doi.org/10.1242/jcs.214304.
28. You, Z., Jiang, W.X., Qin, L.Y., Gong, Z., Wan, W., Li, J., Wang, Y., Zhang, H., Peng, C., Zhou, T., et al. (2019). Requirement for p62 acetylation in the aggregation of ubiquitylated proteins under nutrient stress. Nat. Commun. *10*, 5792. https://doi.org/10.1038/s41467-019-13718-w.
29. Peng, H., Yang, J., Li, G., You, Q., Han, W., Li, T., Gao, D., Xie, X., Lee, B.H., Du, J., et al. (2017). Ubiquitylation of p62/sequestosome1 activates its autophagy receptor function and controls selective autophagy upon ubiquitin stress. Cell Res. *27*, 657–674. https://doi.org/10.1038/cr.2017.40.
30. Lee, Y., Chou, T.F., Pittman, S.K., Keith, A.L., Razani, B., and Weihl, C.C. (2017). Keap1/Cullin3 modulates p62/SQSTM1 activity via UBA domain ubiquitination. Cell Rep. *20*, 1994. https://doi.org/10.1016/j.celrep.2017.08.019.
31. Yang, Y., Willis, T.L., Button, R.W., Strang, C.J., Fu, Y., Wen, X., Grayson, P.R.C., Evans, T., Sipthorpe, R.J., Roberts, S.L., et al. (2019). Cytoplasmic DAXX drives SQSTM1/p62 phase condensation to activate Nrf2-mediated stress response. Nat. Commun. *10*, 3759. https://doi.org/10.1038/s41467-019-11671-2.
32. Feng, X., Du, W., Ding, M., Zhao, W., Xirefu, X., Ma, M., Zhuang, Y., Fu, X., Shen, J., Zhang, J., et al. (2022). Myosin 1D and the branched actin network control the condensation of p62 bodies. Cell Res. *32*, 659–669. https://doi.org/10.1038/s41422-022-00662-6.
33. Tan, C.T., Chang, H.C., Zhou, Q., Yu, C., Fu, N.Y., Sabapathy, K., and Yu, V.C. (2021). MOAP-1-mediated dissociation of p62/SQSTM1 bodies releases Keap1 and suppresses Nrf2 signaling. EMBO Rep. *22*, e50854. https://doi.org/10.15252/embr.202050854.
34. Jiang, H., Zhang, X., Chen, X., Aramsangtienchai, P., Tong, Z., and Lin, H. (2018). Protein lipidation: occurrence, mechanisms, biological functions, and enabling technologies. Chem. Rev. *118*, 919–988. https://doi.org/10.1021/acs.chemrev.6b00750.
35. Lanyon-Hogg, T., Faronato, M., Serwa, R.A., and Tate, E.W. (2017). Dynamic protein acylation: new substrates, mechanisms, and drug targets. Trends Biochem. Sci. *42*, 566–581. https://doi.org/10.1016/j.tibs.2017.04.004.
36. Lu, Y., Zheng, Y., Coyaud, É., Zhang, C., Selvabaskaran, A., Yu, Y., Xu, Z., Weng, X., Chen, J.S., Meng, Y., et al. (2019). Palmitoylation of NOD1 and NOD2 is required for bacterial sensing. Science *366*, 460–467. https://doi.org/10.1126/science.aau6391.
37. Zhang, M., Zhou, L., Xu, Y., Yang, M., Xu, Y., Komaniecki, G.P., Kosciuk, T., Chen, X., Lu, X., Zou, X., et al. (2020). A STAT3 palmitoylation cycle promotes TH17 differentiation and colitis. Nature *586*, 434–439. https://doi.org/10.1038/s41586-020-2799-2.
38. Chamberlain, L.H., and Shipston, M.J. (2015). The physiology of protein S-acylation. Physiol. Rev. *95*, 341–376. https://doi.org/10.1152/physrev.00032.2014.
39. Das, T., Yount, J.S., and Hang, H.C. (2021). Protein S-palmitoylation in immunity. Open Biol. *11*, 200411. https://doi.org/10.1098/rsob.200411.
40. Main, A., and Fuller, W. (2022). Protein S-palmitoylation: advances and challenges in studying a therapeutically important lipid modification. FEBS J. *289*, 861–882. https://doi.org/10.1111/febs.15781.
41. Yang, A., Zhao, L., and Wu, Y.W. (2015). Chemical synthesis and biological function of lipidated proteins. Top. Curr. Chem. *362*, 137–182. https://doi.org/10.1007/128_2014_582.
42. Roth, A.F., Wan, J., Bailey, A.O., Sun, B., Kuchar, J.A., Green, W.N., Phinney, B.S., Yates, J.R., 3rd, and Davis, N.G. (2006). Global analysis of protein palmitoylation in yeast. Cell *125*, 1003–1013. https://doi.org/10.1016/j.cell.2006.03.042.
43. Mariscal, J., Vagner, T., Kim, M., Zhou, B., Chin, A., Zandian, M., Freeman, M.R., You, S., Zijlstra, A., Yang, W., and Di Vizio, D. (2020). Comprehensive palmitoyl-proteomic analysis identifies distinct protein signatures for large and small cancer-derived extracellular vesicles. J. Extracell. Vesicles *9*, 1764192. https://doi.org/10.1080/20013078.2020.1764192.
44. Thinon, E., Fernandez, J.P., Molina, H., and Hang, H.C. (2018). Selective enrichment and direct analysis of protein S-palmitoylation sites. J. Proteome Res. *17*, 1907–1922. https://doi.org/10.1021/acs.jproteome.8b00002.
45. Martin, B.R., and Cravatt, B.F. (2009). Large-scale profiling of protein palmitoylation in mammalian cells. Nat. Methods *6*, 135–138. https://doi.org/10.1038/nmeth.1293.
46. Ning, W., Jiang, P., Guo, Y., Wang, C., Tan, X., Zhang, W., Peng, D., and Xue, Y. (2021). GPS-Palm: a deep learning-based graphic presentation system for the prediction of S-palmitoylation sites in proteins. Brief. Bioinform. *22*, 1836–1847. https://doi.org/10.1093/bib/bbaa038.
47. Ren, J., Wen, L., Gao, X., Jin, C., Xue, Y., and Yao, X. (2008). CSS-Palm 2.0: an updated software for palmitoylation sites prediction. Protein Eng. Des. Sel. *21*, 639–644. https://doi.org/10.1093/protein/gzn039.
48. Turco, E., Witt, M., Abert, C., Bock-Bierbaum, T., Su, M.Y., Trapannone, R., Sztacho, M., Danieli, A., Shi, X., Zaffagnini, G., et al. (2019). FIP200 claw domain binding to p62 promotes autophagosome formation at ubiquitin condensates. Mol. Cell *74*, 330–346.e11. https://doi.org/10.1016/j.molcel.2019.01.035.
49. Greaves, J., and Chamberlain, L.H. (2011). DHHC palmitoyl transferases: substrate interactions and (patho)physiology. Trends Biochem. Sci. *36*, 245–253. https://doi.org/10.1016/j.tibs.2011.01.003.
50. Rana, M.S., Kumar, P., Lee, C.J., Verardi, R., Rajashankar, K.R., and Banerjee, A. (2018). Fatty acyl recognition and transfer by an integral membrane S-acyltransferase. Science *359*. https://doi.org/10.1126/science.aao6326.
51. De, I., and Sadhukhan, S. (2018). Emerging roles of DHHC-mediated protein S-palmitoylation in physiological and pathophysiological context. Eur. J. Cell Biol. *97*, 319–338. https://doi.org/10.1016/j.ejcb.2018.03.005.
52. Jennings, B.C., and Linder, M.E. (2012). DHHC protein S-acyltransferases use similar ping-pong kinetic mechanisms but display different acyl-CoA specificities. J. Biol. Chem. *287*, 7236–7245. https://doi.org/10.1074/jbc.M111.337246.
53. Mitchell, D.A., Vasudevan, A., Linder, M.E., and Deschenes, R.J. (2006). Protein palmitoylation by a family of DHHC protein S-acyltransferases. J. Lipid Res. *47*, 1118–1127. https://doi.org/10.1194/jlr.R600007-JLR200.
54. Branon, T.C., Bosch, J.A., Sanchez, A.D., Udeshi, N.D., Svinkina, T., Carr, S.A., Feldman, J.L., Perrimon, N., and Ting, A.Y. (2018). Efficient proximity labeling in living cells and organisms with TurboID. Nat. Biotechnol. *36*, 880–887. https://doi.org/10.1038/nbt.4201.
55. Huang, X., Yao, J., Liu, L., Luo, Y., and Yang, A. (2022). Atg8-PE protein-based in vitro biochemical approaches to autophagy studies. Autophagy *18*, 2020–2035. https://doi.org/10.1080/15548627.2022.2025572.
56. Lamark, T., Svenning, S., and Johansen, T. (2017). Regulation of selective autophagy: the p62/SQSTM1 paradigm. Essays Biochem. *61*, 609–624. https://doi.org/10.1042/EBC20170035.
57. Komatsu, M., Waguri, S., Koike, M., Sou, Y.S., Ueno, T., Hara, T., Mizushima, N., Iwata, J., Ezaki, J., Murata, S., et al. (2007). Homeostatic levels of p62 control cytoplasmic inclusion body formation in autophagy-deficient mice. Cell *131*, 1149–1163. https://doi.org/10.1016/j.cell.2007.10.035.
58. Sarraf, S.A., Shah, H.V., Kanfer, G., Pickrell, A.M., Holtzclaw, L.A., Ward, M.E., and Youle, R.J. (2020). Loss of TAX1BP1-directed autophagy results

CellPress
OPEN ACCESS

in protein aggregate accumulation in the brain. Mol. Cell *80*, 779–795.e10. https://doi.org/10.1016/j.molcel.2020.10.041.

59. Jakobi, A.J., Huber, S.T., Mortensen, S.A., Schultz, S.W., Palara, A., Kuhm, T., Shrestha, B.K., Lamark, T., Hagen, W.J.H., Wilmanns, M., et al. (2020). Structural basis of p62/SQSTM1 helical filaments and their role in cellular cargo uptake. Nat. Commun. *11*, 440. https://doi.org/10.1038/s41467-020-14343-8.
60. Agudo-Canalejo, J., Schultz, S.W., Chino, H., Migliano, S.M., Saito, C., Koyama-Honda, I., Stenmark, H., Brech, A., May, A.I., Mizushima, N., and Knorr, R.L. (2021). Wetting regulates autophagy of phase-separated compartments and the cytosol. Nature *591*, 142–146. https://doi.org/10.1038/s41586-020-2992-3.
61. Fujioka, Y., Alam, J.M., Noshiro, D., Mouri, K., Ando, T., Okada, Y., May, A.I., Knorr, R.L., Suzuki, K., Ohsumi, Y., and Noda, N.N. (2020). Phase separation organizes the site of autophagosome formation. Nature *578*, 301–305. https://doi.org/10.1038/s41586-020-1977-6.
62. Kusumaatmaja, H., May, A.I., and Knorr, R.L. (2021). Intracellular wetting mediates contacts between liquid compartments and membrane-bound organelles. J. Cell Biol. *220*, e202103175. https://doi.org/10.1083/jcb.202103175.
63. Bracha, D., Walls, M.T., and Brangwynne, C.P. (2019). Probing and engineering liquid-phase organelles. Nat. Biotechnol. *37*, 1435–1445. https://doi.org/10.1038/s41587-019-0341-6.
64. Garst, E.H., Das, T., and Hang, H.C. (2021). Chemical approaches for investigating site-specific protein S-fatty acylation. Curr. Opin. Chem. Biol. *65*, 109–117. https://doi.org/10.1016/j.cbpa.2021.06.004.
65. Garst, E.H., Lee, H., Das, T., Bhattacharya, S., Percher, A., Wiewiora, R., Witte, I.P., Li, Y., Peng, T., Im, W., and Hang, H.C. (2021). Site-specific lipidation enhances IFITM3 membrane interactions and antiviral activity. ACS Chem. Biol. *16*, 844–856. https://doi.org/10.1021/acschembio.1c00013.
66. Choy, A., Dancourt, J., Mugo, B., O'Connor, T.J., Isberg, R.R., Melia, T.J., and Roy, C.R. (2012). The Legionella effector RavZ inhibits host autophagy through irreversible Atg8 deconjugation. Science *338*, 1072–1076. https://doi.org/10.1126/science.1227026.
67. Yang, A., Pantoom, S., and Wu, Y.W. (2017). Elucidation of the anti-autophagy mechanism of the Legionella effector RavZ using semisynthetic LC3 proteins. ELife *6*, e23905. https://doi.org/10.7554/eLife.23905.
68. Won, S.J., Cheung See Kit, M., and Martin, B.R. (2018). Protein depalmitoylases. Crit. Rev. Biochem. Mol. Biol. *53*, 83–98. https://doi.org/10.1080/10409238.2017.1409191.
69. Lin, D.T., and Conibear, E. (2015). Enzymatic protein depalmitoylation by acyl protein thioesterases. Biochem. Soc. Trans. *43*, 193–198. https://doi.org/10.1042/BST20140235.
70. Bononi, G., Tuccinardi, T., Rizzolio, F., and Granchi, C. (2021). α/β-hydrolase domain (ABHD) inhibitors as new potential therapeutic options against lipid-related diseases. J. Med. Chem. *64*, 9759–9785. https://doi.org/10.1021/acs.jmedchem.1c00624.
71. Li, L., Tong, M., Fu, Y., Chen, F., Zhang, S., Chen, H., Ma, X., Li, D., Liu, X., and Zhong, Q. (2021). Lipids and membrane-associated proteins in autophagy. Protein Cell *12*, 520–544. https://doi.org/10.1007/s13238-020-00793-9.
72. Blanc, M., David, F., Abrami, L., Migliozzi, D., Armand, F., Bürgi, J., and van der Goot, F.G. (2015). SwissPalm: protein palmitoylation database. F1000Res. *4*, 261. https://doi.org/10.12688/f1000research.6464.1.
73. Schultz, S.W., Agudo-Canalejo, J., Chino, H., Migliano, S.M., Saito, C., Koyama-Honda, I., Stenmark, H., Brech, A., Mizushima, N., Knorr, R.L., and May, A.I. (2021). Should I bend or should I grow: the mechanisms of droplet-mediated autophagosome formation. Autophagy *17*, 1046–1048. https://doi.org/10.1080/15548627.2021.1887548.
74. Morishita, H., and Mizushima, N. (2019). Diverse cellular roles of autophagy. Annu. Rev. Cell Dev. Biol. *35*, 453–475. https://doi.org/10.1146/annurev-cellbio-100818-125300.
75. Deng, Z., Purtell, K., Lachance, V., Wold, M.S., Chen, S., and Yue, Z. (2017). Autophagy receptors and neurodegenerative diseases. Trends Cell Biol. *27*, 491–504. https://doi.org/10.1016/j.tcb.2017.01.001.
76. Scrivo, A., Bourdenx, M., Pampliega, O., and Cuervo, A.M. (2018). Selective autophagy as a potential therapeutic target for neurodegenerative disorders. Lancet Neurol. *17*, 802–815. https://doi.org/10.1016/S1474-4422(18)30238-2.
77. Djajadikerta, A., Keshri, S., Pavel, M., Prestil, R., Ryan, L., and Rubinsztein, D.C. (2020). Autophagy induction as a therapeutic strategy for neurodegenerative diseases. J. Mol. Biol. *432*, 2799–2821. https://doi.org/10.1016/j.jmb.2019.12.035.
78. Rubinsztein, D.C., Codogno, P., and Levine, B. (2012). Autophagy modulation as a potential therapeutic target for diverse diseases. Nat. Rev. Drug Discov. *11*, 709–730. https://doi.org/10.1038/nrd3802.
79. Whitmarsh-Everiss, T., and Laraia, L. (2021). Small molecule probes for targeting autophagy. Nat. Chem. Biol. *17*, 653–664. https://doi.org/10.1038/s41589-021-00768-9.
80. He, S., Li, Q., Jiang, X., Lu, X., Feng, F., Qu, W., Chen, Y., and Sun, H. (2018). Design of small molecule autophagy modulators: a promising druggable strategy. J. Med. Chem. *61*, 4656–4687. https://doi.org/10.1021/acs.jmedchem.7b01019.
81. Takahashi, D., and Arimoto, H. (2021). Selective autophagy as the basis of autophagy-based degraders. Cell Chem. Biol. *28*, 1061–1071. https://doi.org/10.1016/j.chembiol.2021.05.006.
82. Virlogeux, A., Scaramuzzino, C., Lenoir, S., Carpentier, R., Louessard, M., Genoux, A., Lino, P., Hinckelmann, M.V., Perrier, A.L., Humbert, S., and Saudou, F. (2021). Increasing brain palmitoylation rescues behavior and neuropathology in Huntington disease mice. Sci. Adv. *7*, eabb0799. https://doi.org/10.1126/sciadv.abb0799.
83. Brigidi, G.S., and Bamji, S.X. (2013). Detection of protein palmitoylation in cultured hippocampal neurons by immunoprecipitation and acyl-biotin exchange (ABE). J. Vis. Exp.50031 https://doi.org/10.3791/50031.
84. Martin, B.R. (2013). Nonradioactive analysis of dynamic protein palmitoylation. Curr. Protoc. Protein Sci. *73*, 14.15.1–14.15.9. https://doi.org/10.1002/0471140864.ps1415s73.
85. Liu, Y., Zeng, R., Wang, R., Weng, Y., Wang, R., Zou, P., and Chen, P.R. (2021). Spatiotemporally resolved subcellular phosphoproteomics. Proc. Natl. Acad. Sci. USA *118*, e2025299118. https://doi.org/10.1073/pnas.2025299118.
86. García-Mata, R., Bebök, Z., Sorscher, E.J., and Sztul, E.S. (1999). Characterization and dynamics of aggresome formation by a cytosolic GFP-chimera. J. Cell Biol. *146*, 1239–1254. https://doi.org/10.1083/jcb.146.6.1239.
87. Matuszak, N., Muccioli, G.G., Labar, G., and Lambert, D.M. (2009). Synthesis and in vitro evaluation of N-substituted maleimide derivatives as selective monoglyceride lipase inhibitors. J. Med. Chem. *52*, 7410–7420. https://doi.org/10.1021/jm900461w.
88. Jiang, X., Wang, X., Ding, X., Du, M., Li, B., Weng, X., Zhang, J., Li, L., Tian, R., Zhu, Q., et al. (2020). FAM134B oligomerization drives endoplasmic reticulum membrane scission for ER-phagy. EMBO J. *39*, e102608. https://doi.org/10.15252/embj.2019102608.

STAR★METHODS

KEY RESOURCES TABLE

REAGENT or RESOURCE	SOURCE	IDENTIFIER
Antibodies		
DYKDDDDK-tag (Flag), Mouse	GenScript	Cat#A00187; RRID:AB_1720813
HA-tag (F-7)	Santa Cruz Biotechnology	Cat#sc-7392; RRID:AB_627809
Ubiquitin (P4D1)	Santa Cruz Biotechnology	Cat#sc-8017; RRID:AB_628423
GFP	ZENBIO	Cat#300943
Anti-p62 (SQSTM1) pAb	Medical Biological Laboratories	Cat#PM045; RRID:AB_1279301
Anti-multi ubiquitin mAb (FK2)	Medical Biological Laboratories	Cat#D058-3; RRID:AB_592937
LC3A/B	Cell Signaling Technology	Cat#12741S; RRID:AB_2617131
β-actin	Beyotime	Cat#AF0003; RRID:AB_2893353
Histone H3	Beyotime	Cat#AF0009; RRID:AB_2715593
Na^{+}/K^{+} ATPase	Beyotime	Cat#AF1864; RRID:AB_2857942
mCherry	abcam	Cat#ab167453; RRID:AB_2571870
GAPDH	ZENBIO	Cat#200306-7E4; RRID:AB_2722713
LYPLA1 (APT1)	ABclonal	Cat#A4419; RRID:AB_2765665
ATG7	Proteintech	Cat#10088-2-AP; RRID:AB_2062351
Alexa Fluor 488-labeled Goat Anti-Rabbit IgG (H+L)	Beyotime	Cat#A0423; RRID:AB_2891323
Alexa Fluor 647-labeled Goat Anti-Rabbit IgG (H+L)	Beyotime	Cat#A0468; RRID:AB_2936379
Alexa Fluor 488-labeled Goat Anti-Mouse IgG (H+L)	Beyotime	Cat#A0428; RRID:AB_2893435
Alexa Fluor 555-labeled Donkey Anti-Mouse IgG(H+L)	Beyotime	Cat#A0460; RRID:AB_2890133
Alexa Fluor 647-labeled Goat Anti-Mouse IgG (H+L)	Beyotime	Cat#A0473; RRID:AB_2891322
HRP-labeled Goat Anti-Rabbit IgG (H+L)	Beyotime	Cat#A0208; RRID:AB_2892644
HRP-labeled Goat Anti-Mouse IgG (H+L)	Beyotime	Cat#A0216; RRID:AB_2860575
HRP-conjugated Streptavidin	Beyotime	Cat#A0303
Chemicals, peptides, and recombinant proteins		
Dulbecco's modified eagle medium (DMEM)	Shanghai BasalMedia Technology	Cat#P110L7
Foetal bovine serum (FBS)	Biological Industries	Cat#04-001-1ACS
Dialyzed FBS	Biological Industries	Cat#04-011-1A
Opti-MEM™ reduced serum medium	Gibco	Cat#31985062
Lipofectamine™ 3000	Thermo Fisher	Cat#L3000015
Polyethylenimine, linear (PEI)	Polysciences, Inc.	Cat#23966-2
L-Ascorbic acid sodium salt	Solarbio	Cat#S9440
D-PBS	Solarbio	Cat#D1040
Mounting Medium (antifading)	Solarbio	Cat#S2100
D-Biotin	Solarbio	Cat#D8150
Azide-PEG3-Biotin conjugate	Jena Bioscience	Cat#CLK-AZ104P4-25
Palmostatin B	Merck	Cat#178501
Tris(3-hydroxypropyltriazolylmethyl)amine (THPTA)	Merck	Cat#762342
Torin 1	MedChemExpress	Cat#HY-13003
Puromycin	MedChemExpress	Cat#HY-B1743A
Protease inhibitor cocktail, mini-Tablet (EDTA-Free)	MedChemExpress	Cat#HY-K0011
Streptavidin-coated magnetic beads	MedChemExpress	Cat#HY-K0208
Protein A/G magnetic beads	MedChemExpress	Cat#HY-K0202
3× Flag-peptide TFA	MedChemExpress	Cat#HY-P0319A
Stearic acid alkyne	MedChemExpress	Cat#HY-101016
Palmitic acid alkyne	Iris Biotech	Cat#RL-2060

(Continued on next page)

Continued

REAGENT or RESOURCE	SOURCE	IDENTIFIER
Trypsin solution	Biosharp	Cat#BL512A
DAPI	Biosharp	Cat#BL105A
4% Paraformaldehyde	Biosharp	Cat#BL539A
Penicillin-streptomycin solution	Biosharp	Cat#BL505A
N-Ethylmaleimide (NEM)	Sangon Biotech	Cat#A600450
Biotin-BMCC	Sangon Biotech	Cat#C100222
Phenylmethylsulfonyl fluoride (PMSF)	MACKLIN	Cat#P6140
50% hydroxylamine (HAM) solution in H_2O	MACKLIN	Cat#H828371
N,*N*-Diisopropylethylamine (DIPEA)	MACKLIN	Cat#H810969
Triisopropylsilane (TIS)	MACKLIN	Cat#T819181
Trifluoroacetic acid (TFA)	MACKLIN	Cat#T818781
Acetonitrile (ACN)	MACKLIN	Cat#A801455
Maleimide	MACKLIN	Cat#M812744
Bafilomycin A1	Selleck	Cat#S1413
Vps34 IN1	Selleck	Cat#S7980
MG132	Selleck	Cat#S2619
LysoSensorTM Green DND-189	YEASEN	Cat#40767ES50
Propidium iodide (PI)	Beyotime	Cat#ST511
IPTG	Biotopped	Cat#Top1080S
Fatty acid free BSA V	Biotopped	Cat#B1009A
TCEP	Aladdin	Cat#T107252
Triphenylphosphine (PPh_3)	Aladdin	Cat#T104475
Anhydrous tetrahydrofuran (THF)	Aladdin	Cat#T103263
N,*N*-Dimethylformamide (DMF)	Aladdin	Cat#D112000
aminocaproic acid	Aladdin	Cat#F117709
Diethylazodicarboxylate	J&K Scientific	Cat#A10532896
Hexadecanol	Boer Inc	Cat#B603672
Neopentyl alcohol	HEOWNS	Cat#D-75730
5-FITC	GuoPing Pharmaceutical	Cat#GP010852-1
Rink Amide-MBHA resins	MACKLIN	Cat#R916084
O-Benzotriazole-*N*,*N*,*N*',*N*'-tetramethyl-uronium-hexafluorophosphate (HBTU)	Aladdin	Cat#H106174
1-Hydroxybenzotriazole (HOBt)	GL Biochem(Shanghai) Ltd.	Cat#00602
Methoxpolyethylene glycol maleimide (Maleimide-PEG)	Sigma	Cat#63187
Agarose	Sigma	Cat#A2576
Cholesterol	Sigma	Cat#C8667
1,2-dipalmitoyl-*sn*-glycero-3-phosphocholine (PC)	Avanti®	Cat#850355P
1-oleoyl-2-(12-biotinyl(aminododecanoyl))-*sn*-glycero-3-phosphoethanolamine (Biotin-PE)	Avanti®	Cat#860562
1,2-dipalmitoyl-*sn*-glycero-3-phosphoethanolamine-N-(lissamine rhodamine B sulfonyl) (rhodamine-PE)	Avanti®	Cat#810158
1-palmitoyl-2-oleoyl-*sn*-glycero-3-phospho-L-serine (POPS)	Avanti®	Cat#840034
Dichloromethane (DCM)	CHRON CHEMICALS	CAS#75-09-2
Diethyl ether	CHRON CHEMICALS	CAS#60-29-7
Piperidine	Sinopharm Chemical Reagent Co., Ltd	Cat#20201202
Restriction endonuclease EcoR I	Takara	Cat#1040S
Restriction endonuclease Hind III	Takara	Cat#1060A

(*Continued on next page*)

Continued		
REAGENT or RESOURCE	SOURCE	IDENTIFIER
Restriction endonuclease Xho I	Takara	Cat#1094S
Restriction endonuclease Sal I	Takara	Cat#1080S
Restriction endonuclease Nhe I	Takara	Cat#1241S
Restriction endonuclease Dpn I	Takara	Cat#1235A
T4 DNA ligase	Takara	Cat#2011A
FITC-EEKSSSQPSSCCSDPSKPGGNV	This paper	N/A
Palmitate-maleimide conjugated FITC-EEKSSSQPSSCCSDPSKPGGNV	This paper	N/A
mCherry-p62(155–440)^{C331S} recombinant protein	This paper	N/A
Palmitate-maleimide conjugated mCherry-p62(155–440)^{C331S} recombinant protein	This paper	N/A
GFP^{C48S}-p62(155–440)^{C331S} recombinant protein	This paper	N/A
palmitate-maleimide conjugated GFP^{C48S}-p62(155–440)^{C331S} recombinant protein	This paper	N/A
RavZ recombinant protein	This paper	N/A
**Critical commercial assays**		
Enhanced BCA protein assay kit	Beyotime	Cat#P0010
Trelief™ SoSoo cloning kit	Tsingke	Cat#TSV-S1
EndoFree Mini Plasmid kit II	TIANGEN	Cat#DP118-02
BlasTaq™ 2× PCR MasterMix	ABM Inc.	Cat#G895
BeyoECL Moon kit	Beyotime	Cat#P0018FS
Universal DNA purification kit	TIANGEN	Cat#4992197
**Deposited data**		
Raw data	This paper	Mendeley Data: http://dx.doi.org/10.17632/39c7x8ksmw.1
**Experimental models: Cell lines**		
HeLa cells	ATCC	N/A
NRK cells	Li Yu (Tsinghua University)	N/A
HEK293T cells	ATCC	N/A
MCF7 cells	ATCC	N/A
p62-KO NRK cells	Li Yu (Tsinghua University)	N/A
p62-KO NRK cells stably expressing GFP-p62	This paper	N/A
p62-KO NRK cells stably expressing GFP-p62^{C289,290S}	This paper	N/A
ZDHHC19-KO HEK293T cells	This paper	N/A
APT1-KO HEK293T cells	This paper	N/A
ATG7-KO HEK293T cells	Kefeng Lu (Sichuan University)	N/A
FIP200-KO HeLa cells	Min Li (Sun Yat-sen University)	N/A
FIP200-KO MEF cells	Yueguang Rong (Huazhong University of Science and Technology)	N/A
HEK293 cells stably expressing mCherry-LC3	This paper	N/A
HEK293T cells stably expressing GFP-ZDHHC19	This paper	N/A
MCF7 cells stably expressing GFP-LC3	This paper	N/A
**Experimental models: Organisms/strains**		
Escherichia coli DH5α	Tsingke	Cat#TSC-C14

(*Continued on next page*)

Continued

REAGENT or RESOURCE	SOURCE	IDENTIFIER
Escherichia coli BL21 Rosetta (DE3)	Tsingke	Cat#TSC-E01
Oligonucleotides		
Flag-p62_for_Hind III	CCCAAGCTTATGGCGTCG CTCACCGTGAAG	N/A
GFP-p62_rev_EcoR I	CCGGAATTCTCACAACGG CGGGGGATGCTT	N/A
GFP-p62_for_Xho I	CCGCTCGAGCTATGGCG TCGCTCACCGTG	N/A
p62^{C26S}_for	CTTCAGCTTCAGCTGCAG CCCCGAGCCTGAGGCGG AAGCCGA	N/A
p62^{C27S}_for	CAGCTTCTGCAGCAGCCC CGAGCCTGAGGCGGAAG CCGAGGC	N/A
p62^{D69A}_for	CAGGCGCACTACCGCGcTG AGGACGGGGACTTGGTT	N/A
p62^{C105S}_for	GAAAAAAGAGAGCCGGCGGG ACCACCGCCCACCGTGTGCTCA	N/A
p62^{C113S}_for	CCGCCCACCGAGTGCTCAGG AGGCGCCCCGCAACATGGTGCA	N/A
p62^{C289S}_for	GCCAAGCAGCAGCTGCTCTGA CCCCAGCAAGCCGGGTGGGAA	N/A
p62^{C290S}_for	AAGCAGCTGCAGCTCTGACCC CAGCAAGCCGGGTGGGAATGT	N/A
p62^{C289,290S}_for	AAGCAGCTGCAGCTCTGACCC CAGCAAGCCGGGTGGGAATGT	N/A
p62^{C331S}_for	GAGTCGGATAACTCTTCAGGAGGA	N/A
p62(283–296)_for	TCGAGCTAGCTCACAGCCAAGC AGCTGCTGCTCTGACCCCAGC AAGCCGTGAG	N/A
p62(283–296)_rev	AATTCTCACGGCTTGCTGGGGTC AGAGCAGCAGCTGCTTGGCTG TGAGCTAGC	N/A
p62(283–296)^{C289,290S}_for	TCGAGCTAGCTCACAGCCAAGCA GCAGCAGCTCTGACCCCAGCAA GCCGTGAG	N/A
p62(283–296)^{C289,290S}_rev	AATTCTCACGGCTTGCTGGGGTC AGAGCTGCTGCTGCTTGGCTGT GAGCTAGC	N/A
p62(279–300)_for	TCGAGCTGAGGAGAAGAGCAGCT CACAGCCAAGCAGCTGCTGCTCT GACCCCAGCAAGCCGGGTGGGA ATGTTTGAG	N/A
p62(279–300)_rev	AATTCTCAAACATTCCCACCCGG CTTGCTGGGGTCAGAGCAGCAG CTGCTTGGCTGTGAGCTGCTCT TCTCCTCAGC	N/A
p62(279–300)^{C289,290S}_for	TCGAGCTGAGGAGAAGAGCAGC TCACAGCCAAGCAGCAGCAGCT CTGACCCCAGCAAGCCGGGTG GGAATGTTTGAG	N/A
p62(279–300)^{C289,290S}_rev	AATTCTCAAACATTCCCACCCGG CTTGCTGGGGTCAGAGCTGCTGC TGCTTGGCTGTGAGCTGCTCTTC TCCTCAGC	N/A

(*Continued on next page*)

Continued

REAGENT or RESOURCE	SOURCE	IDENTIFIER
p62(285–294)_for	TCGAGCTCAGCCAAGCAGCTGC TGCTCTGACCCCAGCTGAG	N/A
p62(285–294)_rev	AATTCTCAGCTGGGGTCAGAGCA GCAGCTGCTTGGCTGAGC	N/A
p62(285–294)^{C289,290S}_for	TCGAGCTCAGCCAAGCAGCAGCA GCTCTGACCCCAGCTGAG	N/A
p62(285–294)^{C289S,290S}_rev	AATTCTCAGCTGGGGTCAGAGCTG CTGCTGCTTGGCTGAGC	N/A
SEB_p62_for_Hind III	CCCAAGCTTATGGCGTCGCTCACC	N/A
SEB_p62_rev_Sal I	ACGCgtcgacTCACAACGGCGGGGGATGCT	N/A
ZDHHC19^{C142S}_for	tcgaccaccatagcaagtgggtc	N/A
GFP^{C48S}_for	GTTCATCTCCACCACCGGCAAGCTG	N/A
APT1^{S119A}_for	attttgggagggtttgctcagggaggagctttatct	N/A
mCherry-p62(155–440)_for	GTACAAGACCATGGAGGGAAA GGGCTTGCAC	N/A
mCherry-p62(155–440)_rev	CTTTCCCTCCATGGTCTTGT ACAGCTCGTC	N/A
GFP_for	CTCTACTTCCAATCCCATATGGCA ATGGTGAGCAAGGGCGAG	N/A
GFP_rev	GTGCAAGCCCTTTCCCTCCATGG TCTTGTACAGCTCGTCCAT	N/A
GFP-p62(155–440)_for	ACCATGGAGGGAAAGGGCTT	N/A
GFP-p62(155–440)_rev	TGCCATATGGGATTGGAAGT	N/A
Flag-p62(124–440)_for	GATGACAAGCTTCCCAATGTGAT CTGCGATGGCTGCAATG	N/A
Flag-p62(124–440)_rev	AGATCACATTGGGAAGCTTGTC ATCGTCATCCT	N/A
HA-LC3_for_Nhe I	GTTTAGTGAACCGTCAGATCCGC TAGCTGTCGTGAGGAATTCTCT	N/A
HA-LC3_rev_Hind III	GAAGGTCTTCTCCGACGGCATAA GCTTGAATTCGATATCAGATCT	N/A
**Recombinant DNA**		
pEGFP-C1	Clontech	Cat#HG-VYC0084
pCMV-Flag-C1	This paper	N/A
pSEB-Flag-C1 vector	Zhenghong Lin (Chongqing Universuty)	N/A
pSEB-EGFP-C1 vector	This paper	N/A
pCMV-HA-C1 vector	This paper	N/A
pmCherry-C1	Clontech	Cat#632524
pCMV_Flag-p62	Jieqiong Tan (Central South University)	N/A
pCMV_Flag-p62^{C26S}	This paper	N/A
pCMV_Flag-p62^{C27S}	This paper	N/A
pCMV_Flag-p62^{C105S}	This paper	N/A
pCMV_Flag-p62^{C113S}	This paper	N/A
pCMV_Flag-p62^{C289S}	This paper	N/A
pCMV_Flag-p62^{C290S}	This paper	N/A
pCMV_Flag-p62^{C289,290S}	This paper	N/A
pCMV_GFP-p62	This paper	N/A
pSEB_GFP-p62	This paper	N/A
pSEB_GFP-p62^{C289,290S}	This paper	N/A
pEF_BOS-HA-ZDHHCs	Masaki Fukata (National Institute for Physiological Sciences)	N/A

(*Continued on next page*)

Continued

REAGENT or RESOURCE	SOURCE	IDENTIFIER
pCMV_HA-ZDHHC19	This paper	N/A
pCMV_HA-ZDHHC19^{C142S}	This paper	N/A
pEF_BOS-HA-ZDHHC19-Turbo	This paper	N/A
pCMV_HA-APT1	This paper	N/A
pCMV_HA-APT1^{S119A}	This paper	N/A
pCMV_HA-APT2	This paper	N/A
pCMV_HA-PPT1	This paper	N/A
pCMV_HA-PPT2	This paper	N/A
pCMV_HA-ABHD17a	This paper	N/A
pCMV_Flag-GFP-p62(285–294)	This paper	N/A
pCMV_Flag-GFP-p62(285–294)^{C289,290S}	This paper	N/A
pCMV_Flag-GFP-p62(283–296)	This paper	N/A
pCMV_Flag-GFP-p62(283–296)^{C289,290S}	This paper	N/A
pCMV_Flag-GFP-p62(279–300)	This paper	N/A
pCMV_Flag-GFP-p62(279–300)^{C289,290S}	This paper	N/A
pCMV_Flag-p62(124-440)	This paper	N/A
pCMV_Flag-p62(124-440)^{C289,290S}	This paper	N/A
pCMV_Flag-p62^{D69A}	This paper	N/A
pCMV_Flag-p62$^{D69A,\ C289,290S}$	This paper	N/A
pCMV_HA-LC3	This paper	N/A
pCMV_mCherry-LC3	This paper	N/A
pCMV_GFP-LC3	This paper	N/A
pHis-MBP-TEV-mCherry-p62	Li Yu (Tsinghua University)	N/A
pHis-MBP-TEV-GFP^{C48S}-p62(155–440)^{C331S}	This paper	N/A
pHis-MBP-TEV-mCherry-p62(155–440)^{C331S}	This paper	N/A
pDsRed2-ER	MIAOLING BIOLOGY	Cat#P0141
Software and algorithms		
LAS X	Leica	N/A
GraphPad Prism version 7.00	https://www.graphpad.com/scientific-software/prism/	N/A
ImageJ	https://fiji.sc/#cite	N/A
Image Lab	Bio-Rad	N/A
Xcalibur Software	Thermo Fisher Scientific	N/A
olyvia 3.3-24382	OLYMPUS	N/A

RESOURCE AVAILABILITY

Lead contact

Further information and requests for resources and reagents should be directed to and will be fulfilled by the lead contact, Aimin Yang (aimin.yang@cqu.edu.cn).

Materials availability

Plasmids and cell lines generated in this study will be available upon request.

Data and code availability

- Original western blot images and microscopy data have been deposited in the Mendeley dataset and are publicly available upon publication. The DOI is listed in the key resources table.
- This paper does not report original code.
- Any additional information required to reanalyze the data reported in this paper is available from the lead contact upon request.

EXPERIMENTAL MODEL AND STUDY PARTICIPANT DETAILS

E. coli strains and media

E. coli DH5α and BL21 Rosetta (DE3) strains were grown in Luria-Bertani (LB) medium.

Cell lines

NRK (ATCC, CRL-6509), HeLa, MCF7 (ATCC, HTB-22), HEK293 (ATCC, CRL-1573), HEK293T (ATCC, CRL-3216) and MEF (ATCC, SCRC-1040) cells were maintained in Dulbecco's modified Eagle's medium (DMEM) supplemented with 10% fetal bovine serum (FBS) and 1% penicillin-streptomycin solution in a humidified incubator at 37°C in 5% CO_2. Lipofectamine™ 3000 transfection reagent (Thermo Fisher, L3000015) or polyethylenimine (PEI) (Polysciences, Inc., 23966-2) was used for transfection of cells according to the manufacturer's instructions. The total DNA plasmid amounts used in this study were in the range of 0.5-2 μg per well in 6-well plates or 10-15 μg per 10 cm dish plated to 80% confluency. WT NRK cells and *p62*-KO NRK cells were a kind gift from Prof. Li Yu (Tsinghua University). *FIP200*-KO HeLa cells and *FIP200*-KO MEF cells were a kind gift from Prof. Min Li (Sun Yat-sen University) and Prof. Yueguang Rong (Huazhong University of Science and Technology), respectively. *ATG7*-KO HEK293T cells were a kind gift from Prof. Kefeng Lu (Sichuan University).

To delete *ZDHHC19* in HEK293T cells, two guide RNAs (gRNAs) against the 3rd exon in the human *ZDHHC19* genome were designed, using an online tool (http://crispor.tefor.net/). The forward gRNA sequence (ACGGGGCCTTCCGCCTGCAA) and the reverse gRNA sequence (CACACCACGTGCACCGTCAA) are against the upstream and downstream of exon 3, respectively. The double-stranded oligos for each gRNA were synthesized by Shanghai BiOligo Biotechnology and cloned into the pX458 vector (48138, Addgene). The plasmids were co-transfected into HEK293T cells by Lipofectamine™ 3000 (Thermo Fisher, L3000015). Single GFP-fluorescence cell was sorted by fluorescence-activated cell sorting (FACS) (BD Biosciences) to pick single-cell clones. KO cells were confirmed by genotyping and sequencing PCR-amplified genomic DNA. The pair of primers: forward-5'-CTTGTCCCCA CCCTTCCTTG-3' and reverse-5'-GACAGGTGCAGGTGGAGAAC-3' were used for genotyping. Genomic DNA was extracted using a universal DNA purification kit (TIANGEN, 4992197). Sequencing results showed that the design for CRISPR/Cas9 gene targeting was successful, and 56 bp in the *ZDHHC19* genome was missing, causing premature termination of the protein.

To delete *APT1* in HEK293T cells, two guide RNAs (gRNAs) against the 7th exon in the human *APT1* genome were designed, using an online tool (http://crispor.tefor.net/). The forward gRNA sequence (GTGTCACTGCACTCAGTTGC) and the reverse gRNA sequence (CAGTTTCTGCTGTGTGGTAA) are against the upstream and downstream of exon 7 respectively. The double-stranded oligos for each gRNA were synthesized by Shanghai BiOligo Biotechnology and cloned into the pX458 vector (48138, Addgene). The plasmids were co-transfected into HEK293T cells by Lipofectamine™ 3000 (Thermo Fisher, L3000015). Single GFP-fluorescence cell was sorted by fluorescence-activated cell sorting (FACS) (BD Biosciences) to pick single-cell clones. KO cells were confirmed by genotyping and sequencing PCR-amplified genomic DNA. The pair of primers: forward-5'-TGGGCAAGCATG TGTGTTC-3' and reverse-5'-ACCTTCTGCTCAGTTTTGCT-3' were used for genotyping. Genomic DNA was extracted using a universal DNA purification kit (TIANGEN, 4992197). Sequencing results showed that the design for CRISPR/Cas9 gene targeting was successful, and 38 bp in the *APT1* genome was missing, causing premature termination of the protein.

METHOD DETAILS

Plasmid constructions and cloning

Human p62 cDNA was subcloned into the pEGFP-C1 vector, pCMV-Flag-C1 vector and pSEB-EGFP-C1 vector. Human p62 (residues 124–440) cDNA was subcloned into the pCMV-Flag-C1 vector, and p62 (residues 155–440) was subcloned into the His6-MBP-TEV-mCherry vector and His6-MBP-TEV-GFP vector. Mouse ZDHHC19 cDNA was subcloned into the pLJM1-GFP-C1 vector and pCMV-HA-C1 vector. Human APT1 cDNA was subcloned into the pCMV-HA-C1 vector. Human LC3B cDNA was subcloned into the pmCherry-C1 vector, pGFP-C1 vector and pCMV-HA-C1 vector. All PCRs were performed using BlasTaq™ 2× PCR MasterMix (ABM Inc., G895) and *Trelief*™ SoSoo Cloning Kit (Tsingke Biotechnology, TSV-S1) according to the manufacturer's instructions. The Flag- or HA- epitope was introduced by PCR-driven overlap extension. Mutants for p62, ZDHHC19 and APT1 were generated using site-directed mutagenesis system with single-chain primers. All plasmids were verified by DNA sequencing.

Western blotting

Cells incubated in 6-well culture dishes were rinsed in cold phosphate buffered saline (PBS) and lysed in 100 μL of Lysis Buffer (50 mM Tris-HCl pH 7.4, 150 mM NaCl, 1% NP-40, 10% glycerol, 1 mM $MgCl_2$) supplemented with freshly prepared inhibitor cocktail (MedChemExpress, HY-K0011). Protein was quantified by an Enhanced BCA protein assay kit (Beyotime, P0010). Then, 5× Sample Buffer (250 mM Tris-HCl pH 6.8, 10% SDS, 0.25% bromophenol blue, 50% glycerol, 5% β-mercaptoethanol) was added, and the samples were boiled at 95°C for 5 min. Ten micrograms of protein from each sample was loaded onto 8–12.5% gels, separated by SDS-PAGE, and then transferred to PVDF membranes (Millipore, IPVH00010). The membranes were blocked with 5% non-fat powdered milk (Sangon Biotech, A600669) (HRP-conjugated streptavidin, blocked with 5% BSA (Beyotime, ST023)), and incubated overnight with primary antibodies (anti-Flag, mouse, dilution 1:5000; anti-Flag, rabbit, dilution 1:3000; anti-HA, dilution 1:1000; anti-p62, dilution 1:5000; anti-LC3A/B, dilution 1:1000; anti-APT1, dilution 1:1000; anti-β-actin, dilution 1:1000; anti-GAPDH, dilution

1:8000; anti-ATG7, dilution 1:1000; anti-Ubiquitin, dilution 1:1000; anti-FK2, dilution 1:5000; anti-GFP, dilution 1:8000; anti-mCherry, dilution 1:1000; anti-Na^+/K^+ ATPase, dilution 1:10000; anti-Histone H3, dilution 1:8000). After incubation, the membranes were washed with TBST (20 mM Tris-HCl, 150 mM NaCl, 0.05% Tween 20), incubated with secondary antibodies (HRP-labeled goat anti-rabbit, dilution 1:5000; HRP-labeled goat anti-mouse, dilution 1:5000 and HRP-conjugated streptavidin, dilution 1:5000) and visualized by a Western blotting imager (GelDoc XR, Bio-Rad).

Acyl-biotin exchange (ABE) assay

The ABE assay was performed as previously described with minor modifications.[36,83] In brief, cells transfected with Flag-p62 were harvested 48 h after transfection and washed with cold PBS. Prior to cell lysis, 50 mM *N*-ethylmaleimide (NEM; Sangon Biotech, A600450) dissolved in EtOH was freshly added to the Lysis Buffer (50 mM Tris-HCl pH 7.5, 150 mM NaCl, 1 mM $MgCl_2$, 1% NP-40, 10% glycerol) with inhibitor cocktail. Cells were then suspended in NEM-containing Lysis Buffer for 1.5 h at 4°C and centrifugated at 15,000 rpm for 30 min at 4°C. The supernatants were incubated with both anti-Flag antibody (GenScript, A00187) and protein A/G magnetic beads (MedChemExpress, HY-K0202) at 4°C overnight. For *S*-acylation detection of endogenous p62, anti-p62 antibody (MBL, PM045) was used to enrich endogenous p62. After incubation, the beads were washed five times with Lysis Buffer of pH 7.5 and then three times with Lysis Buffer of pH 7.2. For the hydroxylamine (HAM; MACKLIN, H828371) treatment, beads were equivalently divided into two tubes (+HAM and -HAM). Then, freshly prepared HAM-containing Lysis Buffer (50 mM Tris-HCl pH 7.2, 150 mM NaCl, 1 mM $MgCl_2$, 1% NP-40, 10% glycerol, 0.75 M HAM and inhibitor cocktail) was added to the +HAM tube and incubated at room temperature for 1 h. As a control, the Lysis Buffer of pH 7.2 was added to the -HAM tube. The beads were then washed four times with Lysis Buffer of pH 7.2 and three times with Lysis Buffer of pH 6.2. Subsequently, beads were treated with 5 μM biotin-BMCC (Sangon Biotech, C1002225) in Lysis Buffer of pH 6.2 freshly prepared at 4°C for 1 h. The immunoprecipitated samples were analyzed by Western blotting using HRP-conjugated streptavidin (Beyotime, A0303) and anti-Flag antibody (GenScript, A00187).

LC−MS of HAM-mediated hydrolysate

MCF7 cells were harvested and incubated with 50 mM NEM. The supernatants were incubated with both anti-p62 antibody (MBL, PM045) and protein A/G magnetic beads (MedChemExpress, HY-K0202) at 4°C for overnight. After incubation, the beads were washed five times with Lysis Buffer (50 mM Tris-HCl pH 7.5, 150 mM NaCl, 1 mM $MgCl_2$, 1% NP-40, 10% glycerol, and inhibitor cocktail) and then three times with Lysis Buffer of pH 7.2. Then, freshly prepared HAM-containing Lysis Buffer of pH 7.2 was added and incubated at room temperature for 1 h. As a control, Lysis Buffer of pH 7.2 with inhibitor cocktail was added. After HAM treatment, the supernatant was collected and subjected to Q Exactive Plus Orbitrap LC−MS (Thermo Fisher). The sample was separated using C18 (3 μm, 75 nm × 15 cm) (Thermo Fisher) at a flow rate of 600 nL/min. The gradient was set as follows: 3% B (0.1% formic acid in ACN)/97% A (0.1% formic acid in H_2O) to 8% B for 5 min, 8% B to 20% B for 38 min, 20% B to 30% B for 8 min, 30% B to 90% B for 2 min and 90% B for 7 min. The data were collected in the negative ion and positive ion modes, and stearic and palmitic acids were analyzed in the negative ion mode.

Metabolic labeling and click chemistry

Metabolic labeling of bioorthogonal chemical reporters of protein acylation and detection of protein *S*-acylation via click chemistry were performed as previously described with minor modifications.[84] Prior to labeling, the cells were incubated with DMEM containing 5% dialyzed FBS (BI, 04-011-1A) for 1 h. 100 μM of the chemical reporter, palmitic acid alkyne (Alk-C16; Iris Biotech, RL-2060) or stearic acid alkyne (Alk-C18; MedChemExpress, HY-101016) (50 mM stock in DMSO) was pre-incubated with 10% fatty acid free BSA (Biotopped, B1009A) and added to DMEM supplemented with 5% dialyzed FBS (BI, 04-011-1A). After labeling overnight, cells were washed with cold D-PBS (Solario, D1040) and resuspended in Lysis Buffer (50 mM Tris-HCl, pH 7.5, 150 mM NaCl, 1% NP-40, 0.5% sodium deoxycholate) supplemented with inhibitor cocktail, PMSF (MACKLIN, P6140) and 1 μM Palmostatin B (Merck, 178501) for 1.5 h on ice. The lysates were centrifuged at 15,000 rpm for 30 min at 4°C, and the supernatants were incubated with both anti-Flag antibody (GenScript, A00187) and protein A/G magnetic beads (MedChemExpress, HY-K0202) at 4°C overnight. The beads were then washed with Click Buffer (50 mM Tris-HCl, pH 7.5, 150 mM NaCl, 1% NP-40) and incubated in Click Buffer supplemented with 100 μM Azide-PEG3-biotin (Jena Bioscience, CLK-AZ104P4-25), 1 mM L-Ascorbic acid sodium salt (S9440), 1 mM $CuSO_4$, and 0.5 mM THPTA (100× stock solution: 50 mM THPTA in deionized water) for 1.5 h. The immunoprecipitated samples were then analyzed by Western blotting using HRP-conjugated streptavidin (Beyotime, A0303) and anti-Flag antibody (GenScript, A00187).

Proximity labeling

Proximity labeling was performed as previously described with minor modifications.[54,85] HEK293T cells were grown in DMEM (10% FBS) at 37°C under 5% CO_2 and transfected with the ZDHHC19-TurboID plasmid by Lipofectamine™ 3000 (Thermo Fisher, L3000015). After 24 h, the cells were labeled by 500 μM D-Biotin (Solarbio, D8150) for 40 min at 37°C with fresh DMEM. The cells were then washed four times with cold PBS and collected with cold PBS at 800 g for 5 min. The cell lysates were extracted with RIPA Buffer (50 mM Tris-HCl pH 7.4, 150 mM NaCl, 0.5% sodium deoxycholate and 1% Triton X-100) for 30 min with rotation at 4°C and centrifuged at 12,000 rpm for 10 min at 4°C. Next, proteins were precipitated by adding CH_3OH:$CHCl_3$:H_2O at a ratio of 4:1:3, and washed three times with ice-cold CH_3OH. After air-drying, 2% SDS/PBS buffer was added for protein redissolving at room temperature for 1 h, followed by diluting with PBS to 0.1% SDS/PBS. Then, streptavidin-coated magnetic beads

(MedChemExpress, HY-K0208) were incubated with the protein sample for 2 h with rotation at room temperature. The beads were washed once with 0.2% SDS/PBS buffer, three times with PBS buffer and once with ddH_2O, followed by transferring to a new 1.5 mL tube. 2× SDS Sample Buffer with 2 mM D-Biotin and 20 mM DTT was added to the beads for 95°C boiling for 10 min to elute purified proteins for Western blotting analysis.

Immunofluorescence

Cells were grown on glass coverslips (CITOTEST, 12-545-100) followed by fixation using 4% paraformaldehyde (Biosharp, BL539A) for 15 min at room temperature. The fixed cells were rinsed three times with PBS, permeabilized with 0.5% Triton X-100 for 30 min (for HEK293T cells, permeabilized for 10 min) and blocked with 10% FBS in PBS for 1 h at room temperature (RT), followed by incubation with primary antibody (anti-p62 antibody, dilution 1:500 (MBL, PM045); anti-LC3A/B antibody, dilution 1:100 (CST, 12741S); anti-Ub antibody (FK2), dilution 1:100 (MBL, D058-3); anti-HA antibody, dilution 1:100 (Santa Cruz, sc-7392)) diluted in PBS overnight at 4°C. Then, the cells were rinsed three times with PBS containing 0.01% Tween 20 and incubated with secondary antibody (Alexa Fluor 488-labeled goat anti-rabbit IgG (H+L), Alexa Fluor 647-labeled goat anti-rabbit IgG (H+L), Alexa Fluor 488-labeled goat anti-mouse IgG (H+L) or Alexa Fluor 647-labeled goat anti-mouse IgG (H+L), dilution 1:500). For nucleus staining, cells were incubated with DAPI (Biosharp, BL105A) in room temperature for 5 min, and were then rinsed three times with PBS containing 0.01% Tween 20.

Microscopy

Paraformaldehyde-fixed NRK, HEK293, and HEK293T cells were visualized using a Leica TCS SP8 DIVE confocal microscope (Leica) equipped with an Airyscan detector and a 63× oil immersion objective (1.4 numerical aperture; Leica). Images were acquired in Airyscan mode, and processed with LAS X software (Leica). As indicated, samples were probed with anti-p62 (MBL, PM045; dilution 1:500), anti-HA (Santa Cruz, F-7; dilution 1:100), anti-FK2 (MBL, D058-3; dilution 1:500) or anti-LC3A/B antibody (CST, 12741S; dilution 1:100). Imaging conditions and intensity scales were similar for each group presented together.

Biochemical fractionation

Protein aggregates were determined by fractioning cell lysates in Triton X-100 soluble and insoluble fractions as previously described.[86] Briefly, cells were rinsed with cold PBS, lysed in PBS containing 2% Triton X-100 supplemented with inhibitor cocktail, and incubated on ice for 1 h. The lysates were centrifuged at 15000 rpm for 40 min at 4°C. The supernatant was recovered as the soluble fraction. Pellets were rinsed with PBS three times and then resuspended in 1% SDS supplemented with inhibitor cocktail and sonicated to obtain the insoluble fraction. Protein was quantified by an Enhanced BCA protein assay kit (Beyotime, P0010) and then determined by Western blotting.

Immunoprecipitation analysis

Cells transfected with HA-ZDHHC19 and Flag-p62, GFP-p62 and Flag-p62, or Flag-p62 and HA-LC3 were lysed by Lysis Buffer (50 mM Tris-HCl pH 7.5, 150 mM NaCl, 1% NP-40) with inhibitor cocktail. The supernatants were incubated with both antibody and protein A/G magnetic beads (MedChemExpress, HY-K0202) at 4°C for overnight. Subsequently, the beads were washed with cold Lysis Buffer five times. The immunoprecipitated samples were quantified by an Enhanced BCA protein assay kit (Beyotime, P0010) and analyzed by Western blotting using anti-Flag and anti-HA antibodies.

For immunoprecipitation analysis of protein-palmitate conjugate and ubiquitin, HEK293T cells were lysed by Lysis Buffer (50 mM Tris-HCl pH 7.4, 150 mM NaCl, 1% NP-40) with inhibitor cocktail. The supernatants were harvested by centrifugation at 15,000 rpm, 20 min, 4°C and were mixed with 20 ng/μL GFP-p62 protein or palmitate-maleimide conjugated GFP-p62 protein. The mixture was then incubated with both anti-GFP antibody (ZENBIO, 300943) and protein A/G magnetic beads (MedChemExpress, HY-K0202) at 4°C for overnight. Subsequently, the beads were washed with cold Lysis Buffer for five times and eluted with SDS Sample Buffer. Ubiquitin and p62 were analyzed by western blotting using anti-Ub antibody (Santa Cruz, sc-8017) and anti-GFP antibody (ZENBIO, 300943), respectively.

Protein expression and purification

Plasmid containing p62 or RavZ was transformed into *E. coli* BL21 Rosetta (DE3) cells. Protein expression was induced with 0.2–0.4 mM IPTG and carried out at 18°C overnight. Purification was performed with the ÄKTA™ prime plus chromatography purification system (GE Healthcare Life Sciences). The cells were collected and re-suspended in Lysis Buffer (40 mM Tris-HCl pH 7.4, 150 mM NaCl, 10% glycerol, 1 mM EDTA, 1 mM PMSF) for sonication. All of the proteins used in this study contain tags (GFP, mCherry or MBP) with an extra N-terminal His6 tag. The proteins were purified by Ni-NTA affinity purification using HisTrap HP column (GE Healthcare Life Sciences) eluted with a gradient of 4–70% of 500 mM imidazole. Purified proteins were collected and concentrated by Centrifugal Filtration (30 K NMWL) (Merck Millipore, UFC803096). The concentration of target protein was measured by SDS-PAGE using BSA standard samples.

To obtain p62 proteins in mammalian cells, the plasmid encoding Flag-p62 WT or Flag-p62^{C289,290S} was transfected in *p62*-KO NRK cells for 48 h. The cells were then treated with 10 μM MG132 (Selleck, S2619) for 6 h and resuspended in Lysis Buffer (50 mM Tris pH 7.4, 150 mM NaCl, 1% NP-40, 10% glycerol, cocktail inhibitor). The Flag-p62 WT or Flag-p62^{C289,290S} protein was purified by anti-Flag antibody (GenScript, A00187) and protein A/G magnetic beads (MedChemExpress, HY-K0202). The

Flag-p62 enriched beads were washed with PBS for five times and incubated with 50 μL 5 mM 3× Flag-peptide TFA (MCE, HY-P0319A) to elute Flag-p62 WT or Flag-p62^{C289,290S}. In the final step, the Flag-p62 WT or Flag-p62^{C289,290S} solution was diluted by 50 μL PBS.

Peptide synthesis and purification

All peptides were synthesized based on manual Fmoc-SPPS chemistry. Briefly, Rink Amide-MBHA resins (MACKLIN, R916084) with a loading capacity of 0.36 mmol/g were first swelled by DCM. For each coupling procedure, 4-fold excess of protected amino acid, HBTU, HOBt, and DIPEA (with a ratio of 1:1:1:2) in DMF was added to the resin for 90 minutes with shaking at room temperature. The deprotection reaction of the Fmoc group was performed in 20% piperidine in DMF (v/v) after the resins were washed with DMF 5 times. The procedure for the coupling of fluorescein 5-isothiocyanate (FITC; GuoPing Pharmaceutical, GP010852-1) was performed with 3-fold excess of 5-FITC and DIPEA (with a ratio of 1: 2) in DMF overnight. After completion of the synthesis, peptides were released from the resin by treatment with TFA/H_2O/TIS (95/2.5/2.5) for 2 h at room temperature. The crude peptides were precipitated by the addition of cold diethyl ether (Et_2O) and centrifugation.

For purification, the crude peptides were injected to RP-HPLC (Hitachi, Japan) equipped with a C18 column (Bondysil C18, 5 μm, 250 mm × 5.6 mm). 0.1% TFA in ACN (v/v) and 0.1% TFA in H_2O (v/v) were used as the mobile phase A and B, respectively. For all the analytical HPLC trials, the total flow rate was set to be 1 mL/min, and the A concentration raised from 10 % to 100 % over 21 min following a linear gradient. For the purification of peptides in a larger scale by semi-prep HPLC columns (Bondysil C18, 5 μm, 250 mm × 10 mm), the total flow rate was set to be 3 mL/min and the A concentration raised from 10% to 100% over 21 min following a linear gradient. The peptide peaks were collected, lyophilized, and confirmed by MALDI-TOF mass spectrometry analysis (SHIMADZU Corporation, Japan).

Synthesis of palmitate-maleimide

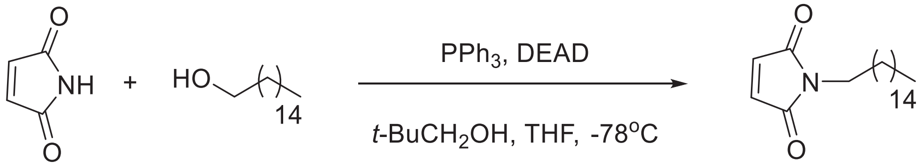

Scheme 1. Synthesis route of palmitate-maleimide

Palmitate-maleimide was prepared according to the previous reported literature (Scheme 1).[87] To a 100 mL of round-bottom flask was added triphenylphosphine (PPh_3; 1.35 g, 5.2 mmol) and 50 mL of anhydrous tetrahydrofuran (THF). The resulting clear solution was cooled to -78°C. Diethyl azodicarboxylate (DEAD; 0.82 mL, 5.2 mmol) was added over a period of 2–3 min. The yellow mixture was then stirred during 5 min, after which hexadecanol (1.33 g, 5.7 mmol) was added over 1 min and stirred for 5 min. Then neopentyl alcohol (*t*-$BuCH_2OH$; 0.25 g, 2.8 mmol) and maleimide (0.50 g, 5.2 mmol) were added. The resulting suspension was allowed to remain at -78°C for 5 min during which time most of the maleimide dissolved. The cooling bath was then removed, and the mixture was stirred overnight at room temperature. After completion of the reaction, the clear solution was evaporated and the resulting solid was purified by a column chromatography.^{1}H NMR (600 MHz, $CDCl_3$) δ 6.67 (s, 2H), 3.5 (t, J = 7.2 Hz, 2H), 1.56 (m, 2H), 1.25 (s, 26H), 0.87 (t, J = 6.7 Hz, 3H).

Preparation of protein/peptide conjugates

p62 protein conjugation with palmitate-maleimide was carried out in buffer containing 6.75 μM p62 protein, 2 mM palmitate-maleimide, 50 mM Tris-HCl pH7.4, 150 mM NaCl, 1 mM TCEP (Aladdin, T107252), 1% Triton X-100 for 2 h at 20°C. After reaction, the conjugation efficiency was measured by adding 5 mM methoxpolyethylene glycol maleimide (Mal-PEG, 5 kD; Sigma, 63187) for an additional reaction and analyzed by SDS-PAGE. The conjugated proteins were purified through a Ni-NTA beads in 150 mM NaCl, 10 mM Tris-HCl, pH 8.0, 1 mM EDTA, 0.1% NP-40.

The peptide conjugation with palmitate-maleimide was performed by mixing 2 mM peptide, 5 mM palmitate-maleimide, 1 mM TCEP (Aladdin, T107252) in DMSO for 1.5 h at 25°C. After reaction, the conjugated peptides were purified by semi-prep HPLC columns (Bondysil C18, 5 μm, 250 mm × 10 mm). The purified peptides were collected, lyophilized, and confirmed by MALDI-TOF mass spectrometry analysis (SHIMADZU Corporation, Japan).

GUV assay

GUV assay was performed as previously described with minor modifications.[88] 1% agarose was used to coat one glass slide for overnight, while the other glass slide was coated by 200 μL streptavidin solution (7 μg streptavidin dissolved in 200 μL ddH_2O) and dry for overnight. For preparation of GUVs, 40 μL of chloroform was added to 60 μL lipids solution (15 μL 10 mM DPPC, 30 μL 10 mM cholesterol, 12.5 μL 10 mM Biotin-PE, 1 μL 10 mM rhodamine-PE and 1.5 μL 10 mM POPS). The lipids solution was then transferred to the agarose-coated glass slide by glass syringe (HEMILTON) and dry with Argon gas for 3 h. Then, 200 μL PBS was gently added to

agarose glass for 10 min to suspend the GUVs, which were subsequently added to the streptavidin-coated glass slide. After 20 min incubation, GUVs were anchored to glass slides through biotin-streptavidin interaction. p62 protein or peptide was injected into the chamber. The fluorescence of GUVs was observed continuously for 60 min using a laser scanning confocal microscope (Olympus IX2). For each representative images, three different experiments were performed to ensure the reproducibility.

Preparation of membrane fraction

To prepare total cellular membrane fraction preparation, HEK293T or *p62*-KO NRK were scraped and resuspended in PBS. Then cells were frozen by liquid nitrogen and thawed at 37°C for five cycles. The cell nucleus was removed by centrifugation at 1,000 g, 5 min, 4°C. Membrane fraction was achieved by centrifugation at 15,000 rpm, 20 min, 4°C.

To prepare total LC3-positive or LC3-negative cellular membrane, the HEK293T or *ATG7*-KO HEK293T cells were treated with 1 μM Torin 1 (MedChemExpress, HY-13003) and 1 μM Baf-A1 (Selleck, S1413) for 4 h. The cells were scraped and resuspended in PBS. Then cells were frozen by liquid nitrogen and thawed at 37°C for five cycles. The cell nucleus was removed by centrifugation at 1,000 g, 5 min, 4°C. Membrane fraction was achieved by centrifugation at 15,000 rpm, 20 min, 4°C and washed with PBS.

Alternatively, to prepare total LC3-positive or LC3-negative cellular membrane, the GFP-LC3-expressing MCF7 cells were treated with 1 μM Torin 1 (MedChemExpress, HY-13003) and 1 μM Baf-A1 (Selleck, S1413) for 4 h. The cells were scraped and resuspended in PBS. Then cells were frozen by liquid nitrogen and thawed at 37°C for five cycles. The cell nucleus was removed by centrifugation at 1,000 g, 5 min, 4°C. The LC3-positive membrane was obtained by centrifugation at 15,000 rpm, 20 min, 4°C and washed with PBS. To prepare LC3-negative membrane, the LC3-positive membrane was resuspended in Reaction Buffer (50 mM Tris pH 7.5, 150 mM NaCl, 1mM DTT) and incubated with 50 ng/μL MBP-RavZ protein for 1 h at 37°C. The LC3-negative membranes were achieved by centrifugation at 15,000 rpm, 20 min, 4°C and washed with PBS.

Co-precipitation assay

For co-precipitation assay of protein-palmitate conjugate and total membrane, 20 ng/μL p62 protein or palmitate-maleimide conjugated p62 protein was added into 200 μL total cellular membrane and incubated for indicated time at room temperature, of which 100 μL mixture was transferred to new tube as control (Total). The membrane-binding p62 was separated by centrifugation at 15,000 rpm, 20 min, 4°C, and washed with PBS. The supernatant was transferred to new tube (Supernatant). All samples were resuspended in SDS Sample Buffer and analyzed by Western blotting.

For co-precipitation assay of exogenously expressed Flag-p62 and membrane fraction, the total cellular membrane was resuspended in 100 μL Flag-p62 WT or Flag-p62^{C289,290S} solution for 1 h at room temperature, of which 50 μL mixture was transferred to new tube as control (Total). The membrane-binding p62 was separated by centrifugation at 15,000 rpm, 20 min, 4°C, and washed with PBS. The supernatant was transferred to new tube. All samples were resuspended in SDS Sample Buffer and analyzed by Western blotting.

FRAP analysis

The *p62*-KO NRK cells stably expressing GFP-p62 WT or GFP-p62^{C289,290S} were incubated in Lab-Tek II Chambered Coverglass (Thermo Scientific, 155382) at 37°C with 5% CO_2. The cells were analyzed by FRAP using an oil immersion objective (63×). GFP-p62 puncta were bleached for 13.08 s (30 × 0.436 s) with an 80% laser intensity of 488 nm. Recovery was recorded by fluorescence intensity per 10 s.

Non-reduced SDS-PAGE

The cells were lysed, and dissolved in SDS Sample Buffer (50 mM Tris, pH 7.5, 150 mM NaCl, 10% glycerol, 2% SDS, 0.1% bromophenol blue) with cocktail inhibitor. To retain the disulfide bonds, reducing agent is not permitted. Samples were boiled at 95°C for 5 min. Oligomer and monomer of p62 were separated by SDS-PAGE (4% stacking gel and 10% separating gel).

Cell death measurement

Freshly trypsinized cells (1×10^6 cells) were washed twice with cold PBS and resuspended in 195 μL Binding Buffer (Beyotime, C1065S). Cells were treated with 5 μL propidium iodide (PI, 30 μg/mL) (Beyotime, ST511) and incubated for 15 min at room temperature. After reaction, Cells were transferred to FACS tube, and 800 μL Binding Buffer was added. Then cells were analyzed by the Becton-Dickinson (BD) LSRFortessa™ flow cytometer. Data was analyzed by FlowJo 7.6.1 software.

QUANTIFICATION AND STATISTICAL ANALYSIS

Unless otherwise indicated, images are representative of cells from at least three separate experiments. Data presented in text and graphs are the means plus standard error of the mean (SEM) and are representative of at least three independent experiments. Unpaired *t* tests were used to establish the significance of experimentally observed differences.

Molecular Cell, Volume *83*

Supplemental information

S-acylation of p62 promotes p62 droplet recruitment into autophagosomes in mammalian autophagy

Xue Huang, Jia Yao, Lu Liu, Jing Chen, Ligang Mei, Jingjing Huangfu, Dong Luo, Xinyi Wang, Changhai Lin, Xiaorong Chen, Yi Yang, Sheng Ouyang, Fujing Wei, Zhuolin Wang, Shaolin Zhang, Tingxiu Xiang, Dante Neculai, Qiming Sun, Eryan Kong, Edward W. Tate, and Aimin Yang

Supplemental Information

S-acylation of p62 promotes p62 droplet recruitment into autophagosomes in mammalian autophagy

Xue Huang, Jia Yao, Lu Liu, Jing Chen, Ligang Mei, Jingjing Huangfu, Dong Luo, Xinyi Wang, Changhai Lin, Xiaorong Chen, Yi Yang, Sheng Ouyang, Fujing Wei, Zhuolin Wang, Shaolin Zhang, Tingxiu Xiang, Dante Neculai, Qiming Sun, Eryan Kong, Edward W. Tate, and Aimin Yang

This file includes:

Supplementary Figures and Legends

Figure S1 to S7

Supplemental Figures

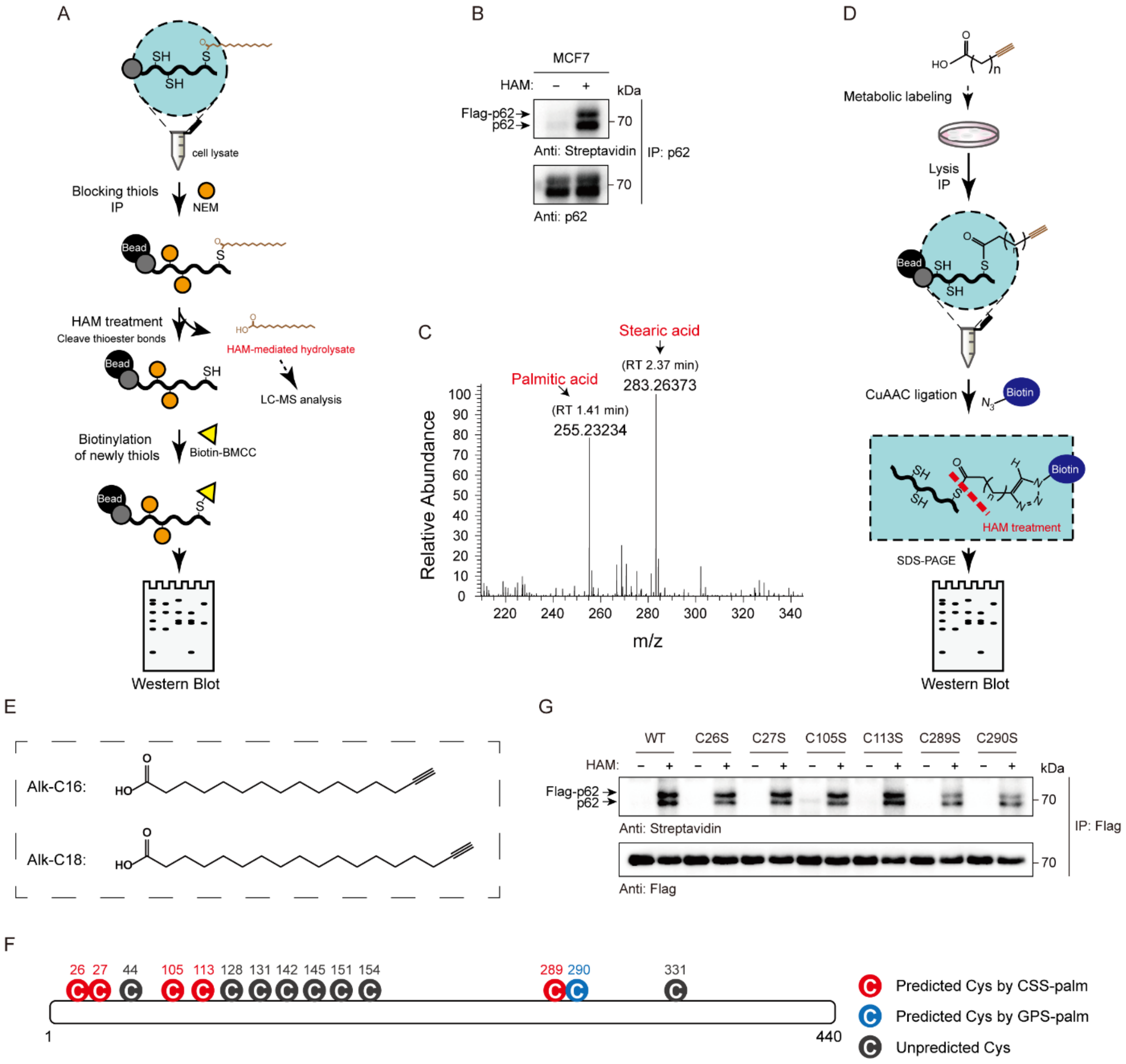

Figure S1. p62 undergoes *S*-acylation at Cys289 and Cys290, related to Figure 1

(**A**) Scheme of the acyl-biotin exchange (ABE) assay. Cell lysates are treated with *N*-ethylmaleimide (NEM) to irreversibly block free thiol (–SH) groups, and a target protein is then purified using immunoprecipitation. The purified target protein is subjected to treatment with Hydroxylamine (HAM) to expose the thiol groups of acylated cysteines and then reacts with a thiol-reactive biotin molecule, biotin-BMCC, resulting in specific biotinylation of the acylated cysteines.

(**B**) *S*-acylation of Flag-tagged p62 and endogenous p62 detected by ABE assay in MCF7 cells. Cells were transfected with the plasmid encoding Flag-tagged p62. Flag-tagged p62 and endogenous p62 were immunoprecipitated with anti-p62 antibody, and *S*-acylated p62 was further detected by the ABE assay.

(**C**) Mass spectrometry analysis of HAM-mediated hydrolysates. The HAM-mediated hydrolysates from MCF7 cells as in (**B**) were analyzed by mass spectrometry. Stearic acid and palmitic acid were detected in the HAM-mediated hydrolysates in negative ion mode. HRMS: stearic acid, *m/z*: calcd for $C_{18}H_{35}O_2$ 283.26371 $[M-H]^-$, found 283.26373; palmitic acid, *m/z*: calcd for $C_{16}H_{31}O_2$ 255.23241 $[M-H]^-$, found 255.23234.

(**D**) Scheme of chemical reporters for protein acylation and the click reaction. Cells are metabolically labeled with alkyne-functionalized lipid analogs. The on-beads copper (I)-catalyzed azide alkyne cycloaddition (CuAAC) click reaction is conducted with azide-tagged biotin (Azide-PEG3-Biotin Conjugate). HAM treatment hydrolyzes the thioester bonds, resulting in loss of biotinylation.

(**E**) Structure of Alk-C16 and Alk-C18. Alk-C16, palmitic acid alkyne. Alk-C18, stearic acid alkyne.

(**F**) Prediction of p62 acylation sites using prediction algorithms.

(**G**) *S*-acylation of Flag-p62 mutants in MCF7 cells detected by the ABE assay. Because of p62 oligomerization, anti-Flag antibody precipitated exogenous Flag-p62 (the upper band) as well as p62 (the lower band).

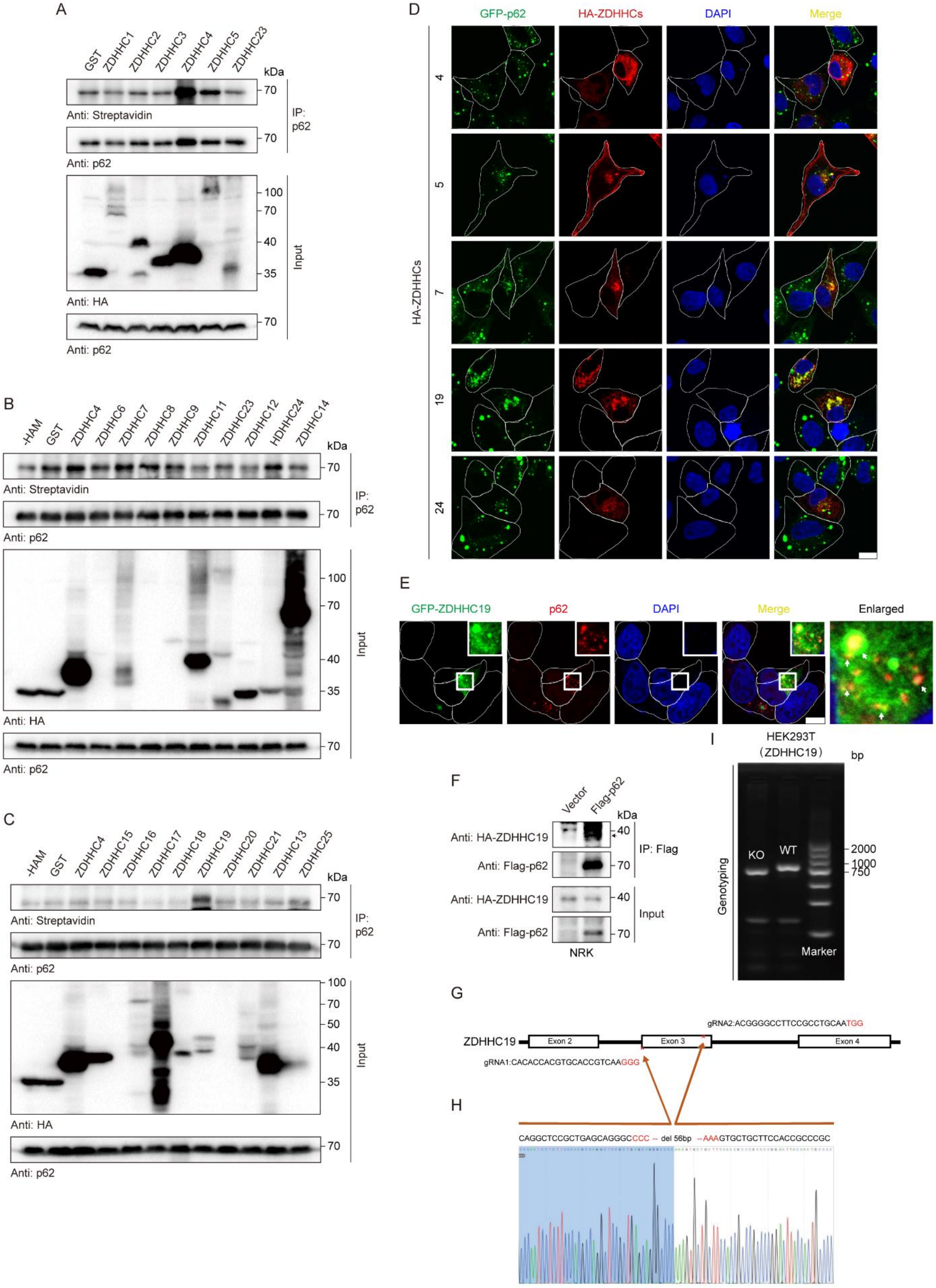
A
GST
ZDHHC1
ZDHHC2
ZDHHC3
ZDHHC4
ZDHHC5
ZDHHC23
kDa
70
Anti: Streptavidin
IP: p62
Anti: p62
100
40
35
Input
Anti: HA
Anti: p62
B
-HAM
GST
ZDHHC4
ZDHHC6
ZDHHC7
ZDHHC8
ZDHHC9
ZDHHC11
ZDHHC23
ZDHHC12
HDHHC24
ZDHHC14
C
ZDHHC15
ZDHHC16
ZDHHC17
ZDHHC18
ZDHHC19
ZDHHC20
ZDHHC21
ZDHHC13
ZDHHC25
50
D
GFP-p62
HA-ZDHHCs
DAPI
Merge
HA-ZDHHCs
4
5
7
19
24
E
GFP-ZDHHC19
p62
Enlarged
F
Vector
Flag-p62
Anti: HA-ZDHHC19
Anti: Flag-p62
IP: Flag
NRK
I
HEK293T
（ZDHHC19）
bp
KO
WT
2000
1000
750
Genotyping
Marker
G
gRNA2:ACGGGGCCTTCCGCCTGCAATGG
ZDHHC19
Exon 2
Exon 3
Exon 4
gRNA1:CACACCACGTGCACCGTCAAGGG
H
CAGGCTCCGCTGAGCAGGGCCCC -- del 56bp -- AAAGTGCTGCTTCCACCGCCCGC

Figure S2. ZDHHC19 mediates p62 *S*-acylation, related to Figure 2

(**A**), (**B**) and (**C**) p62 *S*-acylation in cells expressing ZDHHCs. MCF7 cells were transfected with HA-ZDHHC1-25 plasmids to screen for acyltransferases that could increase p62 acylation. Endogenous p62 was immunoprecipitated with anti-p62 antibody, and *S*-acylated p62 was further analyzed by ABE assay. Key ZDHHC candidates were replicated to examine the acylation level.

(**D**) Representative images of the colocalization of p62 with ZDHHCs. *p62*-KO NRK cells stably expressing GFP-p62 (green) were transfected with HA-ZDHHC4, 5, 7, 19 or 24 and immunostained with anti-HA antibody (red). Scale bar, 10 μm.

(**E**) Representative images of the colocalization of stable ZDHHC19 and endogenous p62. HEK293T cells stably expressing GFP-ZDHHC19 (green) were immunostained with anti-p62 (red) antibody. Scale bar, 10 μm.

(**F)** Immunoblot analysis of Flag-p62-immunoprecipitated (IP) proteins in NRK cells co-expressing Flag-p62 with HA-ZDHHC19.

(**G**) Deleting *ZDHHC19* in HEK293T cells. Targeting scheme of the truncation of exon 3 in human *ZDHHC19*. Two sgRNAs were designed to target the enzyme activity center (ACGGGGCCTTCCGCCTGCAA, CACACCACGTGCACCGTCAA) of human *ZDHHC19*.

(**H**) Sequencing verification of the *ZDHHC19*-KO cell line. The knockout allele has a deletion of 56 bp on exon 3, confirmed by sequencing, and the ORF finder predicts that the knockout allele results in a premature termination of the protein.

(**I**) Electrophoresis verification of the *ZDHHC19*-KO cell line. Genotyping was carried out by PCR amplification with corresponding primers and the amplicons of WT and knockout alleles were 420 bp and 364 bp, respectively.

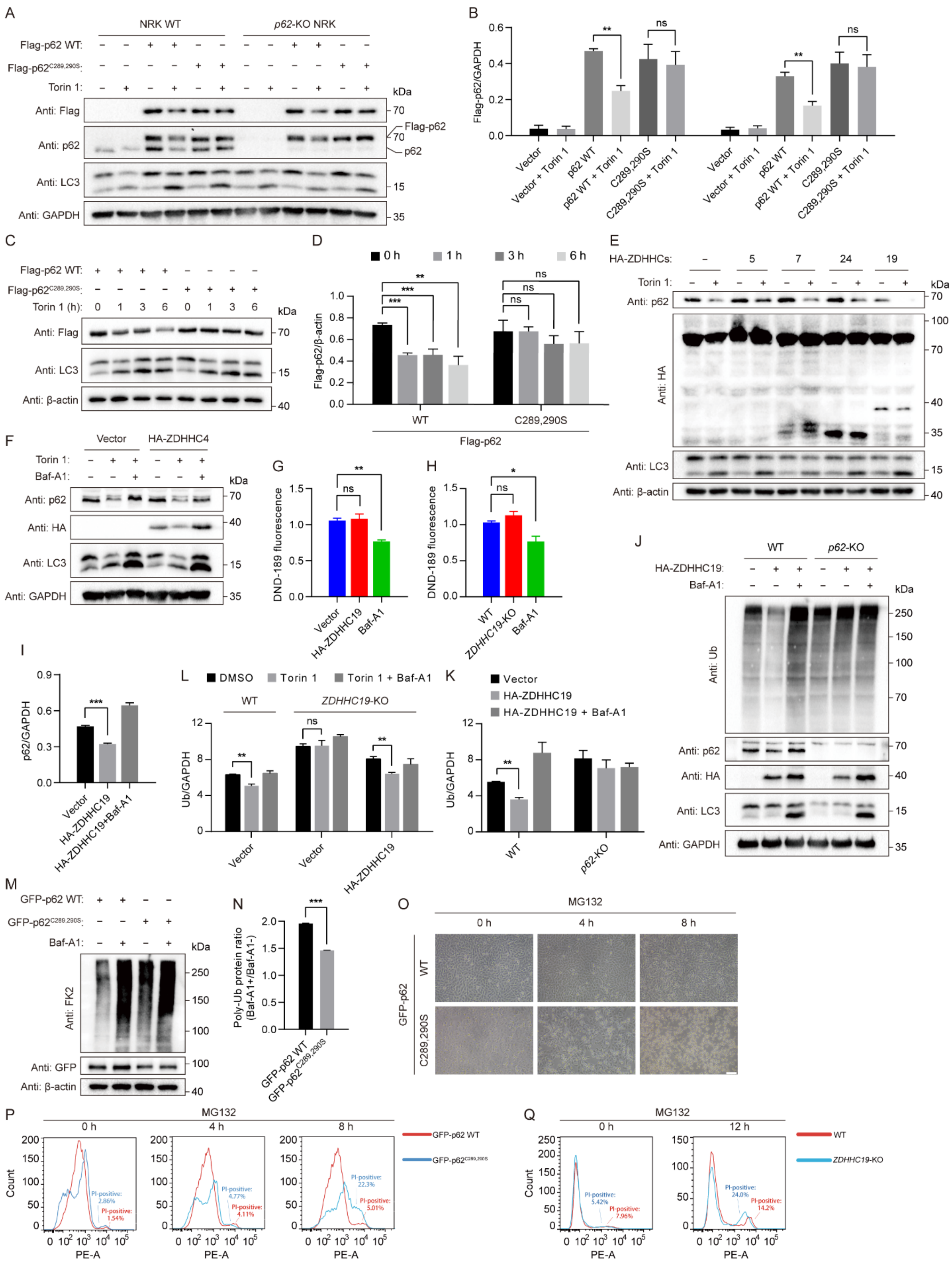
A
NRK WT
p62-KO NRK
Flag-p62 WT:
Flag-p62C289,290S:
Torin 1:
kDa
Anti: Flag
Anti: p62
Flag-p62
p62
Anti: LC3
Anti: GAPDH
B
Flag-p62/GAPDH
Vector
Vector + Torin 1
p62 WT
p62 WT + Torin 1
C289,290S
C289,290S + Torin 1
C
Torin 1 (h):
Anti: β-actin
D
0 h
1 h
3 h
6 h
Flag-p62/β-actin
WT
C289,290S
Flag-p62
E
HA-ZDHHCs:
Anti: HA
F
Vector
HA-ZDHHC4
Baf-A1:
G
DND-189 fluorescence
HA-ZDHHC19
Baf-A1
H
ZDHHC19-KO
I
p62/GAPDH
HA-ZDHHC19+Baf-A1
J
p62-KO
HA-ZDHHC19:
Anti: Ub
L
DMSO
Torin 1
Torin 1 + Baf-A1
Ub/GAPDH
K
HA-ZDHHC19 + Baf-A1
M
GFP-p62 WT:
GFP-p62C289,290S:
Anti: FK2
Anti: GFP
N
Poly-Ub protein ratio (Baf-A1+/Baf-A1-)
GFP-p62 WT
GFP-p62C289,290S
O
MG132
0 h
4 h
8 h
GFP-p62
P
Count
PE-A
PI-positive: 2.86%
PI-positive: 1.54%
PI-positive: 4.77%
PI-positive: 4.11%
PI-positive: 22.3%
PI-positive: 5.01%
Q
12 h
PI-positive: 5.42%
PI-positive: 7.96%
PI-positive: 24.0%
PI-positive: 14.2%

Figure S3. *S*-acylation facilitates the degradation of p62 and ubiquitinated proteins, related to Figure 3

(**A**) Immunoblot analysis of Flag-p62 WT and Flag-p62^{C289,290S} proteins in NRK WT or *p62*-KO cells. Cells were transiently transfected with Flag-p62 WT or Flag-p62^{C289,290S} under basal condition or upon induction of autophagy with the mTOR inhibitor Torin 1 (1 μM) for 1 h. Total proteins were extracted and detected by Western blotting.

(**B**) Quantification of Flag-p62 WT and Flag-p62^{C289,290S} protein levels as in (**A**). Data represent the mean ± SEM of three independent experiments. "ns", no significant difference, $^{**}P < 0.01$, Student's *t* test.

(**C**) Immunoblot analysis of Flag-p62 WT and Flag-p62^{C289,290S} upon autophagy induction. *p62*-KO NRK cells were transfected with Flag-p62 WT or Flag-p62^{C289,290S}, followed by Torin 1 (1 μM) treatment for the indicated time points.

(**D**) Quantification of Flag-p62 WT and Flag-p62^{C289,290S} protein levels as in (**C**). Data represent the mean ± SEM of three independent experiments. "ns", no significant difference, $^{**}P < 0.01$, $^{***}P < 0.001$, Student's *t* test.

(**E**) Immunoblot analysis of p62 protein level upon the expression of each ZDHHC. NRK cells were transfected with HA-ZDHHC5, 7, 24 or 19 and treated with DMSO or Torin 1 (1 μM) for 1 h.

(**F**) Immunoblot analysis of p62 protein level upon ZDHHC4 expression. NRK cells were transfected with empty vector or HA-ZDHHC4, and treated with Torin 1 (1 μM) for 1 h with or without Baf-A1 (1 μM). Baf-A1, Bafilomycin A1.

(**G**) Analysis of lysosomal pH in HEK293T cells expressing HA-ZDHHC19. The HEK293T cells transiently expressing empty vector or HA-ZDHHC19 were incubated with DND-189 (1 μM) for 1 h. Baf-A1 was used as positive control. Mean intensity of fluorescence was detected by flow cytometry and assessed by FlowJo software. Data represent the mean ± SEM of three independent experiments. "ns", no significant difference, $^{**}P < 0.01$, Student's *t* test.

(**H**) Analysis of lysosomal pH in WT or *ZDHHC19*-KO HEK293T cells. The WT or *ZDHHC19*-KO HEK293T cells were incubated with DND-189 (1 μM) for 1 h. Data represent the mean ± SEM of three independent experiments. "ns", no significant difference, $^{*}P < 0.05$, Student's *t* test.

(**I**) Quantification of p62 protein levels as in **Figure 3K**. Data represent the mean ± SEM of three independent experiments. ****P* < 0.001, Student's *t* test.

(**J**) Immunoblot analysis of total ubiquitinated protein and p62 protein level upon ZDHHC19 expression. WT or *p62*-KO NRK cells were transiently transfected with HA-ZDHHC19 and treated with DMSO or Baf-A1 (1 μM) for 6 h.

(**K**) Quantification of total ubiquitinated proteins as in (**J**). Data represent the mean ± SEM of three independent experiments. ***P* < 0.01, Student's *t* test.

(**L**) Quantification of total ubiquitinated protein levels as in **Figure 3E**. Data represent the mean ± SEM of three independent experiments. "ns", no significant difference, ***P* < 0.01, Student's *t* test.

(**M**) Immunoblot analysis of total poly-ubiquitinated proteins in GFP-p62 WT and GFP-$p62^{C289,290S}$ cells. *p62*-KO NRK cells stably expressing GFP-p62 WT or GFP-$p62^{C289,290S}$ were treated with DMSO or Baf-A1 (1 μM) for 18 h. The levels of polyubiquitinated proteins were detected with anti-FK2 antibody.

(**N**) Quantification of total ubiquitinated proteins in Baf-A1-treated cells to that in Baf-A1-untreated cells as in (**M**). Data represent the mean ± SEM of three independent experiments. ****P* < 0.001, Student's *t* test.

(**O**) Representative bright-field images of *p62*-KO NRK cells stably expressing GFP-p62 WT or GFP-$p62^{C289,290S}$. Cells were treated with MG132 (5 μM) for the indicated time points and observed by microscopy. Scale bar, 100 μm.

(**P**) Cell death assay of GFP-p62 WT or GFP-$p62^{C289,290S}$ cells. *p62*-KO NRK cells stably expressing GFP-p62 WT or GFP-$p62^{C289,290S}$ were treated with MG132 (5 μM) for the indicated time points and stained with propidium iodide (PI). The fluorescence of PI-stained cells was detected by flow cytometry and assessed by FlowJo software.

(**Q**) Cell death assay of WT or *ZDHHC19*-KO HEK293T cells. The WT or *ZDHHC19*-KO HEK293T cells were treated with MG132 (10 μM) for 8 h and stained with propidium iodide (PI).

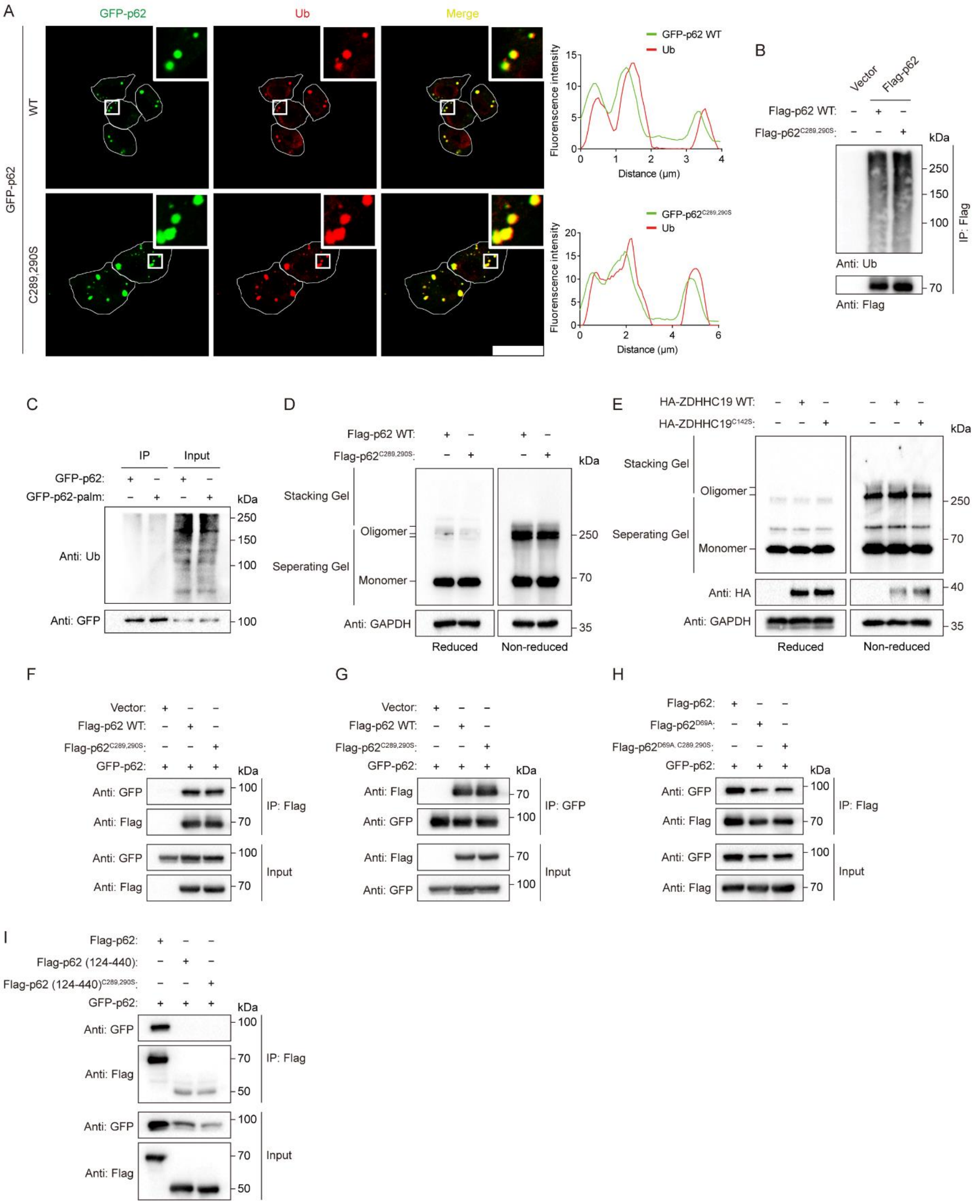

Figure S4. Effect of *S*-acylation on p62-ubiquitin binding and its oligomerization, related to Figure 4

(**A**) Representative images of colocalization of GFP-p62 and poly-ubiquitinated proteins. *p62*-KO NRK cells stably expressing GFP-p62 WT and GFP-p62^{C289,290S} (green) were immunostained with anti-FK2 antibody (red), and observed under confocal microscopy. The colocalization of GFP-p62 and poly-Ub was evaluated by ImageJ software. Scale bar, 10 μm.

(**B**) Immunoblot analysis of Flag-p62-immunoprecipitated (IP) ubiquitinated proteins in NRK cells. *p62*-KO NRK cells were transfected with empty vector (control), Flag-p62 WT or Flag-p62^{C289,290S}. Immunoprecipitation was performed with anti-Flag antibody and further analyzed by Western blotting.

(**C**) The pull-down assay of purified GFP-p62 and total ubiquitin proteins. The GFP-p62 or GFP-p62-palm was incubated with *p62*-KO NRK cell lysates for overnight. The ubiquitin was co-immunoprecipitated with anti-GFP antibody.

(**D**) Immunoblot analysis of Flag-p62 oligomerization. *p62*-KO NRK cells were transfected with Flag-p62 WT or Flag-p62^{C289,290S}. The oligomer and monomer were separated by non-reduced SDS-PAGE.

(**E**) Immunoblot analysis of p62 oligomerization upon ZDHHC19 expression. HEK293T cells were transfected with empty vector, HA-ZDHHC19 WT or HA-ZDHHC19^{C142S}. The oligomer and monomer were separated by non-reduced SDS-PAGE.

(**F**) Immunoblot analysis of Flag-p62-immunoprecipitated (IP) proteins in HEK293T cells co-expressing Flag-p62 with GFP-p62. The HEK293T cells were co-transfected with GFP-p62 and Flag-p62 (WT or the mutant p62^{C289,290S}). The empty vector was transfected as control.

(**G**) Immunoblot analysis of GFP-p62-immunoprecipitated (IP) proteins in HEK293T cells co-expressing GFP-p62 with Flag-p62. The HEK293T cells were co-transfected with GFP-p62 and Flag-p62 (WT or the mutant p62^{C289,290S}). The empty vector was transfected as control.

(**H**) Immunoblot analysis of Flag-p62 and its mutants-immunoprecipitated (IP) proteins in HEK293T cells co-expressing GFP-p62 with Flag-p62^{D69A} or Flag-p62$^{D69A, C289,290S}$. The Flag-p62 was transfected as control.

(**I**) Immunoblot analysis of Flag-p62 and its mutants-immunoprecipitated (IP) proteins in HEK293T cells co-expressing GFP-p62 with Flag-p62 (124–440) or Flag-p62 (124–440)^{C289,290S}. The Flag-p62 was transfected as control.

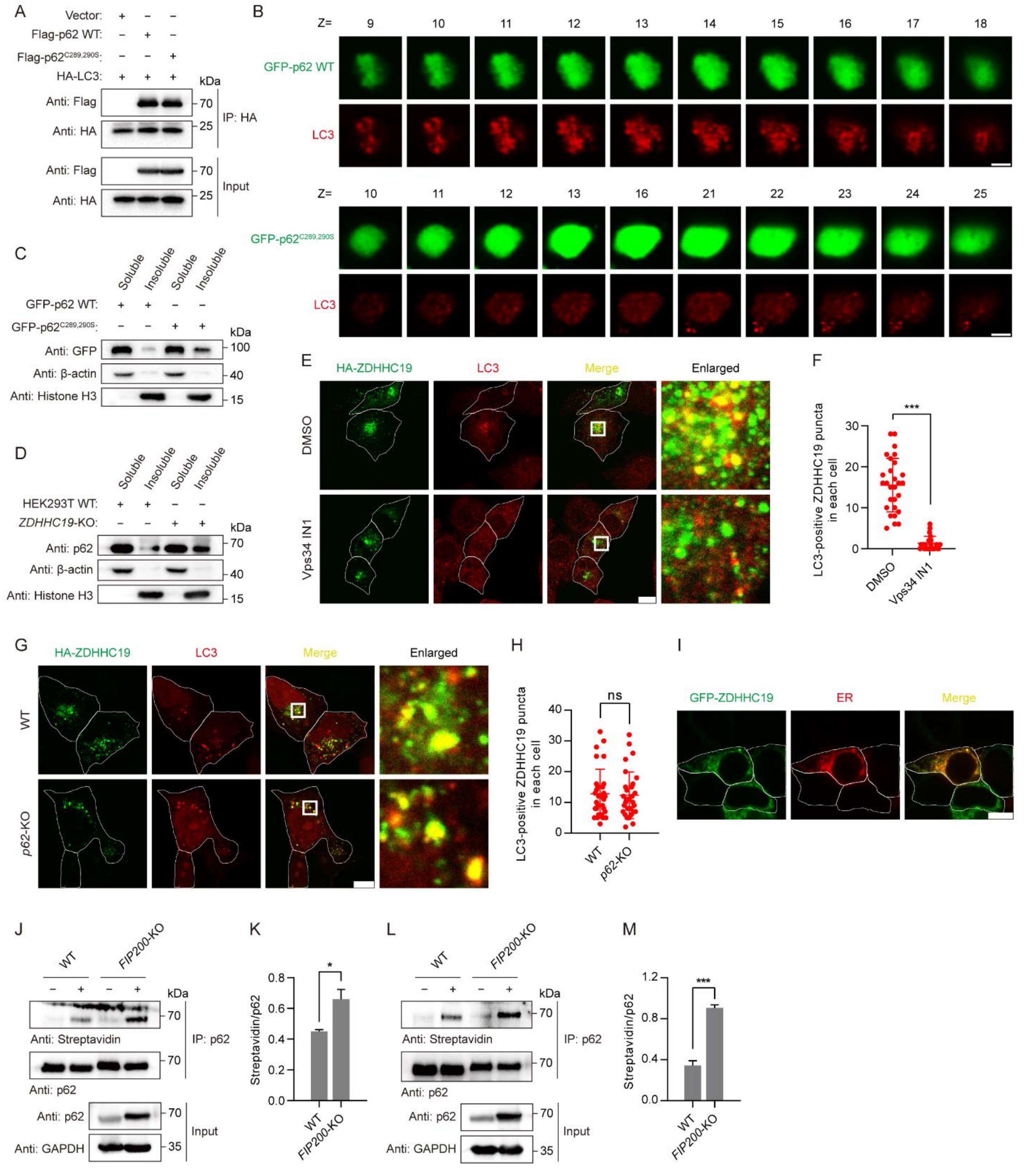

Figure S5. *S*-acylation regulates autophagic membrane localization of p62 droplets, related to Figure 5

(**A**) Immunoblot analysis of HA-LC3-immunoprecipitated (IP) proteins in HEK293T cells co-expressing HA-LC3 with Flag-p62 (WT or the mutant $p62^{C289,290S}$). The empty vector was transfected as control. The HA-LC3 was purified with anti-HA antibody.

(**B**) Representative images of LC3-positive p62 puncta in GFP-p62 WT or GFP-$p62^{C289,290S}$ cells upon HA-ZDHHC19 expression. *p62*-KO NRK cells stably expressing GFP-p62 WT or GFP-$p62^{C289,290S}$ were transiently transfected with HA-ZDHHC19. The p62- and LC3-positive puncta were analyzed by serial layer scanning. For GFP-p62 WT puncta, N=25 layers. For GFP-$p62^{C289,290S}$ puncta, N=30 layers. Interval= 0.2 μm. Scale bar, 0.5 μm.

(**C**) Analysis of Triton X-100 soluble and insoluble fractions of GFP-p62. *p62*-KO NRK cells stably expressing GFP-p62 WT or GFP-$p62^{C289,290S}$ were separated into Triton X-100 soluble and insoluble fractions.

(**D**) Analysis of Triton X-100 soluble and insoluble fractions of p62 in *ZDHHC19*-KO HEK293T cells.

(**E**) Representative images of the colocalization of ZDHHC19 and LC3 upon Vps34 IN1 treatment. HEK293T cells were transfected with HA-ZDHHC19, and treated with Vps34 IN1 (5 μM) for 6 h. ZDHHC19 and LC3 were immunostained with anti-HA antibody (green) and anti-LC3A/B antibody (red), respectively. Cells were visualized under confocal microscopy. Scale bar, 10 μm.

(**F**) Quantification of LC3-positive ZDHHC19 puncta in HEK293T cells as in (**E**). $^{***}P < 0.001$, Student's *t* test.

(**G**) Representative images of the colocalization of ZDHHC19 and LC3 in WT or *p62*-KO NRK cells. WT or *p62*-KO NRK cells were transfected with HA-ZDHHC19. ZDHHC19 and LC3 were immunostained with anti-HA antibody (green) and anti-LC3A/B antibody (red), respectively. Scale bar, 10 μm.

(**H**) Quantification of LC3-positive ZDHHC19 puncta in WT or *p62*-KO NRK cells as in (**G**). "ns", no significant difference, Student's *t* test.

(**I**) Representative images of the colocalization of stable ZDHHC19 and ER. The GFP-ZDHHC19 (green) stably expressing HEK293T cells were transiently transfected with DsRed2-ER (red). Scale bar, 10 μm.

(**J**) *S*-acylation of p62 in WT or *FIP200*-KO MEF cells. Endogenous p62 were immunoprecipitated with anti-p62 antibody, and *S*-acylated p62 was further detected by ABE assay.

(**K**) Quantification of p62 *S*-acylation in WT or *FIP200*-KO MEF cells as in (**J**). Data represent the mean ± SEM of three independent experiments. *$P < 0.05$, Student's *t* test.

(**L**) *S*-acylation of p62 in WT or *FIP200*-KO HeLa cells. The acylated p62 was detected by ABE assay.

(**M**) Quantification of p62 *S*-acylation in WT or *FIP200*-KO HeLa cells as in (**L**). Data represent the mean ± SEM of three independent experiments. ***$P < 0.001$, Student's *t* test.

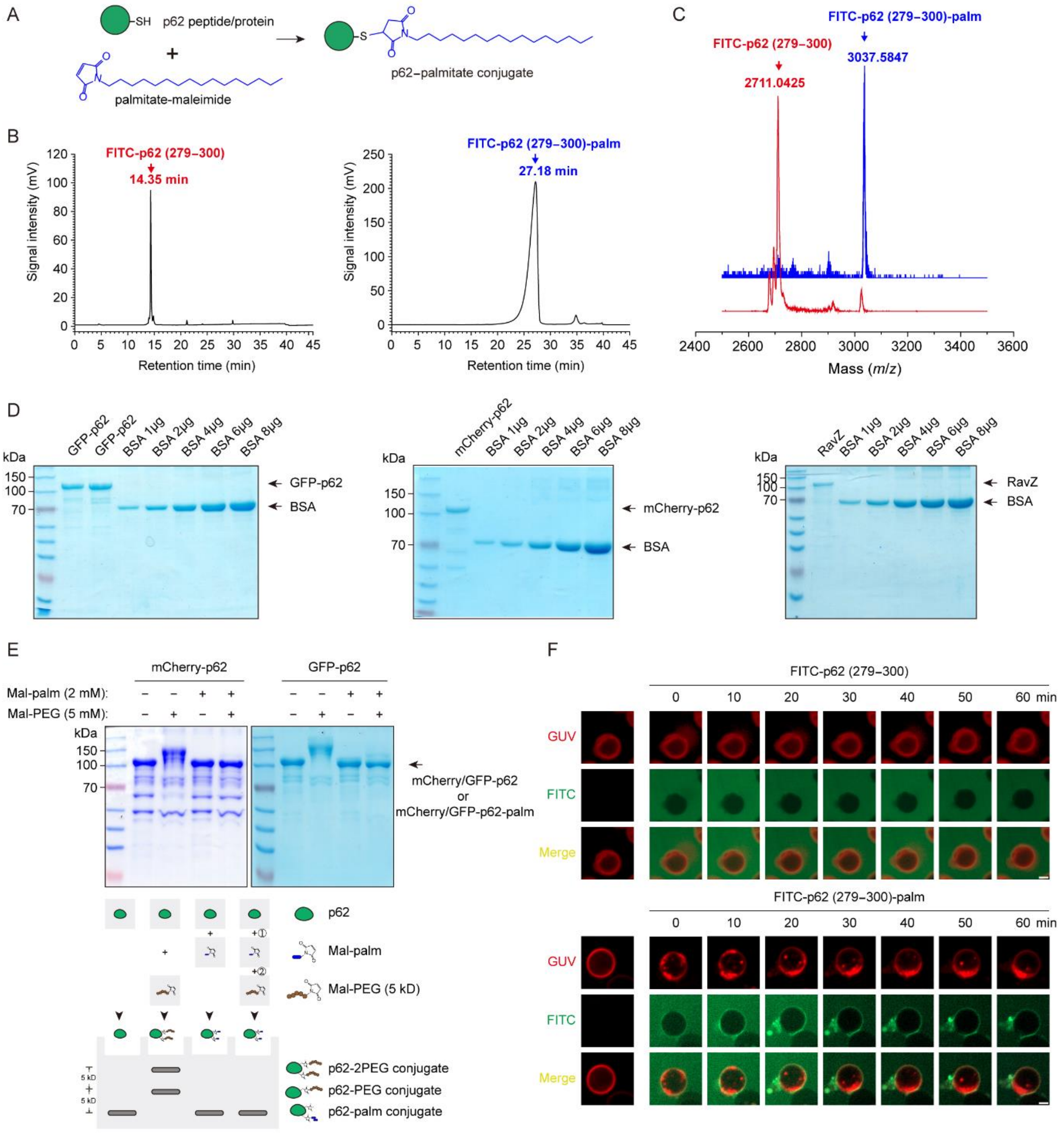

Figure S6. *S*-acylation enhances the affinity of p62 for LC3-positive membranes *in vitro*, related to Figure 6

(**A**) Schematic diagram of peptide/protein-palmitate conjugate. The palmitate-maleimide was conjugated to purified peptide or protein through the free thiol groups (–SH) of peptide or protein.

(**B**) HPLC analysis of FITC-p62 (279–300) peptide (left panel) and palmitate-maleimide conjugated FITC-p62 (279–300) peptide (right panel). The retention time of FITC-p62 (279-300) peptide is 14.35 min, and that of FITC-p62 (279–300)-palm peptide is 27.18 min. Due to its high hydrophobicity, the peptide modified with two palmitates was not purified. We used the peptide modified with one palmitate for GUV experiment. FITC-p62 (279–300)-palm, palmitate-maleimide conjugated FITC-p62 (279–300).

(**C**) Mass spectrometry analysis of FITC-p62 (279–300) and FITC-p62 (279–300)-palm. MS (MALDI–TOF): FITC-p62 (279–300), calcd. for $C_{113}H_{164}N_{29}O_{43}S_3$ $[M+H]^+$ 2711.0622, found 2711.0425; FITC-p62 (279–300)-palm, calcd. for $C_{133}H_{199}N_{30}O_{45}S_3$ $[M+H]^+$ 3037.5847, found 3037.3289.

(**D**) SDS-PAGE analysis of purified MBP-mCherry-p62 (155–440)^{C331S}, MBP-GFP^{C48S}-p62 (155–440)^{C331S} and MBP-RavZ. The arrow indicates the target proteins.

(**E**) (Upper panel) SDS-PAGE analysis of p62 and palmitate-maleimide conjugation reaction. MBP-mCherry/GFP–p62 proteins were treated with 2 μM palmitate-maleimide (Mal-palm), the resulting product was further treated with 5 μM maleimide-poly (ethylene glycol) (5 kD) (Mal-PEG). (Lower panel) The Mal-PEG conjugated to p62 caused an up-shift of p62 protein (Lane 2), while palmitate-maleimide was incapable to change the p62 migration (Lane 3). Once palmitate-maleimide blocked the free –SH group, Mal-PEG didn't provide the up-shift of p62 any more (Lane 4). GFP-p62, MBP-GFP^{C48S}-p62 (155–440)^{C331S}; GFP-p62-palm, palmitate-maleimide conjugated MBP-GFP^{C48S}-p62 (155–440)^{C331S}.

(**F**) Representative images of FITC-p62 (279–300)-positive GUVs. The FITC-p62 (279–300) or FITC-p62 (279–300)-palm (green) was incubated with rhodamine labelled GUVs (red) for 0–60 min. The fluorescence was monitored by laser scanning confocal microscope and analyzed by OLYMPUS OlyVIA software. Scale bar, 4 μm.

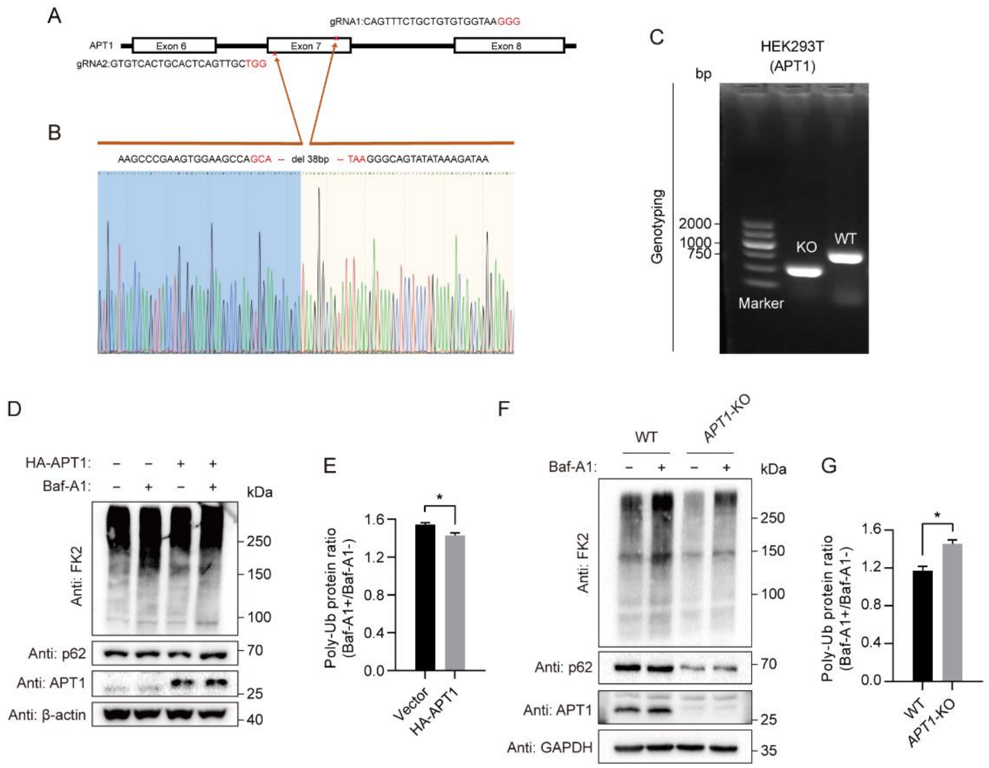

Figure S7. APT1 inhibits selective autophagic flux, related to Figure 7

(**A**) Deleting *APT1* in HEK293T cells. Targeting scheme of the truncation of exon 7 in human *APT1*. Two sgRNAs were designed to target the enzyme activity center (GTGTCACTGCACTCAGTTGC, CAGTTTCTGCTGTGTGGTAA) of human *APT1*.

(**B**) Sequencing verification of the *APT1*-KO HEK293T cell line. The knockout allele has a deletion of 38 bp on exon 7, confirmed by sequencing, and the ORF finder predicts that the knockout allele results in a premature termination of the protein.

(**C**) Electrophoresis verification of the *APT1*-KO HEK293T cell line. Genotyping was carried out by PCR amplification with corresponding primers, and the amplicons of WT and knockout alleles were 297 bp and 259 bp, respectively.

(**D**) Immunoblot analysis of total poly-ubiquitinated proteins in HEK293T cells transiently expressing HA-APT1 with or without Baf-A1 treatment for 18 h. Total proteins were extracted and detected by Western blotting.

(**E**) The ratio of the intensity of ubiquitinated proteins in Baf-A1-treated cells to that in Baf-A1-untreated cells as in (**D**). Data represent the mean ± SEM of three independent experiments. *$P < 0.05$, Student's t test.

(**F**) Immunoblot analysis of total poly-ubiquitinated proteins in WT or *APT1*-KO HEK293T cells with or without Baf-A1 treatment for 18 h.

(**G**) The ratio of the intensity of ubiquitinated proteins in Baf-A1-treated cells to that in Baf-A1-untreated cells as in (**F**). Data represent the mean ± SEM of three independent experiments. *$P < 0.05$, Student's *t* test.
